# Chemoenzymatic C,C-Bond Forming Cascades by Cryptic Vanadium Haloperoxidase Catalyzed Bromination

**DOI:** 10.1021/acs.orglett.4c04108

**Published:** 2024-12-31

**Authors:** Qingqi Zhao, Ru Zhang, Johannes Döbber, Tanja Gulder

**Affiliations:** † Biomimetic Catalysis, Catalysis Research Center, TUM School of Natural Sciences, Technical University of Munich, Lichtenbergstrasse 4, 85748 Garching, Germany; ‡ Institute of Organic Chemistry, Faculty of Chemistry and Mineralogy, Leipzig University, Johannisallee 29, 04103 Leipzig, Germany; § Organic Chemistry−Biomimetic Catalysis, Saarland University, 66123 Saarbruecken, Germany; ∥ Forschungszentrum Jülich GmbH, IBG-1: Biotechnology, Wilhelm-Johnen-Straße, 52428 Jülich, Germany; ⊥ Synthesis of Natural-Product Derived Drugs, Helmholtz Institute for Pharmaceutical Research Saarland (HIPS) Helmholtz Centre for Infection Research (HZI), 66123 Saarbruecken, Germany

## Abstract

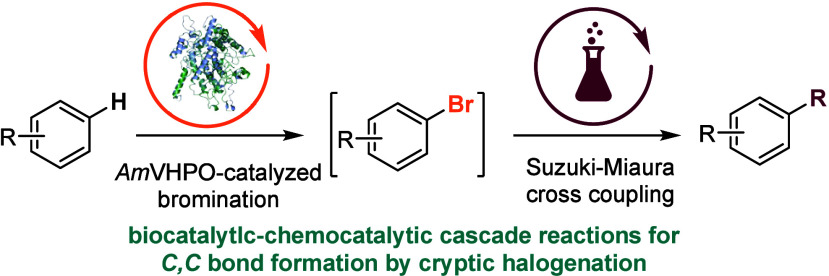

Inspired by natural cryptic halogenation in *C,C*-bond formation, this study developed a synthetic approach combining biocatalytic bromination with transition-metal-catalyzed cross-coupling. Using the cyanobacterial *Am*VHPO, a robust and sustainable bromination-arylation cascade was created. Genetic modifications allowed enzyme immobilization, enhancing the compatibility between biocatalysis and chemocatalysis. This mild, efficient method for synthesizing biaryl compounds provides a foundation for future biochemo cascade reactions harnessing halogenation as a traceless directing tool.

Halogen-containing compounds are well-known for their vast applications in pharmaceutical and agricultural industries.^1^ In organic synthesis, installing a halogen atom can open a plethora of further transformations, particularly carbon–carbon (*C*,*C*)coupling reactions. Nature also takes advantage of the beneficial properties of *C*,*X*-bonds and uses them as a strategic tool for assembling structurally complex frameworks.^2^ Biochemical reactions triggered by cryptic halogenations have recently been observed in the biosynthesis of cyclindrophanes **1** (Friedel–Crafts alkylation),^3^ marinopyrrole A (**2**, *C*,*N*-biaryl coupling),^4^ and merochlorine D (**3**, α-hydroxy-ketone rearrangement).^5^ By harnessing in situ generated *C*,*X*-bonds for *C*,*C*-bond formations, highly efficient multistep sequences are performed in a straightforward way.

Inspired by nature’s synthetic strategy, our group aims to emulate such processes.^6^ In this article, we establish halogenation-triggered cascade reactions as a tool in organic synthesis by integrating *AmVHPO-*catalyzed bromination in a one-pot reaction with a Pd-catalyzed cross-coupling reaction. Such pathways are highly rewarding,^7^ as they offer improved step economics^8^ while reducing resources compared to conventional organic synthesis. Integrating biocatalytic processes into chemosynthetic regimes offers multiple advantages.^9^ However, successfully integrating enzymes with chemocatalysts is still challenging because of their inherently different operating conditions, often requiring compartmentalization of at least one of the catalysts.^10^ In chemical synthesis, halogenations of sp^2^-carbons proceed mostly via electrophilic halogenations using stoichiometric amounts of either toxic, often difficult-to-handle, corrosive dihalogens or organic electrophilic reagents that produce large quantities of organic waste. Biocatalytic halogenations serve as an exciting alternative.^11^ Haloperoxidases constitute here a promising enzyme class because of their ability to convert a broader substrate range and their cost- and atom-economic cofactors vanadate and H_2_O_2_ (Figure 1b).^12^ These characteristics inherently position VHPOs as versatile choices for catalytic applications in biotechnology.

**Figure 1:**
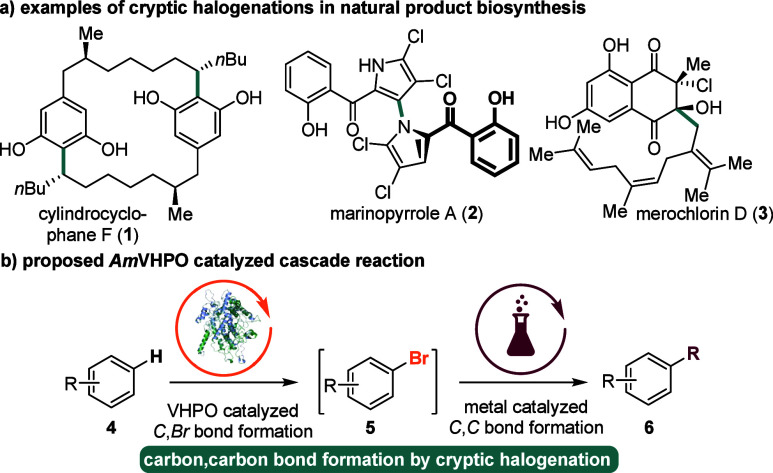
Cryptic halogenations (a) in nature during the synthesis of cyclindrophanes, marinopyrroles, and merochlorines and (b) their application in the arylation and alkenylation of (hetero)aromatic compounds.

Vanadium-dependent haloperoxidases (VHPO) have lately gained recognition for their applications in organic synthesis,^13^ even catalyzing reactions beyond their natural scope. The catalytic oxidative bromination requires cheap halide salts, sodium vanadate as a prosthetic group, and H_2_O_2_ as an oxidant, providing a cost-effective and atom-economic system. Our group has previously investigated the haloperoxidase *Am*VHPO from the cyanobacterium *Acaryochloris marina.*(13i,^14^)*Am*VHPO exhibits exceptional robustness toward organic solvents, temperature, and H_2_O_2_, making it ideally suited for its application in organic transformations.^13h,13i^ We established a protocol for *Am*VHPO-catalyzed brominations of diverse aromatic compounds, achieving good to excellent yields and selectivities. Combining *Am*VHPO with photochemically generated H_2_O_2_ gave access to even milder reaction conditions and further showed the potential of this biocatalyst.^13h,13,15^

Generally, fundamental differences must be overcome between enzymatic transformations and transition-metal catalysis to effectively combine a Pd-catalyzed cross-coupling reaction with an enzymatic transformation. The aqueous environment and high dilution needed for enzymatic reactions are vastly untypical for most chemical conversions. For the enzymatic bromination with *Am*VHPO, the highest conversion was achieved at 7.5 mM,^13h,13i^ a substrate concentration substantially lower than the concentration used in most organic reactions. In our study, we mainly focused on converting (hetero)aromatic compounds since these are prevalent structural motifs found ubiquitously in bioactive drugs and pharmaceuticals.^16^ Therefore, we started our investigations by selecting indole **5a** as our model substrate. As an optimized procedure for *Am*VHPO-triggered aromatic bromination has already been established before,^13h,13i^ we first focused on adjusting the reaction conditions of the Suzuki–Miyaura cross-coupling to those needed for the enzymatic bromination. Initially, screenings were performed in a highly diluted (7.5 mM), degassed aqueous environment, employing different Pd catalysts, ligands, and bases (Table 1 and Supporting Information (SI)). Product **6a** was afforded in quantitative yields using the Pd(II) catalysts PdCl_2_(PPh_3_)_2_ and Pd(OAc)_2_ in combination with the water-soluble trisodium 3,3′,3″-phosphanetriyltri(benzene-1-sulfonate) (TPPTS) ligand and K_2_CO_3_ as a base at 50 °C (Table 1, entries 3 and 4). Because of the superior solubility in the water/acetonitrile (50:50) mixture, further investigations were conducted with Pd(OAc)_2_.

**Table 1:**
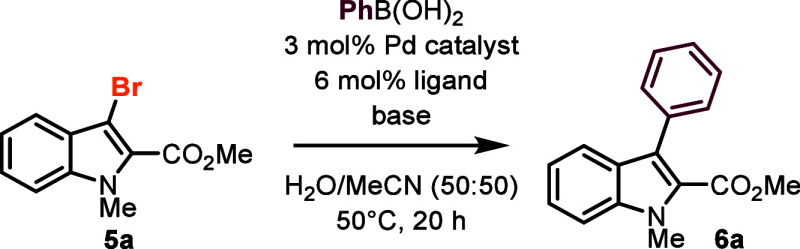
Optimization of the Suzuki–Miyaura Cross-Coupling Reaction in Water/Acetonitrile^a^

entry	Pd catalyst	base	ligand^d^	yield^b^ [%]
1	Na_2_PdCl_4_	K_2_CO_3_	–	58
2	Pd(PPh_3_)_4_	K_2_CO_3_	–	68
3	PdCl_2_(PPh_3_)_2_^c^	K_2_CO_3_	TPPTS	quant.
4	Pd(OAc)_2_	K_2_CO_3_	TPPTS	quant.
5	Pd(OAc)_2_	K_3_PO_4_	TPPTS	59
6	Pd(OAc)_2_	K_2_CO_3_	TPPTS	85

a The reactions were carried out using 3-bromoindole **5a** (12.0 μmol, 1.0 equiv), PhB(OH)_2_ (14.4 μmol, 1.2 equiv), ligand (0.72 μmol, 0.06 equiv), base (0.24 mmol, 2.0 equiv), and Pd catalyst (0.36 μmol, 0.03 equiv) in 1.6 mL degassed solvent mixture (50:50, 7.5 mM) at 50 °C for 20 h.

b The yield was determined by GC MS from the crude mixture using dodecane as internal standard.

c Suspension in H_2_O and MeCN.

d Solvent mixture not degassed. TPPTS = trisodium 3,3′,3″-phosphanetriyltri(benzene-1-sulfonate).

The optimized aqueous conditions needed further adjustments when reacting in buffered solutions. An increase in pH was needed when turning from biocatalytic bromination to Pd-catalyzed *C,C*-coupling. While the *Am*VHPO requires pH 6.0 to achieve optimal halogenation activity, basic conditions are warranted for the Suzuki–Miyaura reaction. Therefore, the optimum pH for the second step was evaluated by varying the amount of K_2_CO_3_ (Figure 2a). The best yields were obtained using 20 equiv of K_2_CO_3_ at pH 10 (56%). Subsequent screening of bases showed that with an excess of NEt_3_, the buffer capacity was likewise overcome (pH 10), and **6a** was isolated in 90% yield (Figure 2b, cf. SI chapter S4).

**Figure 2:**
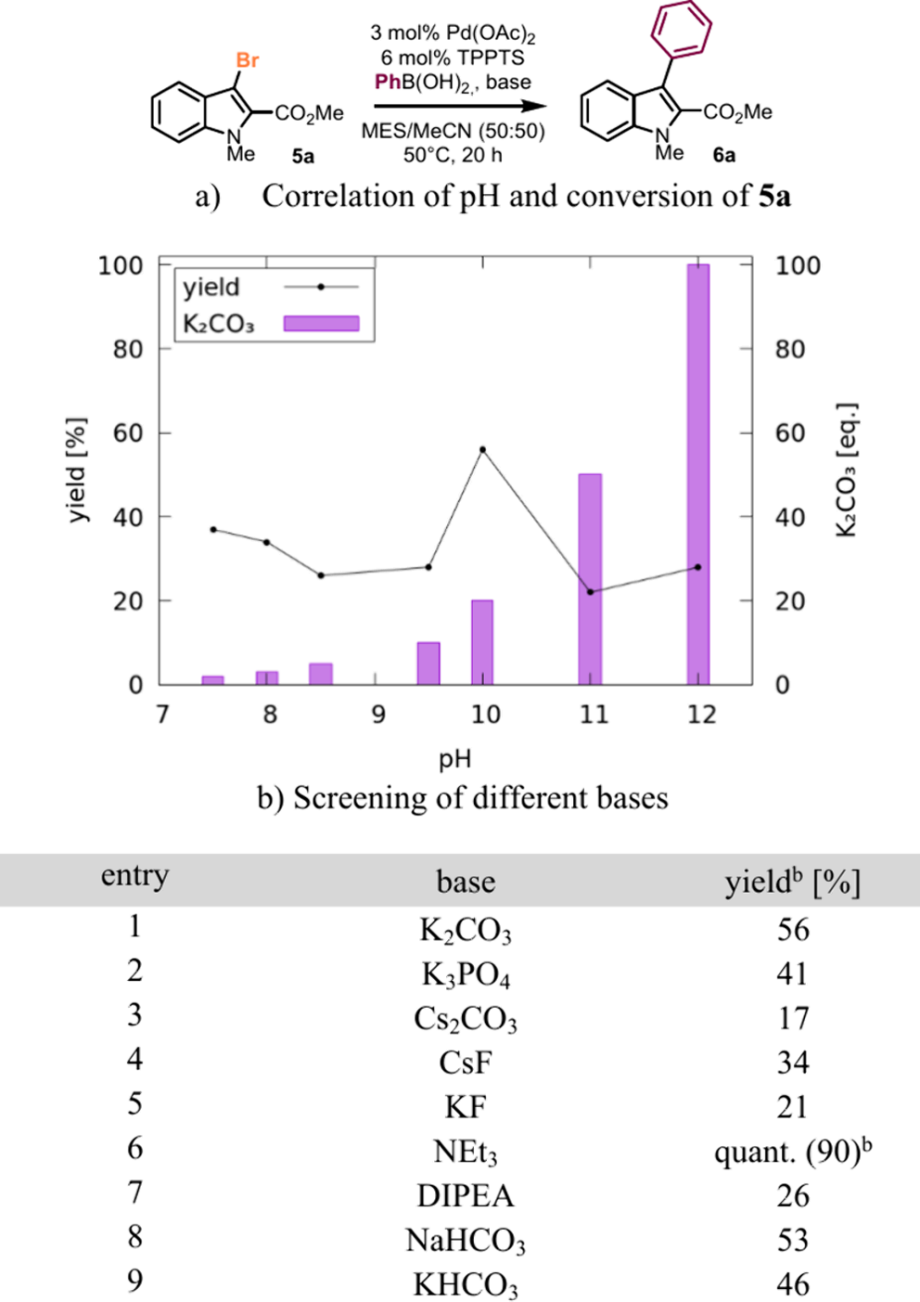
Influence of the base on the cross-coupling reaction.^a a^The reactions were carried out using 3-bromoindole **5a** (12.0 μmol, 1.0 equiv), PhB(OH)_2_ (1.2 equiv), TPPTS (0.06 equiv), and Pd(OAc)_2_ (0.03 equiv) in degassed MES buffer (pH 6.0, 50 mM) and MeCN (50:50, v/v, 7.5 mM) at 50 °C for 20 h. (a) Influence of pH on the conversion of the aqueous Pd-catalyzed cross-coupling reaction in MES buffer with 2–100 equiv K_2_CO_3_ as base. (b) Screening of different bases using 20 eq base. ^a^GC yield was determined from the crude using dodecane as the internal standard. ^b^Isolated yield. MES = 2-(*N*-morpholino)ethanesulfonic acid, DIPEA = diisopropylamine, TPPTS = trisodium 3,3′,3″-phosphanetriyltri(benzene-1-sulfonate).

Next, we tested the compatibility of the *C,C*-bond forming reaction with enzymatic bromination (see Table 2). In our initial attempt using soluble *Am*VHPO,^13h,13i^ we observed no conversion to the desired cross-coupling product **6a** (Table 2, entry 1). This lack of conversion may be attributed to the inhibitory effect of Lewis basic amino acid side chains within the protein such as those in cysteines and histidines. Such moieties are known to coordinate the metal and thus hinder its catalytic ability.^17^ To address this problem, we covalently attached the biocatalyst to solid support.^18^ Therefore, we genetically fused a HaloTag construct into *Am*VHPO^19^ and immobilized the tagged enzyme with the commercially available HaloLink resin.^19c^ For details on cloning, protein production, purification, immobilization protocol, and enzyme loading, refer to SI 3.1 and 3.3 and 3.4, respectively. Despite *Am*VHPO’s huge size due to its dodecameric structure, *Am*VHPO loading could be increased to 0.51 ± 0.03 mg mg^–1^. The *Am*VHPO_HaloTag_ complex exhibited comparable bromination activity in the standard monochlorodimedon assay to the soluble HisTagged enzyme (see SI chapter S3.3). After immobilizing *Am*VHPO_HaloTag_ onto HaloLink resin (*Am*VHPO_immo_), the enzyme did not interfere with the transition metal catalyst after simply centrifuging the reaction mixture after the enzymatic bromination step. This procedure increased the overall yield to 41% (Table 2, entry 2). Another increase in yield (52% and 69% from **4a**, respectively) was observed by adding additional portions of palladium, ligand, and boronic acid after each hour (Table 2, entries 3 and 4). Such observations hint at a decomposition of these reactants over time under the applied conditions. This behavior could be caused by residual H_2_O_2_ from the bromination step that interferes with the Pd species through oxidation. As lowering the H_2_O_2_ concentration diminished the yield of brominated indole **5a**, we set out to destroy the residual oxidant from the reaction mixture before adding the Pd species. The addition of reductants, such as MnO_2_ (10 mol %) or FeCl_3_ (1.1 equiv)_,_ and incubating the reaction mixture for another hour afforded the final product **6a** in quantitative and 94% overall yield, respectively (Table 2, entries 5 and 6). As MnO_2_ needed to be employed only in catalytic amounts, it was used for all further transformations.

**Table 2:**
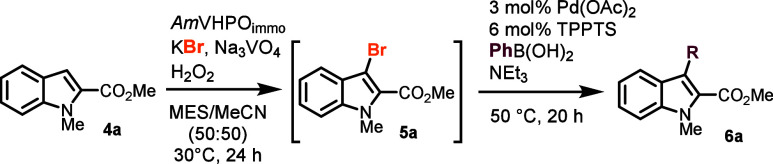
Optimization of the Cascade Reaction Combining Enzymatic Bromination and a Pd-Catalyzed Cross-Coupling Reaction^a^

entry	enzyme	H_2_O_2_ deactivation	yield^b^ [%]
1	*Am*VHPO	–	–
2	*Am*VHPO_immo_^c^	–	41
3^d^	*Am*VHPO_immo_^c^	–	52
4^e^	*Am*VHPO_immo_^c^	–	69
5	*Am*VHPO_immo_^c^	10 mol % MnO_2_, 30 °C, 1 h	quant.
6	*Am*VHPO_immo_^c^	1.1 equiv FeCl_3_, rt, 1 h	94

a The reactions were carried out using indole **4a** (7.50 mM; 12.0 μmol), KBr (1.1 equiv), H_2_O_2_ (1.1 equiv), Na_3_VO_4_ (190 μM), *Am*VHPO (50 μL; 3 mg mL^–1^; 6 U; preincubated with 300 μL of 30 mM K_3_VO_4_) in MES buffer (pH 6.0, 50 mM) and MeCN (1:1, 7.5 mM, total volume = 1.6 mL) were shaken at 30 °C and 1200 rpm for 24 h. After degassing the reaction mixture, boronic acid (1.2 equiv), TPPTS (0.06 equiv), NEt_3_ (20 equiv), and Pd(OAc)_2_ (0.03 equiv) were added, and the reaction mixture was stirred at 50 °C for 20 h. .

b GC yield determined from the crude reaction mixture using dodecane as an internal standard.

c HaloTag-HaloLink *Am*VHPO complex immobilized on HaloLink resin.

d Additional 3 mol % Pd(OAc)_2_ and 6 mol % TPPTS were added after 1 h.

e Additional 3 mol % Pd(OAc)_2_, 6 mol % TPPTS, and 1.2 equiv of PhB(OH)_2_ were added after 1 h and 2 h. MES = 2-(*N*-morpholino)ethanesulfonic acid; TPPTS = trisodium 3,3′,3″-phosphanetriyltri(benzene-1-sulfonate).

With a working one-pot method on hand, the scope of the reaction was explored. Regarding boronic acids, various substrates were compatible with our transformation (Scheme 1). Both electron-rich and electron-poor aryl boronic acids could be employed, giving the corresponding products **6a**–**6t** in up to quantitative yields. Only the *p*-nitro-benzene **6l** showed a decreased yield of 59% due to solubility issues of the boronic acid. Furthermore, the sterically hindered *ortho-*chlorinated and *ortho-*methylated boronic acid resulted in a 58% and 45% yield of indoles **6j** and **6m**, respectively. Heterocyclic boronic acids were also successfully converted. For instance, furanyl boronic acid yielded indole **6p** with an impressive 96% yield, whereas boronic acids with 4-pyridyl and 2-benzothiophene functionalities showed binding to the protein, thus hampering the conversion of intermediate **5a**. Nevertheless, the biaryl products **6n** and **6o** were obtained in 71% and 66% yield based on the recovered intermediate (bri) **6a**. Simple cyclohexenyl boronic acids afforded product **6s** in 94% isolated yield, and even the styrenyl derivative produced the desired product **6t** in 36% (81% bri) despite the styrenyl reactant’s tendency to undergo polymerization.

**Scheme 1:**
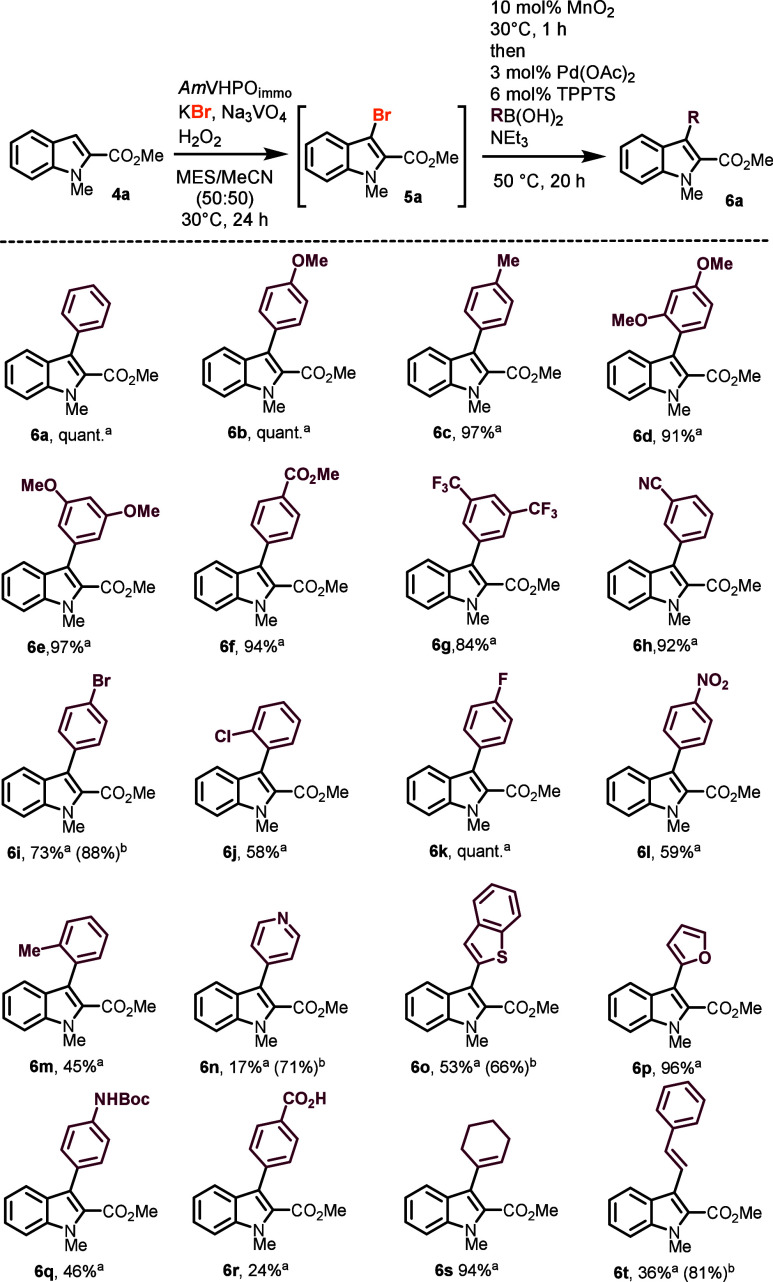
Substrate Scope: Variation of the Boronic Acid Moiety Isolated yield. Yield based on recovered bromoindole **5a**. MES = 2-(*N*-morpholino)ethane sulfonic acid; TPPTS = trisodium 3,3′,3″-phosphanetriyltri(benzene-1-sulfonate).

The versatility of the cascade reaction prompted us to expand our exploration to include starting materials beyond the 2-carboxylic methyl ester indole **4a** (Scheme 2).^13h,13i^ For instance, *N*-methylated indole **4u** was seamlessly converted to the desired monoarylated product **6u** in 57% yield. Despite the inherent instability of substrate **4u** and its brominated derivative **5u**, we did not observe any dibromination or oxidative decomposition of the indole moiety, underscoring the mildness of the reaction conditions. Indoles lacking substituents at the nitrogen atom were transformed into the corresponding products **6v** and **6w** only when electron-withdrawing substituents were positioned at C2. The biocatalytic cascade reaction was applicable to other heterocyclic substrates such as pyrroles (→**6x**), carbazoles (→**6y**), and thiophenes (→**6z**). The latter stood out as the electron-rich bis methoxylated thiophene **4z** forms a delicate, acid- and heat-sensitive brominated intermediate **5z** that is tough to handle in stepwise transformations. Notably, electron-rich benzene derivatives served as substrates, giving the biaryl products **6aa** and **6ab** in 94% and 64% yield, respectively. Also, monoterpene carvacrol (**4ac**) was successfully converted to corresponding biphenyl **6ac**.

**Scheme 2:**
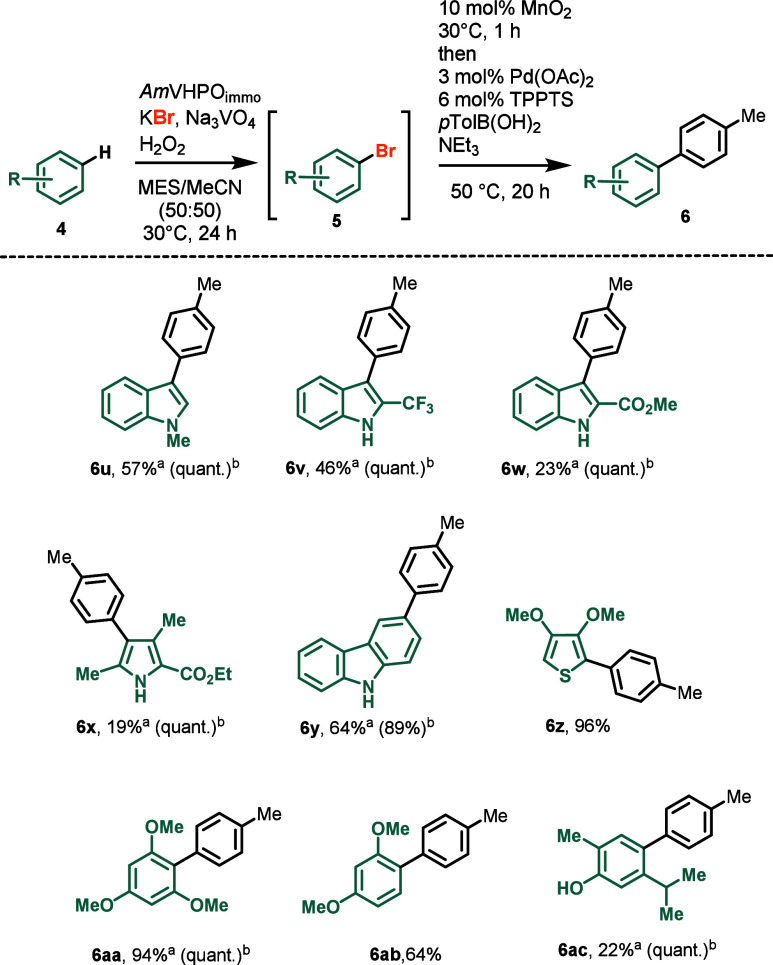
Substrate Scope: Variation of the Aryl Moiety Isolated yield. Yield based on recovered starting material **4**. MES = 2-(*N*-morpholino)ethane sulfonic acid; TPPTS = trisodium 3,3′,3″-phosphanetriyltri(benzene-1-sulfonate).

Nature provides numerous examples of cryptic halogenations in complex natural product synthesis as an efficient way to forge *C,C* bonds. Inspired by this natural strategy, we elaborated a synthetic method combining biocatalytic bromination with transition-metal catalyzed cross-coupling reactions. In our setup, the cyanobacterial haloperoxidase *Am*VHPO was employed to activate the C–H bond through cryptic halogenation assisting *C,C* bond formation in a bromination-arylation cascade. The employed biocatalytic system is robust, simple, and easy to handle, thus making it a cost-effective, atom-economical, and sustainable alternative for halogenations. Simple genetic modification of the *Am*VHPO was applied to enable enzyme immobilization on resin to overcome incompatibility issues between the biocatalytic and chemocatalytic steps. The two-step-one-pot procedure presented here is proof of principle for the versatility of vanadium-dependent haloperoxidases in chemoenzymatic transformations and the enzymes’ compatibility with organic synthetic setups. Overall, the mild, generally applicable, and efficient method for converting various electron-rich (hetero)aromatic scaffolds into biaryl compounds laid the foundation for future biocascade reactions, ranging from other transition-metal catalyzed transformations to photocatalytic and electrochemical reactions.

## Data Availability

The data underlying this study are available in the published article and its Supporting Information.

## Supporting Information Available

Experimental procedures, characterization of all compounds, and further information on the substrate scope are presented in the Supporting Information. (PDF)

## References

[ref1] aHernandes M. Z.; Cavalcanti S. M. T.; Moreira D. R. M.; de Azevedo J.; Filgueira W.; Leite A. C. L. Halogen atoms in the modern medicinal chemistry: hints for the drug design. Curr. Drug Targets 2010, 11, 303–314. 10.2174/138945010790711996. 20210755

[ref2] Adak S.; Moore B. S. Cryptic halogenation reactions in natural product biosynthesis. Nat. Prod. Rep. 2021, 38, 1760–1774. 10.1039/D1NP00010A. 34676862 PMC8542889

[ref3] Nakamura H.; Schultz E. E.; Balskus E. P. A new strategy for aromatic ring alkylation in cylindrocyclophane biosynthesis. Nat. Chem. Biol. 2017, 13, 916–921. 10.1038/nchembio.2421. 28671684

[ref4] Yamanaka K.; Ryan K. S.; Gulder T. A. M.; Hughes C. C.; Moore B. S. Flavoenzyme-Catalyzed Atropo-Selective N,C-Bipyrrole Homocoupling in Marinopyrrole Biosynthesis. J. Am. Chem. Soc. 2012, 134, 12434–12437. 10.1021/ja305670f. 22800473 PMC3415713

[ref5] Miles Z. D.; Diethelm S.; Pepper H. P.; Huang D. M.; George J. H.; Moore B. S. A unifying paradigm for naphthoquinone-based meroterpenoid (bio) synthesis. Nat. Chem. 2017, 9, 1235–1242. 10.1038/nchem.2829. 29168495 PMC5960991

[ref6] aArnold A. M.; Dullinger P.; Biswas A.; Jandl C.; Horinek D.; Gulder T. Enzyme-like polyene cyclizations catalyzed by dynamic, self-assembled, supramolecular fluoro alcohol-amine clusters. Nat. Commun. 2023, 14, 813 10.1038/s41467-023-36157-0. 36781877 PMC9925744

[ref7] aPatzelt C.; Poethig A.; Gulder T. Iodine(III)-Catalyzed Cascade Reactions Enabling a Direct Access to β-Lactams and α-Hydroxy-β-amino Acids. Org. Lett. 2016, 18, 3466–3469. 10.1021/acs.orglett.6b01658. 27380445

[ref8] Wender P. Towards the ideal synthesis. Chemistry and Industry 1997, 765.

[ref9] aHessefort L. Z.; Harstad L. J.; Merker K. R.; Ramos L. P. T.; Biegasiewicz K. F. Chemoenzymatic Catalysis: Cooperativity Enables Opportunity. ChemBioChem. 2023, 24, e202300334 10.1002/cbic.202300334. 37252875

[ref10] Latham J.; Henry J.-M.; Sharif H. H.; Menon B. R.; Shepherd S. A.; Greaney M. F.; Micklefield J. Integrated catalysis opens new arylation pathways via regiodivergent enzymatic C–H activation. Nat. Commun. 2016, 7, 1–8. 10.1038/ncomms11873. PMC4906404 27283121

[ref11] aAgarwal V.; Miles Z. D.; Winter J. M.; Eustáquio A. S.; El Gamal A. A.; Moore B. S. Enzymatic halogenation and dehalogenation reactions: pervasive and mechanistically diverse. Chem. Rev. 2017, 117, 5619–5674. 10.1021/acs.chemrev.6b00571. 28106994 PMC5575885

[ref12] aSchnepel C.; Sewald N. Enzymatic halogenation: a timely strategy for regioselective C–H activation. Chem.—Eur. J. 2017, 23, 12064–12086. 10.1002/chem.201701209. 28464370

[ref13] aLi H.; Younes S. H. H.; Chen S.; Duan P.; Cui C.; Wever R.; Zhang W.; Hollmann F. Chemoenzymatic Hunsdiecker-Type Decarboxylative Bromination of Cinnamic Acids. ACS Catal. 2022, 12, 4554–4559. 10.1021/acscatal.2c00485. 35465241 PMC9016706

[ref14] aZeides P.; Bellmann-Sickert K.; Zhang R.; Seel C. J.; Most V.; Schoeder C.; Groll M.; Gulder T. Unraveling the Molecular Basis of Substrate Specificity and Halogen Activation in Vanadium-Dependent Haloperoxidases. ChemRixv 2023, 10.26434/chemrxiv-22023-26437l26430h. PMC11871015 40021637

[ref15] Seel C. J.; Gulder T. Biocatalysis fueled by light: on the versatile combination of photocatalysis and enzymes. ChemBioChem. 2019, 20, 1871–1897. 10.1002/cbic.201800806. 30864191

[ref16] aSrivastava A.; Pandeya S. Indole: a versatile nucleuse in pharmaceutical field. Int. J. Curr. Pharm. Rev. Res. 2011, 4, 5–8.

[ref17] Kilpin K. J.; Dyson P. J. Enzyme inhibition by metal complexes: concepts, strategies and applications. Chem. Sci. 2013, 4, 1410–1419. 10.1039/c3sc22349c.

[ref18] Sheldon R. A.; van Pelt S. Enzyme immobilisation in biocatalysis: why, what and how. Chem. Soc. Rev. 2013, 42, 6223–6235. 10.1039/C3CS60075K. 23532151

[ref19] aLos G. V.; Wood K. The HaloTag. In High Content Screening; Springer, 2007; pp 195–208.

