# Supplementary material for: Chemoenzymatic C,C-Bond Forming Cascades by Cryptic Vanadium Haloperoxidase Catalyzed Bromination
Source: Org Lett. 2024 Dec 31;27(1):159–64. doi: 10.1021/acs.orglett.4c04108 (PMC11731374; doi:10.1021/acs.orglett.4c04108)
Supplement: Supplementary file 1 — ol4c04108_si_001.pdf [file ol4c04108_si_001.pdf]

# - SUPPORTING INFORMATION –

Chemoenzymatic C,C-Bond Forming Cascades by Cryptic Vanadium Haloperoxidase Catalyzed Bromination

Qingqi Zhao,[a] Ru Zhang,[b,c] Johannes Döbber,[d] Tanja Gulder[a,b,c,e*]

[a]Biomimetic Catalysis, Catalysis Research Center, TUM School of Natural Sciences, Technical University of Munich, Lichtenbergstrasse 4, 85748 Garching, Germany

[b]Institute of Organic Chemistry, Faculty of Chemistry and Mineralogy, Leipzig University, Johannisallee 29, 04103 Leipzig, Germany

[c]Organic Chemistry – Biomimetic Catalysis, Saarland University, 66123 Saarbruecken, Germany

[d]Forschungszentrum Jülich GmbH, IBG-1: Biotechnology, Wilhelm-Johnen-Straße, 52428 Jülich, Germany

[e]Synthesis of Natural-Product Derived Drugs, Helmholtz Institute for Pharmaceutical Research Saarland (HIPS) Helmholtz Centre for Infection Research (HZI), 66123 Saarbruecken, Germany

corresponding author: tanja.gulder@uni-saarland.de

Content

Content 2

1. General Information 3

2. Optimization of Suzuki Cross-Coupling Reaction in MES Buffer and MeCN 5

3. Cloning, Protein Production, and Purification of *Am*$VHPO_{HaloTag}$ 8

3.1 Heterologous Production of *Am*$VHPO_{HaloTag}$ 8

3.2 Sequence of the pET22-HaloTag-*Am*VHPO plasmid 9

3.3 Determination of the *Am*$VHPO_{wild\text{-}type}$ vs *Am*$VHPO_{HaloTag}$ Activity using MCD Activity Assay[1] 12

3.4 Immobilization of *Am*$VHPO_{HaloTag}$ on HaloLink Resin[2] 13

3.5 Bicinchoninic acid (BCA) Assay for the Determination of Enzyme Loading[3] 14

4. Procedures 15

4.1 General Procedure (GP-1) for the Enzymatic Synthesis of Brominated Indoles 15

4.2 General Procedure (GP-2) for the Synthesis of One-Pot Reactions Combining Enzymatic Bromination and Suzuki Cross-Coupling Reaction 15

4.3 Synthesis of Product **6a** on a mmol Scale 15

5. Physical and Spectroscopic Data 17

5.1 Physical and Spectroscopic Data of Starting Material and Brominated Intermediate 17

5.2 Physical and Spectroscopic Data of the Products **6** 18

6. NMR Spectra of Compounds 28

7. References 62

1. General Information

Solvents used in reactions were p.a. grade. Solvents for chromatography were technical grade and distilled before use. Reagents were purchased at the highest commercial quality and used without further purification. Reactions were monitored by thin layer chromatography (TLC) on Merck silica gel aluminum plates with an F-254 indicator using UV light as the visualizing agent. Reactions were monitored by thin layer chromatography (TLC) carried out on Merck silica gel aluminum plates with an F-254 indicator using UV light as the visualizing agent (UV) and a basic potassium permanganate solution (KMnO4) and heat as the developing agent. Silica gel Merck 60 (particle size 0.63 – 0.2 mm) was used for flash column chromatography. Solvent mixtures are reported as volume/volume (v/v). NMR spectra were recorded on Bruker AV300, Bruker AV400, or Bruker AV500 spectrometers. The spectra were calibrated using residual undeuterated solvents as internal references ($CHCl_3$ @ 7.26 ppm ^{1}H NMR and $CHCl_3$ @ 77.00 ppm ^{13}C NMR). The following abbreviations (or combinations thereof) are used to explain the multiplicities: s = singlet, d = doublet, dd = doublet of doublets, t = triplet, dt = doublet of triplets, q = quartet, m = multiplet, br = broad. In addition, the following abbreviations are used: *Am*VHPO = *Acaryochloris marina*, BCA = bicinchoninic acid, BSA = bovine serum albumin, calcd. = calculated, $CDCl_3$ = deuterated chloroform, Cs_2CO_3 = cesium carbonate, CsF = cesium fluoride, CsOAc = cesium acetate, DIPEA = *N*,*N*-diisopropylethylamine, DMSO = dimethyl sulfoxide, ESI = electrospray ionization, GC = gas chromatography, H_2O_2 = hydrogen peroxide, HPLC = high performance liquid chromatography, HRMS = high resolution mass spectrometry, immo = immobilized, IR = infrared spectroscopy, K_2CO_3 = potassium carbonate, K_3PO_4 = potassium phosphate, KBr = potassium bromide, KF = potassium flouride, KH_2PO_4 = potassium dihydrogen phosphate, $KHCO_3$ = potassium bicarbonate, KOAc = potassium acetate, KOH = potassium hydroxide, KO*t*Bu = potassium tert-butoxide, λ = wave length, LB = lysogeny broth,MeI = methyl iodide, m.p. = melting point, MCD = monochlorodimedone, MeCN = acetonitrile, MES = 2-(*N*-morpholino)ethanesulfonic acid, MnO_2 = manganese dioxide, MS = mass spectrometry, Na_2PdCl_4 = sodium tetrachloropalladate(II), Na_2SO_4 = sodium sulfate, Na_3VO_4 = sodium orthovanadate, NaCl = sodium chloride, NaH_2PO_4 = sodium dihydrogen phosphate, $NaHCO_3$ = sodium bicarbonate, NaOAc = sodium acetate, NaOH = sodium hydroxide, NaO*t*Bu = sodium tert-butoxide, NEt_3 = triethylamine, $(NH_4)_2SO_4$ = ammonium sulfate, NMR = nuclear magnetic resonance, PCR = polymerase chain reaction, Pd = palladium, $Pd(OAc)_2$ = palladium acetate, $Pd(PPh_3)_4$ = palladium-tetrakis(triphenylphosphine), $PdCl_2(PPh_3)_2$ = bis(triphenylphosphine)palladium(II) dichloride, $PhB(OH)_2$ = phenylboronic acid, quant. = quantitative, R_f = retardation factor, rpm = revolutions per minute, rt = room temperature, TBAB = tetra-*n*-butylammonium bromide, THF = tetrahydrofuran, TPPTS = triphenylphosphine-3,3',3''-trisulfonic acid trisodium salt, Tris = 2-amino-2-(hydroxymethyl)-1,3-propanediol. IR spectra were recorded on a JASCO FT-IR-4100 (ATR) and are reported in terms of frequency of absorption (cm^{-1}). GC-MS measurements were conducted on a Finnigan MAT SSQ 7000

(MS-EI, 70 eV; CI, 100 eV). HRESI-MS mass spectra were recorded on a Thermo LTQ FT Ultra using an ion trap and coupled to a Dionex UltiMate 3000 HPLC system. Gas chromatography (GC) measurements were carried out on an HP 6890 Series GC instrument (Agilent, equipped with an FID detector and an HP-5 capillary column [length = 29.5 m]). Hydrogen was used as the carrier gas and the constant-flow mode (flow rate = 1.8 mL min^{-1}) with a split ratio of 1:20. The following temperature program was used: 60°C for 3 min, 15°C min^{-1} to 250°C in 12 min, and 250°C for 5 min. High-performance liquid chromatography (HPLC) analysis was performed on a HITACHI Chromaster employing the following conditions: 150 x 4 mm Eurospher II (KNAUER®), 100 Å, 5 µm, C18, 25°C, flow rate: 1.0 mL min^{-1}, water (+0.01% TFA)/MeCN (+0.01% TFA) gradient: 90/10 → 0/100 in 30 min, hold 0/100 for 5 min, 0/100 → 10/90 in 1 min, hold 10/90 for 9 min. UV/Vis spectra were recorded on a BioTek® Eon spectrophotometer. MCD assays were performed in 96-well polystyrene microplates (Brand® F-bottom, UV-transparent, pureGrade™). Protein concentrations were determined using a BCA test kit purchased from interchim uptima.

2. Optimization of Suzuki Cross-Coupling Reaction in MES Buffer and MeCN

Table S1. Optimization of MES buffer concentration in the Suzuki-Miyaura cross-coupling reaction.

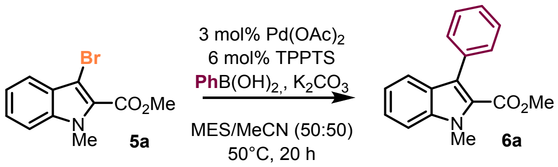

entry	MES concentration [mM]	yield[a] [%]
1	10	5
2	25	16
3	50	37
4	80	5
5	100	3

The reactions were carried out using 3-bromoindole **5a** (12.0 µmol, 1.0 eq), $PhB(OH)_2$ (14.4 µmol, 1.2 eq), TPPTS (0.72 µmol, 0.06 eq), K_2CO_3 (0.24 mmol, 2.0 eq) and $Pd(OAc)_2$ (0.36 µmol, 0.03 eq) in 1.6 mL degassed MES buffer (pH 6.0) and MeCN (50:50 v/v, 7.5 mM) at 50°C for 20 h. [a]GC yield determined from the crude reaction mixture using dodecane as internal standard.

Table S2. Optimization of the base equivalents for the Suzuki-Miyaura cross-coupling reaction.

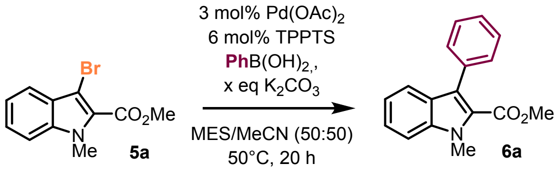

entry	K_2CO_3 [eq]	yield[a] [%]	pH value
1	2	37	7.5
2	3	34	8.0
3	5	26	8.5
4	10	28	9.5
5	20	56	10
6	50	22	11
7	100	28	12

The reactions were carried out using 3-bromoindole **5a** (12.0 µmol, 1.0 eq), $PhB(OH)_2$ (14.4 µmol, 1.2 eq), TPPTS (0.72 µmol, 0.06 eq), K_2CO_3 (eq indicated as above) and $Pd(OAc)_2$ (0.36 µmol, 0.03 eq) in 1.6 mL degassed MES buffer (pH 6.0, 50 mM) and MeCN (50:50, v/v, 7.5 mM) at 50°C for 20 h. [a]GC yield determined from the crude reaction mixture using dodecane as internal standard.

Table S3. Screening of different bases in the Suzuki-Miyaura cross-coupling reaction.

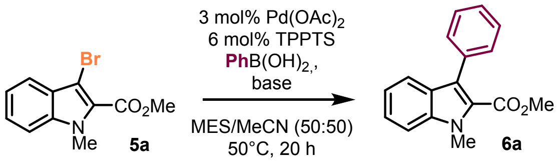

entry	base	yield[a] [%]	pH value
1	K_3PO_4	41	11.5
2	Cs_2CO_3	17	11.0
3	NaOH	< 1	10.5
4	KOH	< 1	11.0
5	KOAc	6	7.0
6	CsOAc	6	7.0
7	CsF	34	7.0
8	KF	21	7.0
9	NEt_3	quant. (90)[b]	10.5
10	DIPEA	26	9.5
11	$NaHCO_3$	53	10.0
12	$KHCO_3$	46	9.5
13	NaH_2PO_4	9	7.0
14	KH_2PO_4	7	7.0
15	KOtBu	1	11.5
16	NaOtBu	< 1	11.0

The reactions were carried out using 3-bromoindole **5a** (12.0 µmol, 1.0 eq), $PhB(OH)_2$ (14.4 µmol, 1.2 eq), TPPTS (0.72 µmol, 0.06 eq), base (0.24 mmol, 20 eq) and $Pd(OAc)_2$ (0.36 µmol, 0.03 eq) in 1.6 mL degassed MES buffer (pH 6.0, 50 mM) and MeCN (50:50, v/v, 7.5 mM) at 50°C for 20 h. [a]GC yield was determined from the crude reaction mixture using dodecane as the internal standard. [b]Isolated yield.

3. Cloning, Protein Production, and Purification of *Am*VHPO$_{\text{HaloTag}}$

3.1 Heterologous Production of *Am*VHPO$_{\text{HaloTag}}$

The genomic DNA from *Acaryochloris marina* (strain MBIC 11017) was used as a template for the PCR amplification of the isoenzyme *Am*VHPO (Genbank accession number WP_012165216) gene sequence. Applying restriction digest (BamHI, PstI) and ligation, the gene sequence was introduced into a pET28 vector (Novagen). Sanger sequencing (GATC) was conducted to verify the successful insertion of the gene and rule out undesired mutations. An N-terminal hexahistidine sequence was added to the VHPO open reading frame for the cloning process.

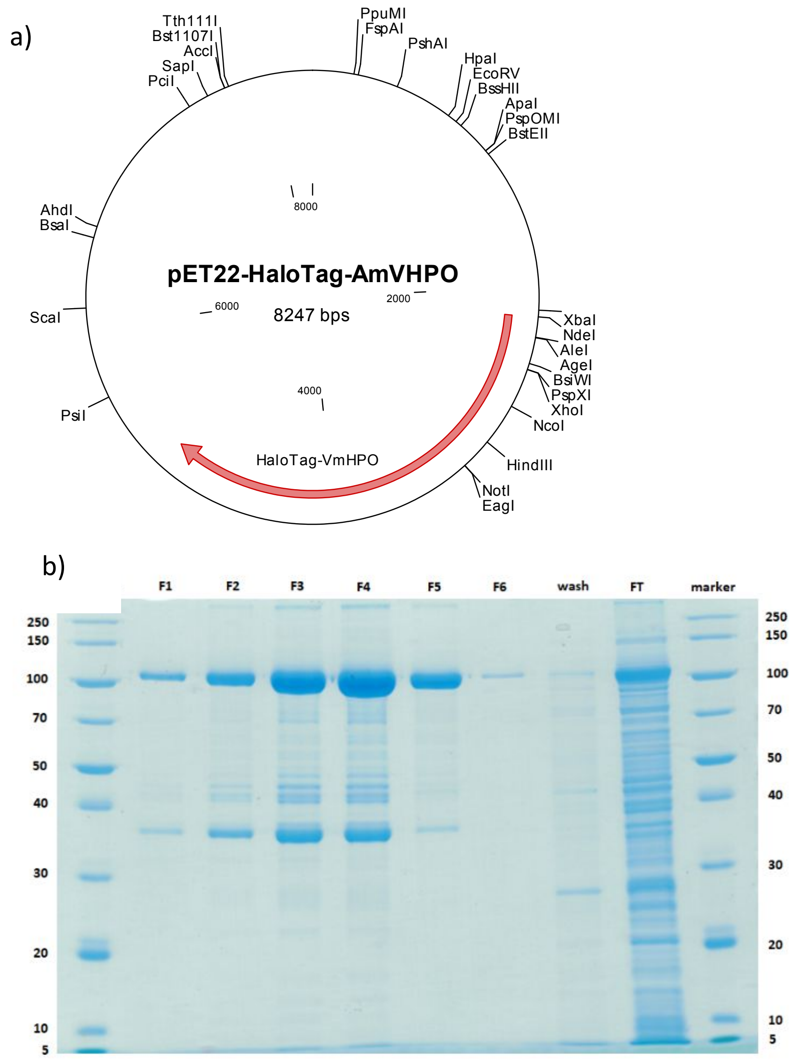

Figure S1. a) Vector map of the pET22::*amvhpoI* construct. b) SDS-Page of the purification of *Am*VHPOI$_{\text{HaloTag}}$; FT = flow through; W = wash; F1 – F6 = elution fractions; the corresponding molecular weights of the protein marker are stated in kilodaltons (kDa); the size of *Am*VHPOI+His+HaloTag approx. 107 kDa.

The pET22HaloTag::amvhpoI expression vector was used to transform E. coli BL21 (DE3) cells for protein production. Recombinant cells were grown on LB agar plates and subsequently used for the inoculation in auto-induction medium, which contained ampicillin as a selection antibiotic (100 µg mL^{-1} final concentration). Cells were grown in 2 L expression cultures at 37°C for 3.5 h while being shaken at 180 rpm. After inoculation, the temperature was lowered to 20°C, at which the cultures were incubated for 48 h. Cells were harvested by centrifugation (5.000 g, 5 min, 4 °C), washed with 0.9% NaCl solution, and then resuspended in lysis buffer (50 mM Tris, pH 7.0, 300 mM NaCl). The suspension was sonicated on ice and centrifuged at 12,000 g at 4 °C for 30 min. Subsequently, the soluble fraction was incubated with Ni-NTA resin (Jena Bioscience, 1 mL per 1 L culture) and gently shaken on ice for 1.5 h.

The resin-containing solution was transferred to a gravity flow column, and the flow through was eluted. After washing the bead bed with 20 mL of binding buffer (50 mM Tris, pH 7.5, 300 mM NaCl, 20 mM imidazole), the His-tagged protein was eluted in fractions (50 mM Tris, pH 7.5, 300 mM NaCl, 250 mM imidazole). Fractions of the highest purity were pooled, and the buffer was exchanged to storage buffer (50 mM Tris pH 7.0, 100 µM Na_3VO_4) using buffer-exchange columns (PD-10 desalting column, GE Healthcare). After concentration (if required), the enzyme was flash-frozen using liquid nitrogen and stored at - 80°C until further use.

Alternatively, *Am*$VHPO_{HaloTag}$ was kept in MES buffer (50 mM, pH 6.0) and 100 µM Na_3VO_4 to maintain the pH of the reactions at pH 6. However, the stability of *Am*$VHPO_{HaloTag}$ in the MES buffer was notably lower. Alternative to this purification method, *Am*$VHPO_{HaloTag}$ could be directly immobilized onto HaloLink resin using the supernatant of the cell lysate (cf. chapter 3.4).

3.2 Sequence of the pET22-HaloTag-*Am*VHPO plasmid

TCGTTAATACAGATGTAGGTGTTCCACAGGGTAGCCAGCAGCATCCTGCGATGCAGATCCGGAACATAATGGTGCAGGGCGCTGACTTCCGCGTTTCCAG
ACTTTACGAAACACGGAAACCGAAGACCATTCATGTTGTTGCTCAGGTCGCAGACGTTTTGCAGCAGCAGTCGCTTCACGTTCGCTCGCGTATCGGTGATTC
ATTCTGCTAACCAGTAAGGCAACCCCGCCAGCCTAGCCGGGTCCTCAACGACAGGAGCACGATCATGCGCACCCGTGGGGCCGCCATGCCGGCGATAATG
GCCTGCTTCTCGCCGAAACGTTTGGTGGCGGGACCAGTGACGAAGGCTTGAGCGAGGGCGTGCAAGATTCCGAATACCGCAAGCGACAGGCCGATCATC
GTCGCGCTCCAGCGAAAGCGGTCCTCGCCGAAAATGACCCAGAGCGCTGCCGGCACCTGTCCTACGAGTTGCATGATAAAGAAGACAGTCATAAGTGCGG
CGACGATAGTCATGCCCCGCGCCCACCGGAAGGAGCTGACTGGGTTGAAGGCTCTCAAGGGCATCGGTCGAGATCCCGGTGCCTAATGAGTGAGCTAACT
TACATTAATTGCGTTGCGCTCACTGCCCGCTTTCCAGTCGGGAAACCTGTCGTGCCAGCTGCATTAATGAATCGGCCAACGCGCGGGGAGAGGCGGTTTGC
GTATTGGGCGCCAGGGTGGTTTTTCTTTTCACCAGTGAGACGGGCAACAGCTGATTGCCCTTCACCGCCTGGCCCTGAGAGAGTTGCAGCAAGCGGTCCAC
GCTGGTTTGCCCCAGCAGGCGAAAATCCTGTTTGATGGTGGTTAACGGCGGGATATAACATGAGCTGTCTTCGGTATCGTCGTATCCCACTACCGAGATAT
CCGCACCAACGCGCAGCCCGGACTCGGTAATGGCGCGCATTGCGCCCAGCGCCATCTGATCGTTGGCAACCAGCATCGCAGTGGGAACGATGCCCTCATT
CAGCATTTGCATGGTTTGTTGAAAACCGGACATGGCACTCCAGTCGCCTTCCCGTTCCGCTATCGGCTGAATTTGATTGCGAGTGAGATATTTATGCCAGCC
AGCCAGACGCAGACGCGCCGAGACAGAACTTAATGGGCCCGCTAACAGCGCGATTTGCTGGTGACCCAATGCGACCAGATGCTCCACGCCCAGTCGCGTA
CCGTCTTCATGGGAGAAAATAATACTGTTGATGGGTGTCTGGTCAGAGACATCAAGAAATAACGCCGGAACATTAGTGCAGGCAGCTTCCACAGCAATGG
CATCCTGGTCATCCAGCGGATAGTTAATGATCAGCCCACTGACGCGTTGCGCGAGAAGATTGTGCACCGCCGCTTTACAGGCTTCGACGCCGCTTCGTTCTA
CCATCGACACCACCACGCTGGCACCCAGTTGATCGGCGCGAGATTTAATCGCCGCGACAATTTGCGACGGCGCGTGCAGGGCCAGACTGGAGGTGGCAAC
GCCAATCAGCAACGACTGTTTGCCCGCCAGTTGTTGTGCCACGCGGTTGGGAATGTAATTCAGCTCCGCCATCGCCGCTTCCACTTTTTCCCGCGTTTTCGCA
GAAACGTGGCTGGCCTGGTTCACCACGCGGGAAACGGTCTGATAAGAGACACCGGCATACTCTGCGACATCGTATAACGTTACTGGTTTCACATTCACCAC
CCTGAATTGACTCTCTTCCGGGCGCTATCATGCCATACCGCGAAAGGTTTTGCGCCATTCGATGGTGTCCGGGATCTCGACGCTCTCCCTTATGCGACTCCT
GCATTAGGAAGCAGCCCAGTAGTAGGTTGAGGCCGTTGAGCACCGCCGCCGCAAGGAATGGTGCATGCAAGGAGATGGCGCCCAACAGTCCCCCGGCCA

CGGGGCCTGCCACCATACCCACGCCGAAACAAGCGCTCATGAGCCCGAAGTGGCGAGCCCGATCTTCCCCATCGGTGATGTCGGCGATATAGGCGCCAGC
AACCGCACCTGTGGCGCCGGTGATGCCGGCCACGATGCGTCCGGCGTAGAGGATCGAGATCTCGATCCCGCGAAATTAATACGACTCACTATAGGGGAAT
TGTGAGCGGATAACAATTCCCCTCTAGAAATAATTTTGTTTAACTTTAAGAAGGAGATATACAT**ATGAAACATCACCATCACCATCACGCAGAAATTGGTA**
CGGGATTTCCGTTTGACCCGCATTATGTGGAGGTTCTGGGTGAACGCATGCACTACGTGGATGTTGGTCCGCGCGATGGCACACCGGTGCTGTTTCTGCA
TGGTAATCCGACCTCCAGCTATGTTTGGCGCAACATTATTCCGCATGTCGCCCCAACGCATCGCTGTATTGCCCCAGATCTCATTGGCATGGGCAAAAGC
GACAAACCGGATTTGGGCTACTTCTTCGACGATCACGTACGGTTTATGGACGCCTTTATCGAGGCTCTGGGACTCGAGGAAGTAGTGCTGGTTATTCATG
ACTGGGGCTCTGCATTAGGCTTTCACTGGGCTAAACGGAACCCAGAACGCGTCAAGGGGATTGCCTTCATGGAGTTCATCCGTCCGATTCCGACCTGGG
ATGAATGGCCCGAATTTGCCCGTGAAACCTTTCAGGCGTTTCGTACCACGGATGTTGGCCGTAAGCTCATCATCGACCAAAACGTGTTCATTGAGGGCAC
TCTTCCCATGGGAGTAGTGCGTCCTTTAACCGAAGTCGAGATGGACCACTATCGCGAACCCTTCCTGAATCCGGTTGATCGCGAACCGCTGTGGCGCTTC
CCGAATGAGCTGCCTATTGCTGGTGAACCGGCGAATATCGTGGCACTTGTGGAAGAATACATGGATTGGCTGCATCAGAGTCCAGTCCCTAAGCTGTTG
TTTTGGGGTACACCTGGCGTGTTGATTCCGCCTGCAGAAGCTGCTCGCTTAGCGAAAAGCTTGCCCAACTGCAAAGCGGTCGATATTGGGCCAGGTCTG
AACCTGTTACAGGAGGATAACCCGGATCTGATCGGGAGTGAAATCGCGCGTTGGCTGTCAACTCTGGAAATCTCGGGTCTTGCAGAAGCAGCGGCCAA
AGAAGCTGCGGCCAAAGAGGCAGCCGCGAAAGAAGCAGCGGCGAAAGCGGCCGCGATGAATACCCGTCGTCAACAGGCTCAGAACATTCGCAACAAT
GCGGCGGAACTGGCAGCCAACCGCCCACATCCTCAGCACATCAACAACAAGGAGGAATACGAATACCGTCGCCCGAAGAAAGATGGCAATGAACCGTC
CCATATTGCAAACTTCACTAAAGGCCTGCCACACGACGAACATACGGGGCTCCTCCTTAATAGCGCAGACTACGACCAATTCGTGTTAGGCATTCAAAGT
GGTGACACAACCGATTTTGCGCGTACTCCGTTAGGACCCGCCGAACTGCCGAAAGTCCATGGTTGCTTGAGTAAGCAGAAGATCGACTGCGATGACGAT
CACCGTAGCGGGTTTTGGAAAAGCCAGATCGCGCAGGGCGCTGCAGGCGGCGATGGCGCGAAATTGCGTGCCTGGGAATCTGCGGGTGCAGGACTGG
TATTTGACCTTGAAGGCCCAGATGCCCAAGCCGTCACCATGCCTCCTGCGCCACGCCTCGAATCGCCGGAACTGACCTCCGAAATCGCCGAAGTGTATTC
CCAAGCGCTGCTCCGGGACATCCACTTTAGCCAGTTACGCGATCCAGGTCTGGGCGATCAGGTTAACGCCTGTGATTCTTGCCCAACGCAACTGAGTATC
TATGAGGCTATCGACATCCTGAACACCGTGCAAATTGAAGGCCAGAACTGGTTTTCGGCGAATTGCTGCGATTTGACAGACGATGAGCAAGCGCGTCA
GCGCCCGCTGGTAACCCGTCAGAACATCTTTCGCGGAATTGCTCCCGGCGACGATGTTGGCCCGTACCTCAGCCAGTTTCTGCTGATTGGCAACAACGCA
CTGGGAGGTGGTGTCTTTGGCCAAGAAGCTGGTCATATTGGGTATGGTGCCATTCGCATTGACCAGCGTGTTCGCAAGGCCACTCCGTGTAAAGACTTC
ATGACCAATTTCGAAACGTGGCTGGATGTCCAGAATGGTGCCGATCTGCGCGGGCTTGAAACCTATGTGGACGCAGATCCCGGCAAATGTCGCGAGTTC
CCGGCATATCGCGTCATTACCACCCCACGGGATTTGGCGACGTATGTACACTACGATGCGCTGTATGAGGCGTATCTGAACGCGTGTCTGATTCTCCTCG
GTATGGGTGCTCCGTTTGATCCTGGGATTCCCTTTCAAAAGCCGGACGTTGAAGATAAACAGCAGGGCTTTGCGCATTTCGGTGGCCCTCAGATTTTGAC
GCTTGTGTGCGAAGCGGCAACTCGCGGTCTGAAAGCAGTACGGTTTCAGAAATTCAATGTGCATCGTCGCTTACGCCCGGAAGCCCTGGGTGGTTTAGT
TGATCGCTACAAACATGGTAAAGGCGCCGGTGATGAGCTGAAACCTGTAGCGGCCCTTGTGGAGGCTCTGGAGAATGTGGGTTTGCTGAGTAAAGTTG
TGGCGCACAACCAATTACAGAACCAGAATTTAGATCGCTCAGGCGATCCGAGCTCTGCTGGCGACAACTACTTCCTGCCCATGGCTTTTCCGGAGGGCTC
ACCGATGCATCCGTCGTATGGTGCAGGCCACGCTACAGTTGCGGGCGCGTGTGTGACGATGCTGAAAGCCTTCTTCGATCATGGATGGCAGCTGAATTT
GGGGATGGCGAATGGCAAATACATTAGCTATGAGCCGAATCAGGACGGCTCCTCACTGCAACAGGTTCTGCTGGATTGCCCGTTAACAGTGGAAGGTG
AACTGAACAAAATCGCAGCAAATATCAGCATTGGGCGTGATTGGGCCGGCGTCCACTATTTTACCGATTACATCGAATCGCTGCGTTTGGGTGAGAAAA
TTGCCATTGGAATTCTTGAAGAACAGAAACTGACCTATGGGGAAAACTTCACGATGACTGTCCCGCTGTATGATGGAGGGTCTATCCAAATTTAAGATC
CGGCTGCTAACAAAGCCCGAAAGGAAGCTGAGTTGGCTGCTGCCACCGCTGAGCAATAACTAGCATAACCCCTTGGGGCCTCTAAACGGGTCTTGAGGGG
TTTTTTGCTGAAAGGAGGAACTATATCCGGATTGGCGAATGGGACGCGCCCTGTAGCGGCGCATTAAGCGCGGCGGGTGTGGTGGTTACGCGCAGCGTG
ACCGCTACACTTGCCAGCGCCCTAGCGCCCGCTCCTTTCGCTTTCTTCCCTTCCTTTCTCGCCACGTTCGCCGGCTTTCCCCGTCAAGCTCTAAATCGGGGGCT
CCCTTTAGGGTTCCGATTTAGTGCTTTACGGCACCTCGACCCCAAAAAACTTGATTAGGGTGATGGTTCACGTAGTGGGCCATCGCCCTGATAGACGGTTTT
TCGCCCTTTGACGTTGGAGTCCACGTTCTTTAATAGTGGACTCTTGTTCCAAACTGGAACAACACTCAACCCTATCTCGGTCTATTCTTTTGATTTATAAGGG
ATTTTGCCGATTTCGGCCTATTGGTTAAAAAATGAGCTGATTTAACAAAAATTTAACGCGAATTTTAACAAAATATTAACGTTTACAATTTCAGGTGGCACTT
TTCGGGGAAATGTGCGCGGAACCCCTATTTGTTTATTTTTCTAAATACATTCAAATATGTATCCGCTCATGAGACAATAACCCTGATAAATGCTTCAATAATA
TTGAAAAAGGAAGAGTATGAGTATTCAACATTTCCGTGTCGCCCTTATTCCCTTTTTTGCGGCATTTTGCCTTCCTGTTTTTGCTCACCCAGAAACGCTGGTG
AAAGTAAAAGATGCTGAAGATCAGTTGGGTGCACGAGTGGGTTACATCGAACTGGATCTCAACAGCGGTAAGATCCTTGAGAGTTTTCGCCCCGAAGAAC
GTTTTCCAATGATGAGCACTTTTAAAGTTCTGCTATGTGGCGCGGTATTATCCCGTATTGACGCCGGGCAAGAGCAACTCGGTCGCCGCATACACTATTCTC
AGAATGACTTGGTTGAGTACTCACCAGTCACAGAAAAGCATCTTACGGATGGCATGACAGTAAGAGAATTATGCAGTGCTGCCATAACCATGAGTGATAA
CACTGCGGCCAACTTACTTCTGACAACGATCGGAGGACCGAAGGAGCTAACCGCTTTTTTGCACAACATGGGGGATCATGTAACTCGCCTTGATCGTTGGG
AACCGGAGCTGAATGAAGCCATACCAAACGACGAGCGTGACACCACGATGCCTGCAGCAATGGCAACAACGTTGCGCAAACTATTAACTGGCGAACTACT
TACTCTAGCTTCCCGGCAACAATTAATAGACTGGATGGAGGCGGATAAAGTTGCAGGACCACTTCTGCGCTCGGCCCTTCCGGCTGGCTGGTTTATTGCTG
ATAAATCTGGAGCCGGTGAGCGTGGGTCTCGCGGTATCATTGCAGCACTGGGGCCAGATGGTAAGCCCTCCCGTATCGTAGTTATCTACACGACGGGGAG
TCAGGCAACTATGGATGAACGAAATAGACAGATCGCTGAGATAGGTGCCTCACTGATTAAGCATTGGTAACTGTCAGACCAAGTTTACTCATATATACTTTA
GATTGATTTAAAACTTCATTTTTAATTTAAAAGGATCTAGGTGAAGATCCTTTTTGATAATCTCATGACCAAAATCCCTTAACGTGAGTTTTCGTTCCACTGAG
CGTCAGACCCCGTAGAAAAGATCAAAGGATCTTCTTGAGATCCTTTTTTTCTGCGCGTAATCTGCTGCTTGCAAACAAAAAAACCACCGCTACCAGCGGTGG
TTTGTTTGCCGGATCAAGAGCTACCAACTCTTTTTCCGAAGGTAACTGGCTTCAGCAGAGCGCAGATACCAAATACTGTCCTTCTAGTGTAGCCGTAGTTAG
GCCACCACTTCAAGAACTCTGTAGCACCGCCTACATACCTCGCTCTGCTAATCCTGTTACCAGTGGCTGCTGCCAGTGGCGATAAGTCGTGTCTTACCGGGT
TGGACTCAAGACGATAGTTACCGGATAAGGCGCAGCGGTCGGGCTGAACGGGGGGTTCGTGCACACAGCCCAGCTTGGAGCGAACGACCTACACCGAAC
TGAGATACCTACAGCGTGAGCTATGAGAAAGCGCCACGCTTCCCGAAGGGAGAAAGGCGGACAGGTATCCGGTAAGCGGCAGGGTCGGAACAGGAGAG
CGCACGAGGGAGCTTCCAGGGGGAAACGCCTGGTATCTTTATAGTCCTGTCGGGTTTCGCCACCTCTGACTTGAGCGTCGATTTTTGTGATGCTCGTCAGG
GGGGCGGAGCCTATGGAAAAACGCCAGCAACGCGGCCTTTTTACGGTTCCTGGCCTTTTGCTGGCCTTTTGCTCACATGTTCTTTCCTGCGTTATCCCCTGAT
TCTGTGGATAACCGTATTACCGCCTTTGAGTGAGCTGATACCGCTCGCCGCAGCCGAACGACCGAGCGCAGCGAGTCAGTGAGCGAGGAAGCGGAAGAG
CGCCTGATGCGGTATTTTCTCCTTACGCATCTGTGCGGTATTTCACACCGCATATATGGTGCACTCTCAGTACAATCTGCTCTGATGCCGCATAGTTAAGCCA
GTATACACTCCGCTATCGCTACGTGACTGGGTCATGGCTGCGCCCCGACACCCGCCAACACCCGCTGACGCGCCCTGACGGGCTTGTCTGCTCCCGGCATC
CGCTTACAGACAAGCTGTGACCGTCTCCGGGAGCTGCATGTGTCAGAGGTTTTCACCGTCATCACCGAAACGCGCGAGGCAGCTGCGGTAAAGCTCATCA
GCGTGGTCGTGAAGCGATTCACAGATGTCTGCCTGTTCATCCGCGTCCAGCTCGTTGAGTTTCTCCAGAAGCGTTAATGTCTGGCTTCTGATAAAGCGGGC
CATGTTAAGGGCGGTTTTTTCCTGTTTGGTCACTGATGCCTCCGTGTAAGGGGGATTTCTGTTCATGGGGGTAATGATACCGATGAAACGAGAGAGGATGC

TCACGATACGGGTTACTGATGATGAACATGCCCGGTTACTGGAACGTTGTGAGGGTAAACAACTGGCGGTATGGATGCGGCGGGACCAGAGAAAAATCA
CTCAGGGTCAATGCCAGCGCT

3.3 Determination of the *Am*VHPO$_{wild\text{-}type}$ vs *Am*VHPO$_{HaloTag}$ Activity using MCD Activity Assay[1]

A stock solution of MCD (1 mM) was prepared in 2 M aqueous NaOAc. A master mix containing MCD (50 µM), KBr (200 mM), the respective *Am*VHPO (1 µL of purified batch or dilution thereof), and Na_3VO_4 (1 µM) in MES buffer (50 mM, pH 6.0) was prepared.

For assay initiation, 30 µL H_2O_2 (10 mM final concentration) was added to 270 µL of the master mix, bringing the total volume to 300 µL. The mixture was transferred onto a 96-well plate, and the decrease in absorbance at 290 nm was monitored at 30 °C.

The concentration of converted MCD was calculated from A290 using the equation:

$$c(MCD)[mM] = \frac{A_{290}}{(\mathcal{E}_{MCD} * d)}$$

with $\mathcal{E}_{MCD} = 19.9\ \mathrm{mM^{-1}cm^{-1}}$ and $\mathrm{d} = 0.91\ \mathrm{cm}$

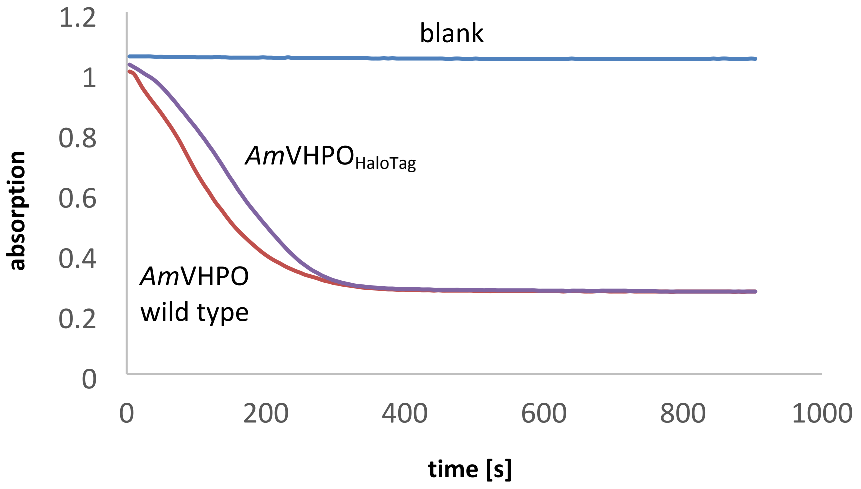

Figure S2. MCD assay as proof for *ex vivo* halogenation activity of *Am*VHPOI$_{HaloTag}$. HaloTag and HisTag *Am*VHPO show comparable bromination activity.

3.4 Immobilization of *Am*VHPO$_{HaloTag}$ on HaloLink Resin[2]

Both purified enzyme and lyophilized cell lysate were utilized for the immobilization of the *Am*VHPO$_{HaloTag}$ onto the HaloLink resin. To prepare lyophilized cell lysate, the cell pellet was resuspended in lysis buffer (10 mM imidazole, 50 mM NaH_2PO_4, 300 mM NaCl). After sonication and centrifugation, the supernatant was gathered and freeze-dried.

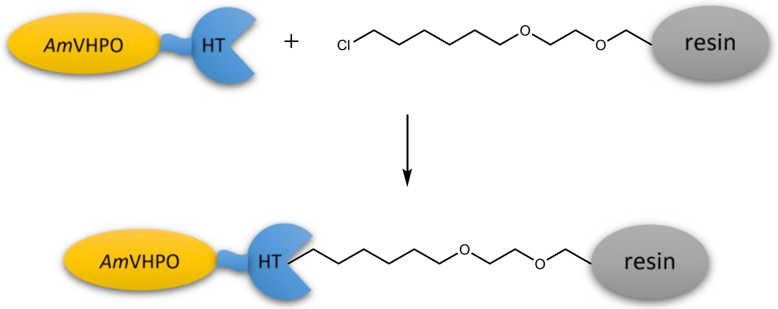

Scheme S1. Modified *Am*VHPO$_{HaloTag}$ forms a covalent bond with the designed ligand on HaloLink resin; HT = HaloTag; resin = HaloLink resin.

The solution of HaloLink resin, stored in 20 vol% ethanol, was exchanged with Tris buffer solution (50 mM, pH 7.0, 100 µM Na_3VO_4, 0.1 vol% Triton X-100). *Am*VHPO$_{HaloTag}$ (cf. chapter 3.1) was incubated with the washed resin at 25 °C and 1000 rpm for 5 h. The resin was then washed with the aforementioned buffer solution to remove the unbound enzyme.

3.5 Bicinchoninic acid (BCA) Assay for the Determination of Enzyme Loading[3]

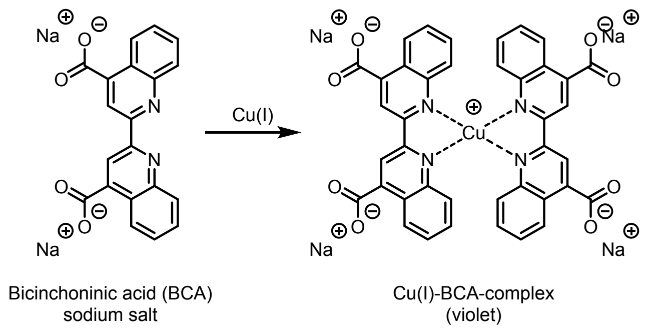

Scheme S2. Underlying principle of the BCA assay.

The BCA assay is a colorimetric method to quantify enzyme concentration. In the presence of peptide bonds in alkaline medium, Cu^{2+} is reduced to Cu^{+}. The bicinchoninic acid chelates Cu^{+} ions with high specificity to form a water-soluble, purple-colored complex (λ_{max} = 562 nm).

A commercially available BCA Protein Quantitation Kit from interchim uptima was used for the assay, containing solution A (containing bicinchoninic acid) and solution B (containing Cu^{2+} ions). For the assay, 50 parts of solution A and 1 part of solution B were mixed. 25 µL enzyme solution was added to 200 µL BCA solution. The resulting mixture was shaken at 37 °C and 1000 rpm for 30 min. After centrifugation, 150 µL of the supernatant was transferred onto a 96-well plate, and the absorption at 562 nm was measured. The enzyme concentration was determined using linear regression.

A calibration curve was established for quantification of the enzyme loading by conducting the assay described above with a dilution series of defined concentrations (2 mg ml^{-1} to 20 µg ml^{-1}) with a BSA standard in the same buffer.

4. Procedures

4.1 General Procedure (GP-1) for the Enzymatic Synthesis of Brominated Indoles

A solution containing substrate **4** (7.50 mM; 1.20 µmol), KBr (1.1 eq, 8.25 mM), Na_3VO_4 (190 µM) and *Am*VHPO$_{HaloTag}$ (5 µL; 3 mg mL^{-1}; 6 U; preincubated with 30 µL of 30 mM K_3VO_4) in MES buffer (pH 6.0, 50 mM) and MeCN or THF (50:50, v/v, 7.5 mM) was prepared and H_2O_2 (1.1 eq, 8.25 mM) was added to initiate the reaction (total volume: 160 µL). The reaction mixture was shaken at 30°C and 1200 rpm for 24 h. The reaction was terminated by the addition of 100 µL of saturated $(NH_4)_2SO_4$ and NaCl, respectively.

4.2 General Procedure (GP-2) for the Synthesis of One-Pot Reactions Combining Enzymatic Bromination and Suzuki Cross-Coupling Reaction

A solution containing substrate **4** (7.50 mM, 12.0 µmol), KBr (1.1 eq, 8.25 mM), Na_3VO_4 (190 µM), and *Am*VHPO$_{immo}$ (50 µL; 3 mg mL^{-1}; 6 U; preincubated with 300 µL of 30 mM K_3VO_4) in MES buffer (pH 6.0, 50 mM) and MeCN (50:50, v/v, 7.5 mM) was prepared and H_2O_2 (1.1 eq, 8.25 mM) was added to initiate the reaction (total volume: 1.6 mL). The reaction mixture was shaken at 30°C and 1200 rpm for 24 h. MnO_2 (1.20 µmol, 0.1 eq) was added, and the mixture was shaken for 1 h at 30°C. The resulting suspension was centrifugated (5 min, 7000 g, 4°C), and the supernatant was transferred into a Schlenk tube. Boronic acid (14.4 µmol, 1.2 eq), TPPTS (0.72 µmol, 0.06 eq), NEt_3 (0.24 mmol, 20 eq), and $Pd(OAc)_2$ (0.36 µmol, 0.03 eq) were added to the degassed solution. The mixture was stirred at 50°C for 20 h, after which it was cooled to room temperature. The pH of the reaction mixture was adjusted to 6 using 2M HCl, and the aqueous phase was extracted with EtOAc (3x 2mL). The combined organic phases were washed with brine, dried over Na_2SO_4, filtered, and concentrated under reduced pressure. The crude product was purified on SiO_2.

4.3 Synthesis of Product 6a on a mmol Scale

A solution containing 227 mg indole **4a** (7.50 mM, 1.20 mmol), KBr (157 mg, 1.32 mmol, 1.1 eq, 8.25 mM), Na_3VO_4 (304 µmol, 190 µM), and *Am*VHPO$_{immo}$ (5 mL; 3 mg mL^{-1}; 6 U; preincubated with 30 mL of 30 mM K_3VO_4) in MES buffer (pH 6.0, 50 mM) and MeCN (50:50, v/v, 7.5 mM) was prepared and H_2O_2 (1.32 mmol, 1.1 eq, 8.25 mM) was added to initiate the reaction (total volume: 160 mL). The reaction mixture was shaken at 30°C at a heatable shaker and 1200 rpm for 24 h. MnO_2 (10.4 mg, 120 µmol, 0.1 eq) was added, and the mixture was shaken for 1 h at 30°C. The resulting suspension was

centrifugated (5 min, 7000 g, 4°C), and the supernatant was transferred into a Schlenk tube. Boronic acid (176 mg, 1.44 mmol, 1.2 eq), TPPTS (40.9 mg, 72.0 µmol, 0.06 eq), NEt_3 (2.24g, 24.0 mmol, 20 eq), and $Pd(OAc)_2$ (225 mg, 36.0 µmol, 0.03 eq) were added to the degassed solution. The mixture was stirred for 20 h at 50°C using a water bath, after which it was cooled to room temperature. The pH of the reaction mixture was adjusted to 6 using 2M HCl, and the aqueous phase was extracted with EtOAc (3x 200 mL). The combined organic phases were washed with brine, dried over Na_2SO_4, filtered, and concentrated under reduced pressure. The crude product was purified on SiO_2 using pentane:EtOAc (97:3) as eluent. 315 mg (1.19 mmol, 99%) of product **6a** was obtained as a colorless oil.

5. Physical and Spectroscopic Data

5.1 Physical and Spectroscopic Data of Starting Material and Brominated Intermediate

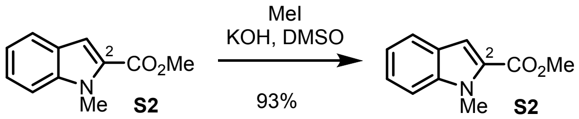

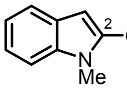
S2

Methyl 1-methyl-1*H*-indole-2-carboxylate (**S2**): Anhydrous DMSO (20 mL, 0.5 M) was added to KOH (2.24 g, 40.0 mmol, 4.0 eq, crushed pellets), and the mixture was stirred at room temperature for 5 min. Methyl 1*H*-indole-2-carboxylate (1.75 g, 10.0 mmol, 1 eq) was added to the mixture and stirred at room temperature for 1 h. MeI (1.3 mL, 2.84 g, 20.0 mmol, 2.0 eq) was added dropwise, and the mixture was stirred at room temperature. Upon the completion of the reaction (monitored *via* TLC), water was added and the aqueous phase was extracted with diethyl ether. The organic phase was washed with water and dried over Na_2SO_4. After filtration, the solvent was removed in vacuo to give the desired product as a colorless solid (1.76 g, 9.30 mmol, 93%); **R_f** = 0.6 (silica gel, pentane/EtOAc 97:3); **^{1}H NMR** (300 MHz, $CDCl_3$) δ = 7.68 (dt, *J* = 8.0, 1.0 Hz, 1H), 7.42 – 7.34 (m, 2H), 7.30 (d, *J* = 0.8 Hz, 1H), 7.15 (ddd, *J* = 8.0, 6.3, 1.7 Hz, 1H), 4.09 (s, 3H), 3.92 (s, 3H) ppm; **^{13}C NMR** (75 MHz, $CDCl_3$) δ = 162.7, 139.7, 127.6, 125.9, 125.0, 122.6, 120.6, 110.3, 51.62, 31.60 ppm; **MS** (EI, 70 eV): *m/z* (%) = 189 (100) $[M]^+$, 174 (20) $[M-CH_3]^+$, 158 (45) $[M-OCH_3]^+$.

The spectroscopic data is in accordance to that reported in the literature.[5]

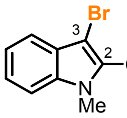
4a

Methyl 3-bromo-1-methyl-1*H*-indole-2-carboxylate (**4a**): prepared from indole **S2** (0.23 mg, 1.20 µmol) applying **GP-1**; colorless solid (0.32 mg, 1.2 µmol, quant.); **R_f** = 0.6 (silica gel, pentane/EtOAc 97:3); **^{1}H NMR** (300 MHz, $CDCl_3$) δ = 7.68 (dt, *J* = 8.1, 1.0 Hz, 1H), 7.44 – 7.36 (m, 2H), 7.25 – 7.20 (m, 1H), 4.05 (s, 3H), 3.99 (s, 3H) ppm; **^{13}C NMR** (75 MHz, $CDCl_3$) δ = 162.1, 138.4, 126.8, 126.3, 126.2, 121.7, 121.4, 110.5, 98.87, 51.98, 32.82 ppm; **MS** (EI, 70 eV): *m/z* (%) = 268 (100) $[M]^+$, 252 (8) $[M-CH_3]^+$, 236 (29) $[M-OCH_3]^+$, 208 (8) $[M-CO_2CH_3]^+$, 194 (2) $[M-CH_3-CO_2CH_3]^+$.

The spectroscopic data is in accordance with that reported in the literature.[4]

5.2 Physical and Spectroscopic Data of the Products 6

Methyl 1-methyl 3-phenyl-1*H*-indole-2-carboxylate (**6a**): prepared from indole **4a** (2.27 mg, 12.0 µmol) and phenylboronic acid (1.76 mg, 14.4 µmol) in MeCN applying **GP-2**; colorless oil (3.18 mg, 12.0 µmol, quant.); **R**$_f$ = 0.38 (silica gel, pentane/EtOAc 97:3); **^{1}H NMR** (400 MHz, $CDCl_3$) δ = 7.58 (dd, *J* = 8.1, 1.1 Hz, 1H), 7.49 – 7.31 (m, 7H), 7.15 (virt. tt, $J_{virt.}$ = 6.6, 1.0 Hz, 1H), 4.08 (s, 3H), 3.71 (s, 3H) ppm; **^{13}C NMR** (101 MHz, $CDCl_3$) δ = 163.3, 138.7, 134.8, 130.5, 128.0, 127.0, 126.7, 125.5, 124.8, 124.7, 121.8, 120.8, 110.2, 51.51, 32.13 ppm; **MS** (EI, 70 eV): *m/z* (%) = 265 (100) $[M]^+$, 250 (3) $[M-CH_3]^+$, 234 (25) $[M-CO_2CH_3]^+$, 206 (7) $[M-CO_2CH_3]^+$, 191 (9) $[M-CH_3-CO_2CH_3]^+$.

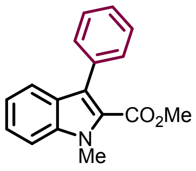

6a, quant

The spectroscopic data is in accordance with that reported in the literature.[6]

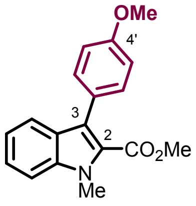

6b, quant.

Methyl 3-(4'-methoxyphenyl)-1-methyl-1*H*-indole-2-carboxylate (**6b**): prepared from indole **4a** (2.27 mg, 12.0 µmol) and (4-methoxyphenyl)boronic acid (2.19 mg, 14.4 µmol) in MeCN applying **GP-2**; colorless solid (3.54 mg, 12.0 µmol, quant.); **m.p.** = 103 °C (EtOAc); **R**$_f$ = 0.5 (silica gel, pentane/EtOAc 97:3); **^{1}H NMR** (400 MHz, $CDCl_3$) δ = 7.58 (dt, *J* = 8.2, 1.0 Hz, 1H) 7.35 – 7.45 (m, 4H), 7.14 (ddd, *J* = 8.0, 6.3, 1.5 Hz, 1H), 6.97 – 7.03 (m, 2H), 4.07 (s, 3H), 3.89 (s, 3H), 3.73 (s, 3H) ppm; **^{13}C NMR** (101 MHz, $CDCl_3$) δ = 163.4, 158.8, 138.7, 131.6, 127.0, 126.9, 125.5, 124.7, 124.5, 121.8, 120.7, 113.5, 110.2, 55.41, 51.51, 32.17 ppm; **IR** (film) $\tilde{\upsilon}_{max}$ = 2951, 2835, 1702, 1258, 1242, 1196, 1177, 749 cm^{-1}; **MS** (EI, 70 eV): *m/z* (%) = 295 (100) $[M]^+$, 280 (38) $[M-CH_3]^+$, 264 (4) $[M-OCH_3]^+$, 220 (4) $[M-CO_2CH_3-CH_3-H]^+$; **HRMS** (ESI) calcd. for $C_{18}H_{18}NO_3^+$ $[M+H]^+$ 296.1281, found 296.1281.

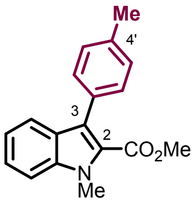

6c, 97%

Methyl 1-methyl-3-(4'-tolyl)-1*H*-indole-2-carboxylate (**6c**): prepared from indole **4a** (2.27 mg, 12.0 µmol) and *p*-tolylboronic acid (2.19 mg, 14.4 µmol) in MeCN applying **GP-2**; colorless solid (3.25 mg, 11.6 µmol, 97%); **m.p.** = 83 °C (EtOAC); **R**$_f$ = 0.38 (silica gel, pentane/EtOAc 97:3); **^{1}H NMR** (400 MHz, $CDCl_3$) δ = 7.59 (dt, *J* = 8.1, 1.0 Hz, 1H), 7.45 – 7.24 (m, 6H), 7.14 (ddd, *J* = 8.0, 6.4, 1.5 Hz, 1H), 4.07 (s, 3H), 3.73 (s, 3H), 2.44 (s, 3H) ppm; **^{13}C NMR** (101 MHz, $CDCl_3$) δ = 163.4, 138.7, 136.6, 131.7, 130.3, 128.7, 126.8, 125.4, 124.8, 124.7, 121.8, 120.7, 110.2, 51.52, 32.15, 21.45 ppm; **IR** (film) $\tilde{\upsilon}_{max}$ = 2955, 2921, 2854, 1699, 1435, 1259, 1245, 1196, 1152, 1102, 1089, 820, 747 cm^{-1}; **MS** (EI, 70 eV): *m/z* (%) = 279 (100) $[M]^+$, 248 (13) $[M-OCH_3]^+$, 220 (6) $[M-CO_2CH_3]^+$, 204 (13) $[M-CO_2CH_3-CH_3-H]^+$; **HRMS** (EI) calcd. for $C_{18}H_{17}NO_2$ [M] 279.1254, found 279.1250.

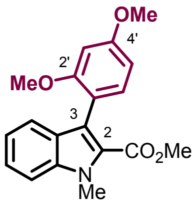

6d, 91%

Methyl 3-(2',4'-dimethoxyphenyl)-1-methyl-1*H*-indole-2-carboxylate (6d): prepared from indole **4a** (2.27 mg, 12.0 µmol) and (2,4-dimethoxyphenyl)boronic acid (2.62 mg, 14.4 µmol) in MeCN applying **GP-2**; off-white solid (3.55 mg, 10.91 µmol, 91%); **m.p.** = 107 °C (EtOAc); $\mathbf{R_f}$ = 0.36 (silica gel, pentane/EtOAc 91:9); **^{1}H NMR** (400 MHz, $CDCl_3$) δ = 7.52 (dt, *J* = 8.2, 1.0 Hz, 1H), 7.33 – 7.44 (m, 2H), 7.30 (d, *J* = 8.2 Hz, 1H), 7.12 (ddd, *J* = 8.0, 6.6, 1.2 Hz, 1H), 6.56 – 6.64 (m, 2H), 4.06 (s, 3H), 3.89 (s, 3H), 3.72 (s, 3H), 3.70 (s, 3H) ppm; **^{13}C NMR** (101 MHz, $CDCl_3$) δ = 163.6, 160.2, 158.2, 138.7, 131.9, 126.8, 126.0, 125.0, 121.8, 120.5, 119.3, 116.6, 110.2, 104.1, 98.88, 55.60, 55.52, 51.48, 31.97 ppm; **IR** (film) $\tilde{\upsilon}_{max}$ = 3005, 2923, 2853, 1714, 1243, 1208, 1159, 1040, 744 cm^{-1}; **MS** (EI, 70 eV): *m/z* (%) = 325 (100) $[M]^+$, 310 (4) $[M-CH_3]^+$, 294 (4) $[M-OCH_3]^+$, 279 (8) $[M-OCH_3-CH_3]^+$, 266 (17) $[M-CO_2CH_3]^+$, 251 (17) $[M-CO_2CH_3-CH_3]^+$, 236 (17) $[M-CO_2CH_3-2CH_3]^+$; **HRMS** (ESI) calcd. for $C_{19}H_{20}NO_4^+$ $[M+H]^+$ 326.1387, found 326.1388.

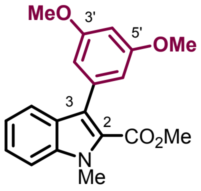

6e,97%

Methyl 3-(3',5'-dimethoxyphenyl)-1-methyl-1*H*-indole-2-carboxylate (6e): prepared from indole **4a** (2.27 mg, 12.0 µmol) and (3,5-dimethoxyphenyl)boronic acid (2.62 mg, 14.4 µmol) in MeCN applying **GP-2**; colorless solid (3.78 mg, 11.6 µmol, 97%); **m.p.** = 107 °C (EtOAc); $\mathbf{R_f}$ = 0.33 (silica gel, pentane/EtOAc 91:9); **^{1}H NMR** (400 MHz, $CDCl_3$) δ = 7.63 (dt, *J* = 8.2, 1.0 Hz, 1H), 7.34 – 7.46 (m, 2H), 7.15 (ddd, *J* = 8.0, 6.4, 1.5 Hz, 1H), 6.60 (d, *J* = 2.3 Hz, 2H), 6.50 (virt. t, $J_{virt.}$ = 2.3 Hz, 1H), 4.06 (s, 3H), 3.82 (s, 6H), 3.74 (s, 3H) ppm; **^{13}C NMR** (101 MHz, $CDCl_3$) δ = 163.3, 160.4, 138.5, 136.7, 126.6, 125.5, 124.9, 124.3, 121.8, 120.9, 110.2, 108.6, 99.52, 55.52, 51.67, 32.09 ppm; **IR** (film) $\tilde{\upsilon}_{max}$ = 2952, 2924, 1700, 1247, 752, 742, 727 cm^{-1}; **MS** (EI, 70 eV): *m/z* (%) = 325 (100) $[M]^+$, 294 (6) $[M-OCH_3]^+$, 267 (6) $[M-H-CO_2CH_3]^+$, 250 (6) $[M-H-CO_2CH_3-CH_3]^+$, 236 (4) $[M-CO_2CH_3-2CH_3]^+$; **HRMS** (ESI) calcd. for $C_{19}H_{20}NO_4^+$ $[M+H]^+$ 326.1387, found 326.1388.

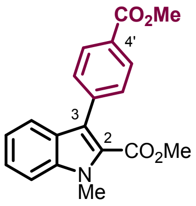

6f, 94%

Methyl 3-(4'-(methoxycarbonyl)phenyl)-1-methyl-1*H*-indole-2-carboxylate (6f): prepared from indole **4a** (2.27 mg, 12.0 µmol) and (4-(methoxycarbonyl)phenyl)boronic acid (2.59 mg, 14.4 µmol) in MeCN applying **GP-2**; yellow oil (3.65 mg, 11.3 µmol, 94%); $\mathbf{R_f}$ = 0.36 (silica gel, pentane/EtOAc 91:9); **^{1}H NMR** (400 MHz, $CDCl_3$) δ = 8.08 – 8.16 (m, 2H), 7.54 (dt, *J* = 8.2, 1.0 Hz, 1H), 7.48 – 7.53 (m, 2H), 7.37 – 7.46 (m, 2H), 7.17 (ddd, *J* = 8.0, 6.5, 1.4 Hz, 1H), 4.09 (s, 3H), 3.96 (s, 3H), 3.70 (s, 3H) ppm; **^{13}C NMR** (101 MHz, $CDCl_3$) δ = 167.3, 163.0, 140.0, 138.7, 130.5, 129.3, 128.7, 126.4, 125.7, 125.0, 123.4, 121.4, 121.3, 110.4, 52.26, 51.61, 32.20 ppm; **IR** (film) $\tilde{\upsilon}_{max}$ = 2950, 2853, 1704, 1608, 1436, 1273, 1259, 1243, 1196, 1177, 1153, 1102, 1088, 740, 709 cm^{-1}; **MS** (EI, 70 eV): *m/z* (%) = 323

(100) $[M]^+$, 292 (17) $[M-OCH_3]^+$, 233 (1) $[M-CO_2CH_3-OCH_3]^+$, 205 (1) $[M-2CO_2CH_3]^+$; **HRMS** (EI) calcd. for $C_{19}H_{17}NO_4$ [M] 323.1152, found 323.1156.

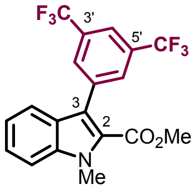

6g,84%

Methyl 3-(3′,5′-bis(trifluoromethyl)phenyl)-1-methyl-1*H*-indole-2-carboxylate (**6g**): prepared from indole **4a** (2.27 mg, 12.0 µmol) and (3,5-bis(trifluoromethyl)phenyl)boronic acid (3.71 mg, 14.4 µmol) in MeCN applying **GP-2**; colorless solid (4.05 mg, 10.1 µmol, 84%); **m.p.** = 98 °C (EtOAc); $\mathbf{R_f}$ = 0.46 (silica gel, pentane/EtOAc 97:3); **^{1}H NMR** (400 MHz, $CDCl_3$) δ = 7.91 – 7.96 (m, 2H), 7.87 – 7.91 (m, 1H), 7.42 – 7.55 (m, 3H), 7.22 (ddd, *J* = 8.1, 6.4, 1.5 Hz, 1H), 4.13 (s, 3H), 3.72 (s, 3H) ppm; **^{13}C NMR** (101 MHz, $CDCl_3$) δ = 162.5, 138.7, 137.1, 131.2 (q, *J* = 33.1 Hz), 130.9 (q, *J* = 2.7 Hz), 126.1, 126.0, 125.1, 123.7 (q, *J* = 272.6 Hz), 121.9, 121.2, 120.7, 120.6 (sept, *J* = 4.0 Hz), 110.7, 51.62, 32.36 ppm; **^{19}F NMR** (376 MHz, $CDCl_3$) δ = -62.76 (s) ppm; **IR** (film) $\tilde{\upsilon}_{max}$ = 2959, 2925, 2854, 1704, 1269, 1176, 1165, 1117, 1107, 895, 742, 708, 683 cm^{-1}; **MS** (EI, 70 eV): *m/z* (%) = 401 (100) $[M]^+$, 386 (9) $[M-CH_3]^+$, 370 (17) $[M-OCH_3]^+$, 343 (9) $[M-H-CO_2CH_3]^+$, 301 (13) $[M-OCH_3-CF_3]^+$, 273 (6) $[M-CO_2CH_3-CF_3]^+$, 258 (4) $[M-CO_2CH_3-CH_3-CF_3]^+$; **HRMS** (EI) calcd. for $C_{19}H_{13}F_6NO_2$ [M] 401.0845, found 401.0837.

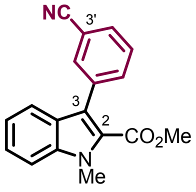

6h,92%

Methyl 3-(3′-cyanophenyl)-1-methyl-1*H*-indole-2-carboxylate (**6h**): prepared from indole **4a** (2.27 mg, 12.0 µmol) and (3-cyanophenyl)boronic acid (2.12 mg, 14.4 µmol) in MeCN applying **GP-2**; colorless solid (3.20 mg, 11.0 µmol, 92%); **m.p.** = 77 °C (EtOAc); $\mathbf{R_f}$ = 0.28 (silica gel, pentane/EtOAc 91:9); **^{1}H NMR** (400 MHz, $CDCl_3$) δ = 7.72 – 7.77 (m, 1H), 7.63 – 7.71 (m, 2H), 7.51 – 7.59 (m, 1H), 7.39 – 7.51 (m, 3H), 7.19 (ddd, *J* = 8.0, 6.4, 1.5 Hz, 1H), 4.10 (s, 3H), 3.72 (s, 3H) ppm; **^{13}C NMR** (101 MHz, $CDCl_3$) δ = 162.6, 138.6, 136.4, 135.1, 134.1, 130.6, 128.8, 126.3, 125.9, 125.0, 122.1, 121.5, 121.0, 119.1, 112.2, 110.6, 51.70, 32.34 ppm; **IR** (film) $\tilde{\upsilon}_{max}$ = 2923, 2853, 2227, 1696, 1264, 1243, 1211, 809, 737, 703 cm^{-1}; **MS** (EI, 70 eV): *m/z* (%) = 290 (100) $[M]^+$, 275 (4) $[M-CH_3]^+$, 259 (19) $[M-OCH_3]^+$, 245 (4) $[M-H-OCH_3-CH_3]^+$, 231 (6) $[M-CO_2CH_3]^+$, 216 (9) $[M-CO_2CH_3-CH_3]^+$, 190 (17) $[M-CO_2CH_3-CH_3-CN]^+$; **HRMS** (ESI) calcd. for $C_{18}H_{15}N_2O_2^+$ $[M+H]^+$ 291.1128, found 291.1129.

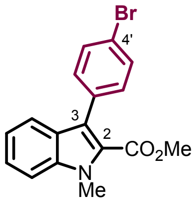

6i, 73% (88%)

Methyl 3-(4′-bromophenyl)-1-methyl-1*H*-indole-2-carboxylate (**6i**): prepared from indole **4a** (2.27 mg, 12.0 µmol) and (4-bromophenyl)boronic acid (2.89 mg, 14.4 µmol) in MeCN applying **GP-2**; colorless oil (3.02 mg, 8.76 µmol, 73%; 88% based on recovered **5a**); $\mathbf{R_f}$ = 0.39 (silica gel, pentane/EtOAc 97:3); **^{1}H NMR** (500 MHz, $CDCl_3$) δ = 7.59 – 7.54 (m, 2H), 7.52 (dt, *J* = 8.1, 1.0 Hz, 1H), 7.45 – 7.38 (m, 2H), 7.32 – 7.28

(m, 2H), 7.15 (ddd, J = 8.0, 6.6, 1.3 Hz, 1H), 4.08 (s, 3H), 3.72 (s, 3H) ppm; **^{13}C NMR** (126 MHz, $CDCl_3$) δ = 163.1, 138.6, 133.8, 132.1, 131.1, 126.4, 125.7, 124.7, 123.4, 121.4, 121.1, 121.1, 110.4, 51.64, 32.24 ppm; **IR** (film) $\tilde{\upsilon}_{max}$ = 2924, 2853, 1704, 1259, 1244, 1199, 1106, 1089, 1011, 740 cm^{-1}; **MS** (EI, 70 eV): m/z (%) = 344 (100) $[M]^+$, 233 (33) $[M\text{-}OCH_3\text{-}Br]^+$, 204 (22) $[M\text{-}CO_2CH_3\text{-}H\text{-}Br]^+$, 190 (13) $[M\text{-}CO_2CH_3\text{-}CH_3\text{-}Br]^+$, 163 (9), 132 (7); **HRMS** (ESI) calcd. for $C_{17}H_{15}BrNO_2^+$ $[M+H]^+$ 344.0281, found 344.0282.

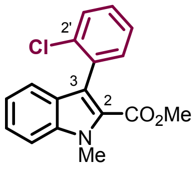

6j, 58%

Methyl 3-(2'-chlorophenyl)-1-methyl-1*H*-indole-2-carboxylate (**6j**): prepared from indole **4a** (2.27 mg, 12.0 μmol) and (2-chlorophenyl)boronic acid (2.25 mg, 14.4 μmol) in MeCN applying **GP-2**; colorless oil (2.09 mg, 6.97 μmol, 58%); **R_f** = 0.44 (silica gel, pentane/EtOAc 97:3); **^{1}H NMR** (400 MHz, $CDCl_3$) δ = 7.48 – 7.54 (m, 1H), 7.29 – 7.47 (m, 6H), 7.14 (ddd, J = 7.8, 6.9, 1.1 Hz, 1H), 4.12 (s, 3H), 3.67 (s, 3H) ppm; **^{13}C NMR** (101 MHz, $CDCl_3$) δ = 163.0), 138.6, 134.6, 134.2, 132.3, 129.5, 128.5, 126.5, 126.4 , 125.7, 125.5), 121.6, 121.3, 121.0, 110.4, 51.69, 32.19 ppm; **IR** (film) $\tilde{\upsilon}_{max}$ = 3058, 2949, 2923, 2853, 1706, 1467, 1438, 1262, 1245, 1199, 1099, 739, 730, 706 cm^{-1}; **MS** (EI, 70 eV): m/z (%) = 299 (43) $[M]^+$, 264 (100) $[M\text{-}Cl]^+$, 249 (85) $[M\text{-}Cl\text{-}CH_3]^+$, 233 (13) $[M\text{-}Cl\text{-}OCH_3]^+$, 218 (4) $[M\text{-}Cl\text{-}OCH_3\text{-}CH_3]^+$, 204 (9) $[M\text{-}Cl\text{-}CO_2CH_3\text{-}H]^+$, 190 (11) $[M\text{-}Cl\text{-}CO_2CH_3\text{-}CH_3]^+$; **HRMS** (ESI) calcd. for $C_{17}H_{15}ClNO_2^+$ $[M+H]^+$ 300.0786, found 300.0786.

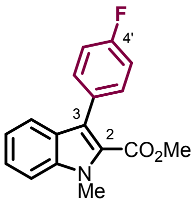

6k, quant.

Methyl 3-(4'-fluorophenyl)-1-methyl-1*H*-indole-2-carboxylate (**6k**): prepared from indole **4a** (2.27 mg, 12.0 μmol) and (4-fluorophenyl)boronic acid (2.01 mg, 14.4 μmol) in MeCN applying **GP-2**; colorless solid (3.40 mg, 12.0 μmol, quant.); **m.p.** = 74 °C (EtOAc); **R_f** = 0.35 (silica gel, pentane/EtOAc 97:3); **^{1}H NMR** (400 MHz, $CDCl_3$) δ = 7.52 (dt, J = 8.0, 0.9 Hz, 1H), 7.36 – 7.45 (m, 4H), 7.09 – 7.18 (m, 3H), 4.08 (s, 3H), 3.71 (s, 3H) ppm; **^{13}C NMR** (101 MHz, $CDCl_3$) δ = 163.1, 138.6, 132.1, 132.0, 130.7, 130.7, 126.8, 125.6, 124.8, 123.7, 121.5, 121.0, 115.0, 114.8, 110.3, 51.54, 32.22 ppm; **^{19}F NMR** (376 MHz, $CDCl_3$) δ = -115.83 (dddd, J = 8.8, 5.5 Hz) ppm; **IR** (film) $\tilde{\upsilon}_{max}$ = 3067, 2946, 2923, 2852, 1696, 1535, 1497, 1442, 1371, 1260, 1244, 1224, 1196, 1155, 1135, 1108, 1099, 1088, 835, 736, 721 cm^{-1}; **MS** (EI, 70 eV): m/z (%) = 283 (100) $[M]^+$, 252 (21) $[M\text{-}OCH_3]^+$, 224 (4) $[M\text{-}CO_2CH_3]^+$, 209 (8) $[M\text{-}CO_2CH_3\text{-}CH_3]^+$; **HRMS** (ESI) calcd. for $C_{17}H_{15}FNO_2^+$ $[M+H]^+$ 284.1081, found 284.1083.

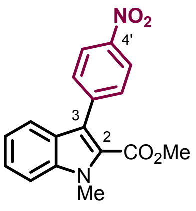

6l, 59%

Methyl 1-methyl-3-(4-nitrophenyl)-1*H*-indole-2-carboxylate (**6k**): prepared from indole **4a** (2.27 mg, 12.0 µmol) and 4-nitrophenylboronic acid pinacol ester (3.59 mg, 14.4 µmol) in MeCN applying **GP-2**; yellow solid (2.19 mg, 7.06 µmol, 59%); **m.p.** = 151 °C (EtOAc); $\mathbf{R_f}$ = 0.50 (silica gel, pentane/EtOAc 80:20); ^{1}**H NMR** (400 MHz, $CDCl_3$) δ = 8.27 – 8.34 (m, 2H), 7.57 – 7.64 (m, 2H), 7.52 (dt, *J* = 8.2, 1.0 Hz, 1H), 7.40 – 7.50 (m, 2H), 7.20 (ddd, *J* = 8.0, 6.4, 1.4 Hz, 1H), 4.11 (s, 3H), 3.73 (s, 3H) ppm; ^{13}**C NMR** (75 MHz, $CDCl_3$) δ = 162.6, 146., 126.0, 125.9, 125.1, 123.2, 122.0, 121.6, 120.9, 110.6, 51.72, 32.28 ppm; **IR** (film) $\tilde{\upsilon}_{max}$ = 2949, 1694, 1507, 1440, 1340, 1321, 1263, 1244, 1200, 1108, 1087, 850, 743, 706, 694 cm^{-1}; **MS** (EI, 70 eV): *m/z* (%) = 310 (100) $[M]^+$, 279 (4) $[M\text{-}OCH_3]^+$, 264 (4) $[M\text{-}OCH_3\text{-}CH_3]^+$, 205 (17) $[M\text{-}CO_2CH_3\text{-}NO_2]^+$, 190 (9) $[M\text{-}CO_2CH_3\text{-}NO_2\text{-}CH_3]^+$; **HRMS** (ESI) calcd. for $C_{17}H_{15}N_2O_4^+$ $[M+H]^+$ 311.1026, found 311.1027.

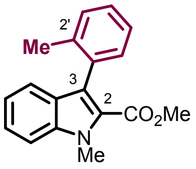

6m, 45%

Methyl 1-methyl-3-(2'-tolyl)-1*H*-indole-2-carboxylate (**6m**): prepared from indole **4a** (2.27 mg, 12.0 µmol) and *o*-tolylboronic acid (1.96 mg, 14.4 µmol) in MeCN applying **GP-2**; colorless oil (1.51 mg, 5.41 µmol, 45%); $\mathbf{R_f}$ = 0.50 (silica gel, pentane/EtOAc 97:3); ^{1}**H NMR** (400 MHz, $CDCl_3$) δ = 7.36 – 7.46 (m, 2H), 7.28 – 7.33 (m, 3H), 7.18 – 7.25 (m, 2H), 7.11 (ddd, *J* = 7.9, 6.6, 1.2 Hz, 1H), 4.12 (s, 3H), 3.63 (s, 3H), 2.09 (s, 3H) ppm; ^{13}**C NMR** (101 MHz, $CDCl_3$) δ = 163.2, 138.7, 137.3, 134.7, 130.8, 129.6, 127.3, 126.9, 125.5, 125.2, 125.0, 124.5, 122.0, 120.6, 110.2, 51.51, 32.21, 20.24 ppm; **IR** (film) $\tilde{\upsilon}_{max}$ = 3058, 2949, 2926, 2855, 1702, 1439, 1365, 1255, 1245, 1206, 1194, 1118, 1105, 1083, 739, 725 cm^{-1}; **MS** (EI, 70 eV): *m/z* (%) = 279 (100) $[M]^+$, 248 (33) $[M\text{-}OCH_3]^+$, 233 (8) $[M\text{-}OCH_3\text{-}CH_3]^+$, 220 (67) $[M\text{-}CO_2CH_3]^+$, 204 (29) $[M\text{-}CO_2CH_3\text{-}CH_3]^+$; **HRMS** (ESI) calcd. for $C_{18}H_{18}NO_2^+$ $[M+H]^+$ 280.1332, found 280.1333.

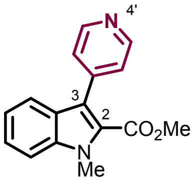

6n, 17% (71%)

Methyl 1-methyl-3-(pyridin-4'-yl)-1*H*-indole-2-carboxylate (**6n**): prepared from indole **4a** (2.27 mg, 12.0 µmol) and pyridin-4-ylboronic acid (1.77 mg, 14.4 µmol) in MeCN applying **GP-2**; yellow solid (0.54 mg, 2.04 µmol, 17%, 71% based on recovered **5a**); **m.p.** = 81 °C (EtOAc); $\mathbf{R_f}$ = 0.38 (silica gel, EtOAc); ^{1}**H NMR** (400 MHz, $CDCl_3$) δ = 8.75 – 8.98 (m, 2H), 7.89 (d, *J* = 5.0 Hz, 2H), 7.46 – 7.59 (m, 3H), 7.30 (ddd, *J* = 8.1, 6.5, 1.5 Hz, 1H), 4.13 (s, 3H), 3.81 (s, 3H) ppm; ^{13}**C NMR** (101 MHz, $CDCl_3$) δ = 161.7, 152.7, 141.4, 138.7, 127.8, 126.6 , 126.3, 125.1, 123.1, 119.8, 118.0, 111.3, 52.31, 32.67 ppm; **IR** (film) $\tilde{\upsilon}_{max}$ = 2945, 2925, 2853, 1698, 1262, 1247, 1200, 1095, 741 cm^{-1}; **MS** (EI, 70 eV): *m/z* (%) = 266 (100) $[M]^+$, 251 (4) $[M\text{-}CH_3]^+$, 235 (28) $[M\text{-}OCH_3]^+$, 221 (4) $[M\text{-}H\text{-}OCH_3\text{-}CH_3]^+$, 207 (9) $[M\text{-}CO_2CH_3]^+$, 192 (9) $[M\text{-}CO_2CH_3\text{-}CH_3]^+$; **HRMS** (ESI) calcd. for $C_{16}H_{15}N_2O_2^+$ $[M+H]^+$ 267.1128, found 267.1128.

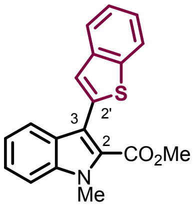

6o, 53% (66%)

Methyl 3-(benzo[*b*]thiophen-2'-yl)-1-methyl-1*H*-indole-2-carboxylate (6o): prepared from indole **4a** (2.27 mg, 12.0 µmol) and benzo[*b*]thiophen-2-ylboronic acid (2.56 mg, 14.4 µmol) in MeCN applying **GP-2**; off-white solid (2.04 mg, 6.36 µmol, 53%, 66% based on recovered **5a**); **m.p.** = 114 °C (EtOAc); $\mathbf{R_f}$ = 0.58 (silica gel, pentane/EtOAc 91:9); **^{1}H NMR** (400 MHz, $CDCl_3$) δ = 7.74 – 7.94 (m, 3H), 7.30 – 7.48 (m, 5H), 7.21 (ddd, *J* = 8.0, 6.1, 1.9 Hz, 1H), 4.09 (s, 3H), 3.81 (s, 3H) ppm; **^{13}C NMR** (101 MHz, $CDCl_3$) δ = 162.8, 140.8, 140.2, 138.5, 136.1, 127.1, 126.2, 125.7), 124.2), 124.2), 124.0, 123.5, 122.1, 121.8, 121.4, 116.2, 110.4, 51.90, 32.24 ppm; **IR** (film) $\tilde{\upsilon}_{max}$ = 3057, 3006, 2952, 1700, 1255, 1247, 754, 743, 727 cm^{-1}; **MS** (EI, 70 eV): *m/z* (%) = 321 (100) $[M]^+$, 290 (4) $[M\text{-}CH_3]^+$, 275 (2) $[M\text{-}OCH_3\text{-}CH_3]^+$, 262 (17) $[M\text{-}CO_2CH_3]^+$, 246 (17) $[M\text{-}H\text{-}CO_2CH_3\text{-}CH_3]^+$; **HRMS** (EI) calcd. for $C_{19}H_{15}NO_2S$ [M] 321.0818, found 321.0816.

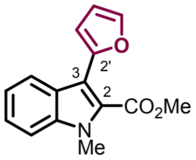

6p, 96%

Methyl 3-(furan-2'-yl)-1-methyl-1*H*-indole-2-carboxylate (6p): prepared from indole **4a** (2.27 mg, 12.0 µmol) and furan-3-ylboronic acid (1.61 mg, 14.4 µmol) in MeCN applying **GP-2**; yellow solid (2.94 mg, 11.5 µmol, 96%); **m.p.** = 68 °C (EtOAc); $\mathbf{R_f}$ = 0.50 (silica gel, pentane/EtOAc 91:9); **^{1}H NMR** (400 MHz, $CDCl_3$) δ = 7.67 – 7.75 (m, 2H), 7.53 (virt. t, $J_{virt.}$ = 1.7 Hz, 1H), 7.35 – 7.45 (m, 2H), 7.17 (ddd, *J* = 8.0, 5.3, 2.6 Hz, 1H), 6.63 (dd, *J* = 1.8, 0.8 Hz, 1H), 4.05 (s, 3H), 3.86 (s, 3H) ppm; **^{13}C NMR** (101 MHz, $CDCl_3$) δ = 163.0, 142.3, 141.2, 138.8, 126.6, 125.6, 125.1, 121.7, 120.9, 118.1, 115.1, 113.0, 110.3, 51.62, 32.31 ppm; **IR** (film) $\tilde{\upsilon}_{max}$ = 2954, 2926, 1701, 1247, 1208, 1105, 1021, 869, 790, 737 cm^{-1}; **MS** (EI, 70 eV): *m/z* (%) = 255 (100) $[M]^+$, 224 (6) $[M\text{-}OCH_3]^+$, 196 (11) $[M\text{-}CO_2CH_3]^+$; **HRMS** (ESI) calcd. for $C_{15}H_{14}NO_3^+$ $[M+H]^+$ 256.0968, found 256.0969.

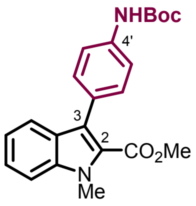

6q, 46%

Methyl 3-(4'-((tert-butoxycarbonyl)amino)phenyl)-1-methyl-1*H*-indole-2-carboxylate (6q): prepared from indole **4a** (2.27 mg, 12.0 µmol) and (4-((*tert*-butoxycarbonyl)amino)phenyl)boronic acid (3.41 mg, 14.4 µmol) in MeCN applying **GP-2**; colorless solid (2.10 mg, 5.52 µmol, 46%); **m.p.** = 152 °C (EtOAc); $\mathbf{R_f}$ = 0.19 (silica gel, pentane/EtOAc 91:9); **^{1}H NMR** (400 MHz, $CDCl_3$) δ = 7.56 (dt, *J* = 8.1, 1.0 Hz, 1H), 7.34 – 7.46 (m, 6H), 7.13 (ddd, *J* = 8.0, 6.4, 1.5 Hz, 1H), 6.54 (s, 1H), 4.06 (s, 3H), 3.71 (s, 3H), 1.55 (s, 9H) ppm; **^{13}C NMR** (101 MHz, $CDCl_3$) δ = 163.3, 153.0, 138.7, 137.3, 131.1, 129.4, 127.4, 126.8, 125.5, 124.7, 124.3, 121.8, 120.8, 118.1, 110.2, 80.71, 51.54, 32.14, 28.53 ppm; **IR** (film) $\tilde{\upsilon}_{max}$ = 3375, 3328, 2980, 2936, 1697, 1521, 1262, 1235, 1153, 1056, 748 cm^{-1}; **MS** (ESI): m/z (%) = 380 $[M+H]^+$; **HRMS** (ESI) calcd. for $C_{22}H_{25}N_2O_4^+$ $[M+H]^+$ 381.1809, found 381.1809.

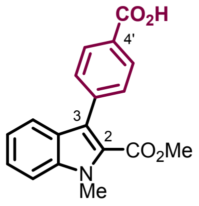

6r, 24%

4'-(2-(Methoxycarbonyl)-1-methyl-1*H*-indol-3-yl)benzoic acid (**6r**): prepared from indole **4a** (2.27 mg, 12.0 µmol) and 4-carboxyphenylboronic acid (2.39 mg, 14.4 µmol) in MeCN applying **GP-2**; colorless solid (0.84 mg, 2.88 µmol, 24%); **m.p.** = 176 °C (EtOAc); $\mathbf{R_f}$ = 0.51 (silica gel, DCM/MeOH 95:5); ^{1}**H NMR** (400 MHz, acetone-d_6) δ = 8.16 – 8.29 (m, 2H), 7.52 – 7.64 (m, 3H), 7.36 – 7.51 (m, 2H), 7.18 (ddd, *J* = 8.0, 6.5, 1.4 Hz, 1H), 4.10 (s, 3H), 3.72 (s, 3H) ppm; ^{13}**C NMR** (75 MHz, acetone-d_6) δ = 167.6, 163.3, 140.5, 139.4, 131.2, 130.1, 129.7, 126.8, 126.2, 126.2, 123.1, 122.0, 121.6, 111.6, 51.80, 32.28 ppm; **IR** (film) $\tilde{\upsilon}_{max}$ = 2952, 2850, 2665, 2547, 2227, 2065, 1682, 1260, 1242, 1171, 1126, 1109, 1088, 869, 739, 705 cm^{-1}; **MS** (ESI): m/z (%) = 310 $[M+H]^+$, 278 $[M-OCH_3]$; **HRMS** (ESI) calcd. for $C_{18}H_{14}NO_4^-$ $[M-H^+]^-$ 308.0928, found 308.0930.

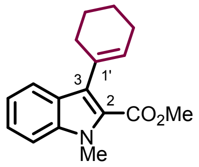

6s 94%

Methyl 3-(cyclohex-1'-en-1'-yl)-1-methyl-1*H*-indole-2-carboxylate (**6s**): prepared from indole **4a** (2.27 mg, 12.0 µmol) and cyclohex-1-en-1-ylboronic acid (1.81 mg, 14.4 µmol) in MeCN applying **GP-2**; colorless oil (3.04 mg, 11.3 µmol, 94%); $\mathbf{R_f}$ = 0.45 (silica gel, pentane/EtOAc 97:3); ^{1}**H NMR** (400 MHz, $CDCl_3$) δ = 7.61 – 7.70 (m, 1H), 7.31 – 7.40 (m, 2H), 7.13 (ddd, *J* = 8.0, 4.5, 3.3 Hz, 1H), 5.64 – 5.77 (m, 1H), 4.01 (s, 3H), 3.90 (s, 3H), 2.17 – 2.36 (m, 4H), 1.68 – 1.88 (m, 4H) ppm; ^{13}**C NMR** (101 MHz, $CDCl_3$) δ = 163.3, 138.7, 132.0, 128.2, 127.0, 126.4, 125.3, 124.0, 121.8, 120.2, 110.0, 51.65, 31.99, 30.69, 25.81, 23.51, 22.55 ppm; **IR** (film) $\tilde{\upsilon}_{max}$ = 2926, 2854, 2834, 1701, 1438, 1369, 1245, 1191, 1146, 1104, 1079, 738 cm^{-1}; **MS** (EI, 70 eV): *m/z* (%) = 269 (100) $[M]^+$, 254 (15) $[M-CH_3]^+$, 237 (21) $[M-H-OCH_3]^+$, 210 (45) $[M-CO_2CH_3]^+$, 194 (9) $[M-H-CO_2CH_3-CH_3]^+$; **HRMS** (EI) calcd. for $C_{17}H_{19}NO_2$ [M] 269.1410, found 269.1410.

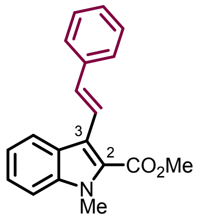

6t, 36% (81%)

Methyl (*E*)-1-methyl-3-styryl-1*H*-indole-2-carboxylate (**6t**): prepared from indole **4a** (2.27 mg, 12.0 µmol) and (*E*)-styrylboronic acid (2.13 mg, 14.4 µmol) in MeCN applying **GP-2**; yellow solid (1.26 mg, 4.32 µmol, 36%, 81% based on recovered **5a**); **m.p.** = 83 °C (EtOAc); $\mathbf{R_f}$ = 0.26 (silica gel, pentane/EtOAc 97:3); ^{1}**H NMR** (400 MHz, $CDCl_3$) δ = 8.12 (dt, *J* = 8.2, 1.0 Hz, 1H), 7.88 (d, *J* = 16.6 Hz, 1H), 7.55 – 7.62 (m, 2H), 7.37 – 7.43 (m, 4H), 7.23 – 7.31 (m, 3H), 4.04 (s, 3H), 4.01 (s, 3H) ppm; ^{13}**C NMR** (101 MHz, $CDCl_3$) δ = 163.2, 139.5, 138.4, 131.0, 128.8, 127.4, 126.4, 125.7, 125.7, 124.8, 122.6, 122.6, 121.5, 121.2, 110.6, 51.95, 32.43 ppm; **IR** (film) $\tilde{\upsilon}_{max}$ = 3651, 3631, 3375, 3050, 1690, 1467, 1440, 1237, 1128, 1107, 966, 738, 690 cm^{-1}; **MS** (EI, 70 eV): *m/z* (%) = 291 (100) $[M]^+$, 260 (6) $[M-H-OCH_3]^+$, 245 (4) $[M-OCH_3-CH_3]^+$, 232 (26) $[M-CO_2CH_3]^+$, 217 (51) $[M-CO_2CH_3-CH_3]^+$; **HRMS** (ESI) calcd. for $C_{19}H_{18}NO_2^+$ $[M+H]^+$ 292.1332, found 292.1332.

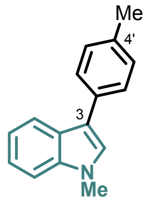

6u, 57% (quant.)

1-Methyl-3-(4'-tolyl)-1*H*-indole (**6u**): prepared from 1-methyl-1*H*-indole (**4u**, 1.57 mg, 12.0 µmol) and *p*-tolylboronic acid (1.96 mg, 14.4 µmol) in MeCN applying **GP-2**; colorless oil (1.51 mg, 6.84 µmol, 57%; quantitative yield based on recovered **4u**); **R_f** = 0.59 (silica gel, pentane/EtOAc 97:3); **^{1}H NMR** (500 MHz, MeOD) δ = 7.83 (d, *J* = 8.0 Hz, 1H), 7.55 – 7.49 (m, 2H), 7.40 (d, *J* = 8.3 Hz, 1H), 7.35 (s, 1H), 7.24 – 7.19 (m, 3H), 7.10 (ddd, *J* = 8.0, 7.0, 1.0 Hz, 1H), 3.83 (s, 3H), 2.36 (s, 3H) ppm; **^{13}C NMR** (126 MHz, MeOD) δ = 139.0, 136.1, 134.3, 130.3, 128.0, 127.5, 127.4, 122.7, 120.6, 120.5, 117.4, 110.5, 32.86, 21.16 ppm; **MS** (EI, 70 eV): *m/z* (%) = 221 (100) $[M]^+$, 206 (13) $[M\text{-}CH_3]^+$.

The spectroscopic data is in accordance with that reported in the literature.[7]

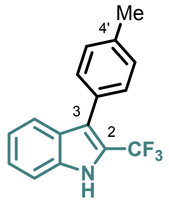

6v, 46% (quant.)

3-(4'-Tolyl)-2-(trifluoromethyl)-1*H*-indole (**6v**): prepared from 2-(trifluoromethyl)-1*H*-indole (**4v**, 2.22 mg, 12.0 µmol) and *p*-tolylboronic acid (1.96 mg, 14.4 µmol) in THF applying **GP-2**; colorless solid (1.52 mg, 5.52 µmol, 46%, quantitative yield based on recovered **4v**); **m.p.** = 98 - 101 °C (EtOAc); **R_f** = 0.48 (silica gel, pentane/EtOAc 97:3); **^{1}H NMR** (400 MHz, $CDCl_3$) δ = 8.46 (s, 1H), 7.67 (d, *J* = 8.1 Hz, 1H), 7.50 – 7.41 (m, 3H), 7.40 – 7.35 (m, 1H), 7.30 (d, *J* = 7.8 Hz, 2H), 7.21 (ddd, *J* = 8.2, 6.9, 1.0 Hz, 1H), 2.45 (s, 3H) ppm; **^{13}C NMR** (101 MHz, $CDCl_3$) δ = 137.4, 135.1, 129.9 (q, *J* = 1.0 Hz), 129.3, 129.2, 127.6, 125.2, 121.9 (q, *J* = 270.7 Hz), 121.3, 121.3, 121.2 (q, *J* = 36.7 Hz), 120.0 (q, *J* = 3.0 Hz), 111.8, 21.43 ppm; **^{19}F NMR** (376 MHz, $CDCl_3$) δ = -56.80 (s, CF_3) ppm; **MS** (EI, 70 eV): *m/z* (%) = 275 (100) $[M]^+$, 206 (8) $[M\text{-}CF_3]^+$.

The spectroscopic data is in accordance to that reported in the literature.[8]

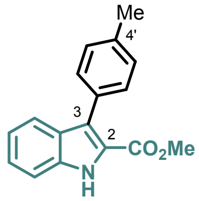

6w, 23% (quant.)

Methyl 3-(4'-tolyl)-1*H*-indole-2-carboxylate (**6w**): prepared from methyl 1*H*-indole-2-carboxylate (**4w**, 2.10 mg, 12.0 µmol) and *p*-tolylboronic acid (1.96 mg, 14.4 µmol) in THF applying **GP-2**; colorless solid (0.73 mg, 2.75 µmol, 23%, quantitative yield based on recovered **6w**); **m.p.** = 161 - 165 °C (EtOAc); **R_f** = 0.34 (silica gel, pentane/EtOAc 91:9); **^{1}H NMR** (400 MHz, $CDCl_3$) δ = 8.97 (s, 1H), 7.65 (dt, *J* = 8.2, 0.9 Hz, 1H), 7.50 – 7.41 (m, 3H), 7.36 (ddd, *J* = 8.3, 6.9, 1.2 Hz, 1H), 7.28 (d, *J* = 7.8 Hz, 2H), 7.15 (ddd, *J* = 8.1, 6.9, 1.1 Hz, 1H), 3.83 (s, 3H), 2.44 (s, 3H) ppm; **^{13}C NMR** (101 MHz, $CDCl_3$) δ = 162.5, 137.1, 135.9, 130.5, 130.5, 128.8, 128.1, 126.0, 124.7, 122.4, 122.0, 121.0, 111.8, 51.91, 21.49 ppm; **MS** (EI, 70 eV): *m/z* (%) = 265 (72) $[M]^+$, 233 (100) $[M\text{-}OCH_3\text{-}H]^+$.

The spectroscopic data is in accordance with that reported in the literature.[9]

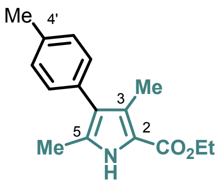

6x, 19% (quant.)

Ethyl 3,5-dimethyl-4-(4'-tolyl)-1*H*-pyrrole-2-carboxylate (**6x**): prepared from ethyl 3,5-dimethyl-1*H*-pyrrole-2-carboxylate (**4x**, 2.01 mg, 12.0 µmol) and *p*-tolylboronic acid (1.96 mg, 14.4 µmol) in THF applying **GP-2**; colorless solid (0.59 mg, 2.28 µmol, 19%, quantitative yield based on recovered **4x**); **m.p.** = 102-104 °C (EtOAc); R_f = 0.30 (silica gel, pentane/EtOAc 91:9); **^{1}H NMR** (400 MHz, $CDCl_3$) δ = 8.85 (brs, 1H), 7.24 – 7.11 (m, 4H), 4.33 (q, *J* = 7.1 Hz, 2H), 2.39 (s, 3H), 2.29 (s, 3H), 2.25 (s, 3H), 1.37 (t, *J* = 7.1 Hz, 3H) ppm; **^{13}C NMR** (101 MHz, $CDCl_3$) δ = 162.0, 135.9, 132.1, 130.1, 130.0, 129.1, 126.8, 124.7, 117.5, 59.99, 21.32, 14.75, 12.29, 11.52 ppm; **MS** (EI, 70 eV): *m/z* (%) = 257 (71) $[M]^+$, 211 (100) $[M\text{-}OCH_2CH_3\text{-}H]^+$, 184 (4) $[M\text{-}COOCH_2CH_3]^+$; **IR** (film) $\tilde{\upsilon}_{max}$ = 3286, 2989, 2960, 2923, 2855, 1657, 1439, 1274, 1204, 1100, 1023, 770, 752 cm^{-1}; **HRMS** (EI) calcd. for $C_{16}H_{19}NO_2$ [M] 257.1410, found 257.1410.

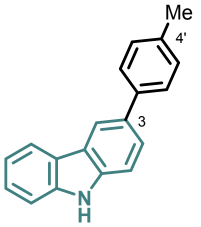

6y, 64% (89%)

3-(4'-Tolyl)-9*H*-carbazole (**6y**): prepared from 9*H*-carbazole (**4y**, 2.01 mg, 12.0 µmol) and *p*-tolylboronic acid (1.96 mg, 14.4 µmol) in THF applying **GP-2**; brown solid (1.98 mg, 7.68 µmol, 64%; 89% based on recovered **4y**); **m.p.** = 185 °C (EtOAc); R_f = 0.54 (silica gel, pentane/EtOAc 75:25); **^{1}H NMR** (400 MHz, $CDCl_3$) δ = ppm; **^{13}C NMR** (101 MHz, $CDCl_3$) δ = 140.1, 139.4, 139.0, 136.3, 133.2, 129.6, 127.3, 126.1, 125.5, 124.0, 123.6, 120.5, 119.7, 118.8, 110.9, 110.8, 21.22 ppm; **MS** (EI, 70 eV): *m/z* (%) = 257 (100) $[M]^+$, 241 (8) $[M\text{-}CH_3\text{-}H]^+$; **IR** (film) $\tilde{\upsilon}_{max}$ = 3411, 2957, 2923, 2853, 1681, 1198, 1136, 803, 728 cm^{-1}; **HRMS** (ESI) calcd. for $C_{19}H_{14}N^-$ $[M\text{-}H^+]^-$ 256.1132, found 256.1133.

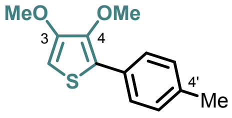

6z, 96%

3,4-Dimethoxy-2-(4'-tolyl)thiophene (**6z**): prepared from 3,4-dimethoxythiophene (**4z**, 1.73 mg, 12.0 µmol) and *p*-tolylboronic acid (1.96 mg, 14.4 µmol) in MeCN applying **GP-2**; slightly yellow oil (2.70 mg, 11.5 µmol, 96%); R_f = 0.34 (silica gel, pentane/EtOAc 95:5); **^{1}H NMR** (400 MHz, MeOD) δ = 7.54 (d, *J* = 8.2 Hz, 2H), 7.17 (d, *J* = 8.0 Hz, 2H), 6.32 (d, *J* = 1.3 Hz, 1H), 3.84 (s, 3H), 3.75 (s, 3H), 2.32 (s, 3H) ppm; **^{13}C NMR** (101 MHz, MeOD) δ = 152.6, 144.2, 138.3, 131.7, 130.3, 127.6, 126.7, 95.16, 60.53, 57.63 (CH_3), 21.19 (CH_3) ppm; **MS** (EI, 70 eV): *m/z* (%) = 234 (100) $[M]^+$, 219 (76) $[M\text{-}CH_3]$.

The spectroscopic data is in accordance with that reported in the literature.[11]

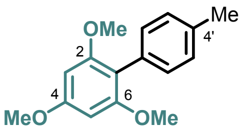

6aa, 94% (quant.)

2,4,6-Trimethoxy-4’-methylbiphenyl (**6aa**): prepared from 1,3,5-trimethoxybenzene (**4aa**, 2.02 mg, 12.0 µmol) and *p*-tolylboronic acid (1.96 mg, 14.4 µmol) in MeCN applying **GP-2**; colorless solid (2.91 mg, 11.3 µmol, 94%, quantitative yield based on recovered **4aa**); **R$_f$** = 0.29 (silica gel, pentane/EtOAc 97:3); **^{1}H NMR** (400 MHz, CDCl$_3$) δ = 7.16 – 7.25 (m, 4H), 6.23 (s, 2H), 3.86 (s, 3H), 3.72 (s, 6H), 2.38 (s, 3H) ppm; **^{13}C NMR** (75 MHz, CDCl$_3$) δ = 160.5, 158.5, 136.2, 131.1, 131.1, 128.7, 112.6, 91.02, 56.02, 55.52, 21.50 ppm; **MS** (EI, 70 eV): *m/z* (%) = 258 (100) [M]$^+$, 243 (4) [M-CH$_3$]$^+$.

The spectroscopic data is in accordance with that reported in the literature.[10]

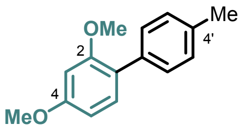

6ab, 64%

2,4-Dimethoxy-4’-methyl-1,1’-biphenyl (**6ab**): prepared from 1,3-dimethoxybenzene (**4ab**, 1.66 mg, 12.0 µmol) and *p*-tolylboronic acid (1.96 mg, 14.4 µmol) in MeCN applying **GP-2**; colorless solid (1.75 mg, 7.67 µmol, 64%); **R$_f$** = 0.54 (silica gel, pentane/EtOAc 97:3); **^{1}H NMR** (400 MHz, CDCl$_3$) δ = 7.35 – 7.42 (m, 2H), 7.21 (dd, *J* = 10.0, 8.5 Hz, 3H), 6.52 – 6.60 (m, 2H), 3.85 (s, 3H), 3.79 (s, 3H), 2.38 (s, 3H) ppm; **^{13}C NMR** (101 MHz, CDCl$_3$) δ = 160.3, 157.6, 136.3, 135.6, 131.3, 129.5, 128.9, 123.8, 104.7, 99.13, 55.67, 55.55 , 21.30 ppm; **MS** (EI, 70 eV): *m/z* (%) = 228 (100) [M]$^+$, 213 (17) [M-CH3]$^+$.

The spectroscopic data is in accordance with that reported in the literature.[11]

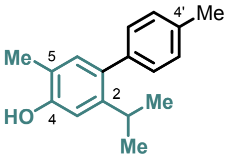

6ac, 22% (quant.)

2-Isopropyl-4',5-dimethyl-[1,1'-biphenyl]-4-ol (**6ac**): prepared from 5-isopropyl-2-methylphenol (**4ac**, 2.95 mg, 12.0 µmol) and *p*-tolylboronic acid (1.96 mg, 14.4 µmol) in MeCN applying **GP-2**; colorless solid (0.63 mg, 2.63 µmol, 22%, quantitative yield based on recovered **4ac**); **m.p.** = 58 - 62 °C (EtOAc); **R$_f$** = 0.35 (silica gel, pentane/EtOAc 95:5); **^{1}H NMR** (400 MHz, MeOD) δ = 8.95 (brs, 1H), 7.21 – 6.99 (m, 4H), 6.77 (d, *J* = 11.6 Hz, 2H), 2.94 (p, *J* = 6.9 Hz, 1H), 2.34 (s, 3H), 2.14 (s, 3H), 1.08 (d, *J* = 6.8 Hz, 6H) ppm; **^{13}C NMR** (126 MHz, MeOD) δ = 155.7, 146.0 , 140.6, 136.9, 133.8, 133.1, 130.5, 129.5, 122.4, 112.3, 30.25, 24.60, 21.41, 15.79 ppm; **MS** (EI, 70 eV): *m/z* (%) = 240 (100) [M]$^+$, 225 (100) [M-CH$_3$]$^+$; **IR** (film) $\tilde{\upsilon}_{max}$ = 3348, 3020, 2961, 2925, 2868, 2465, 1494, 1149, 822 cm^{-1}; **HRMS** (ESI) calcd. for $C_{17}H_{19}O^-$ [M-H]$^-$ 239.1441, found 239.1442.

6. NMR Spectra of Compounds

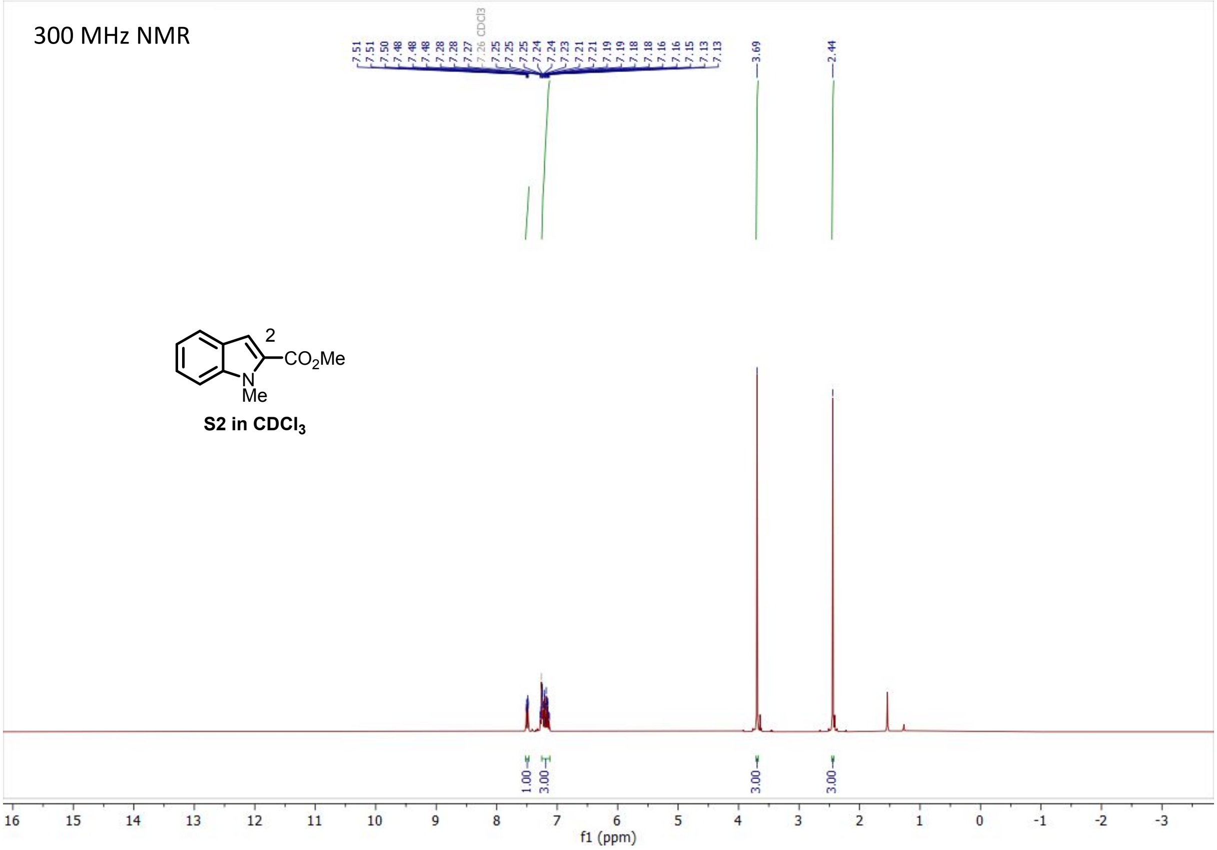

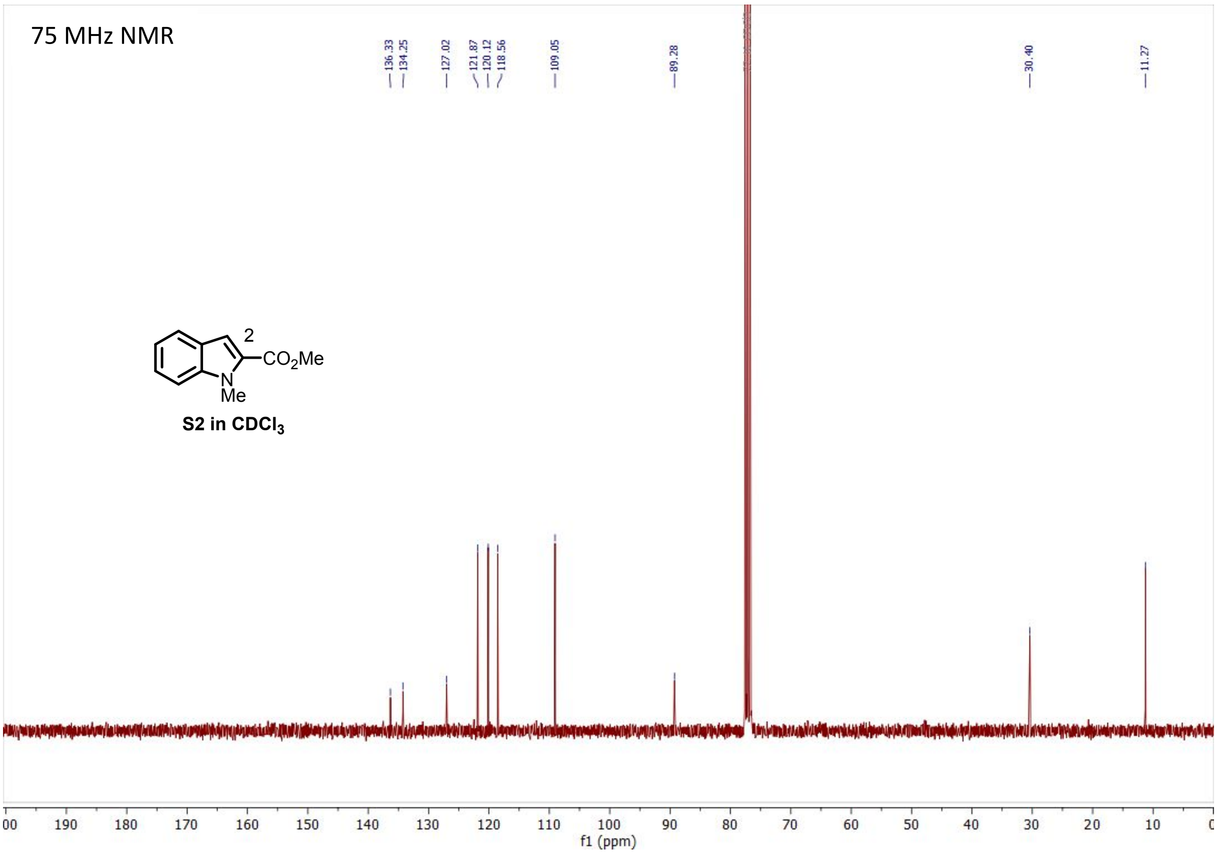

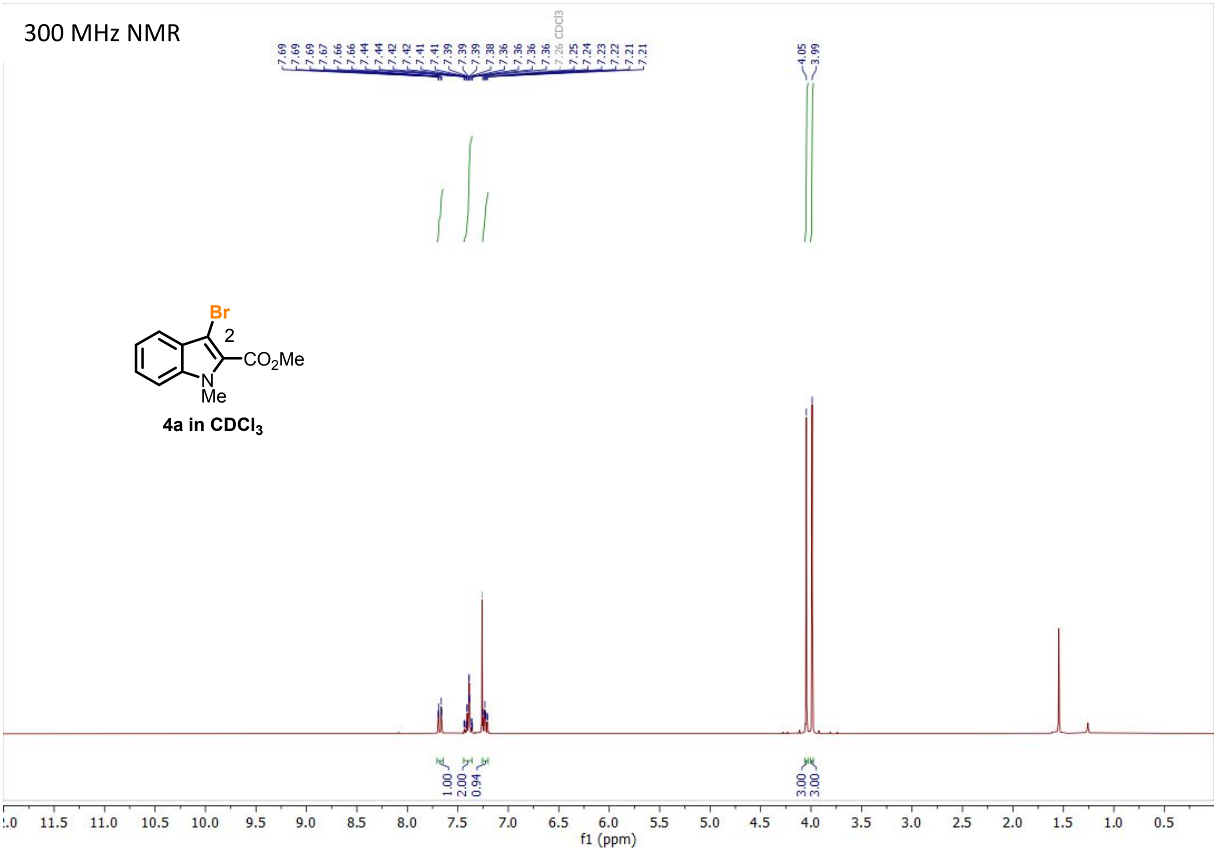

300 MHz NMR
Br
2
CO2Me
N
Me
4a in CDCl3
f1 (ppm)

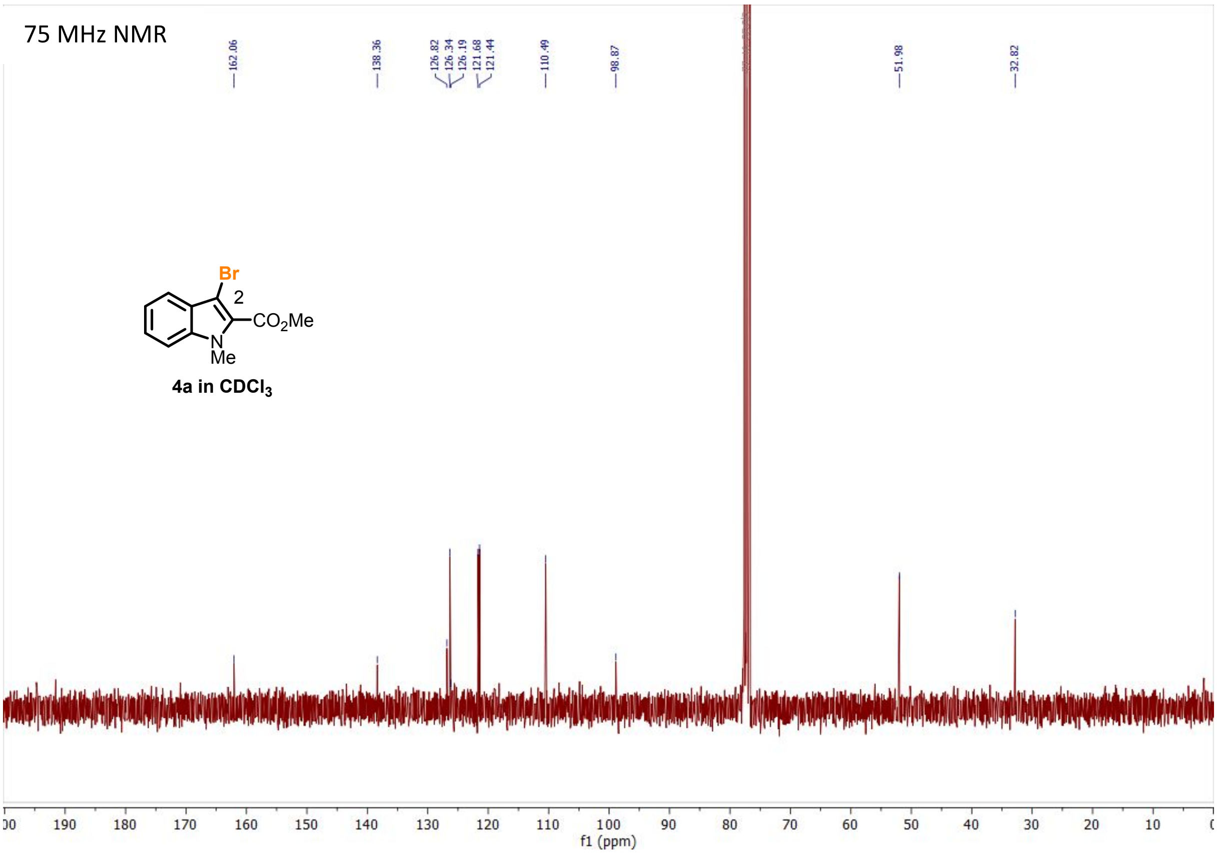

75 MHz NMR
162.06
138.36
126.82
126.34
126.19
121.68
121.44
110.49
98.87
51.98
32.82
Br
2
CO2Me
N
Me
4a in CDCl3
f1 (ppm)

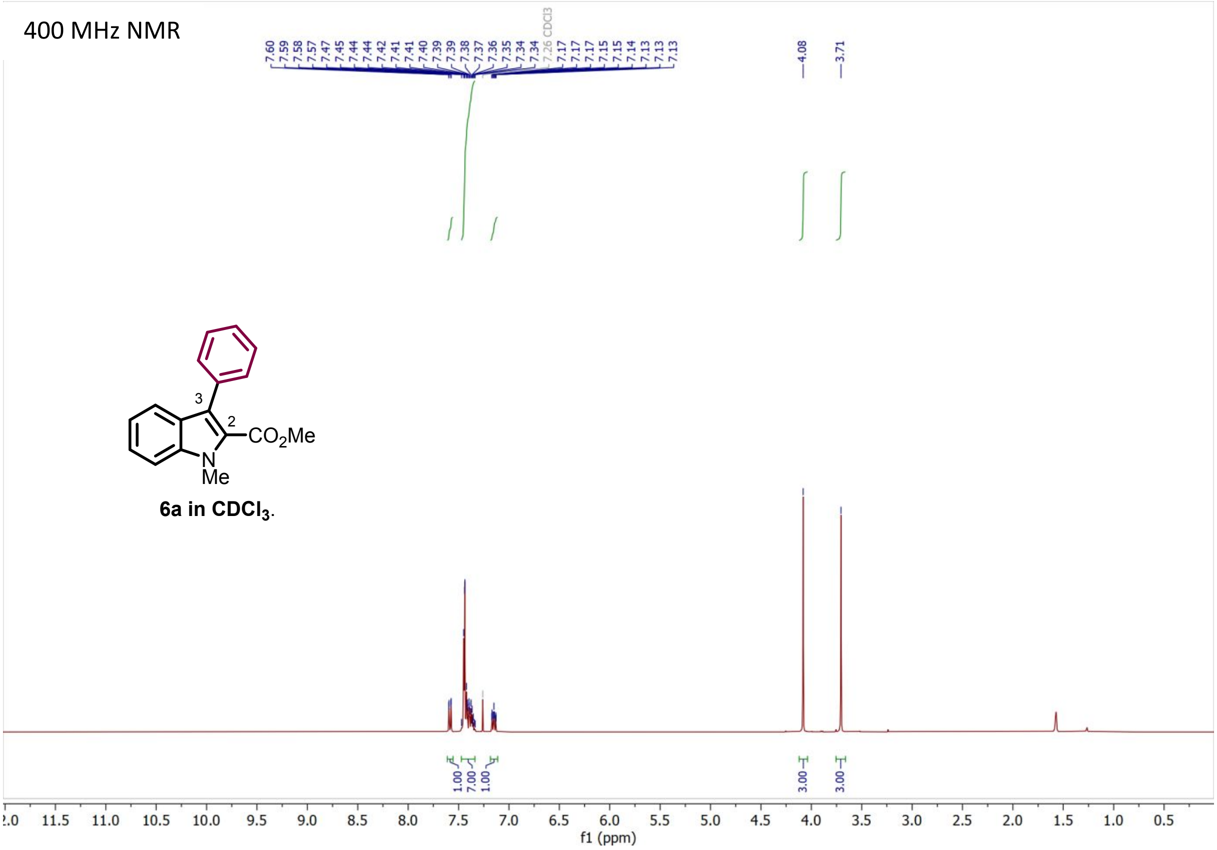
400 MHz NMR
7.60 7.59 7.58 7.57 7.47 7.45 7.44 7.44 7.42 7.41 7.41 7.40 7.39 7.39 7.38 7.37 7.36 7.35 7.34 7.34 7.26 CDCl3 7.17 7.17 7.17 7.15 7.15 7.14 7.13 7.13 7.13
4.08
3.71
3
2
CO2Me
N
Me
6a in CDCl3.
1.00
7.00
1.00
3.00
3.00
f1 (ppm)

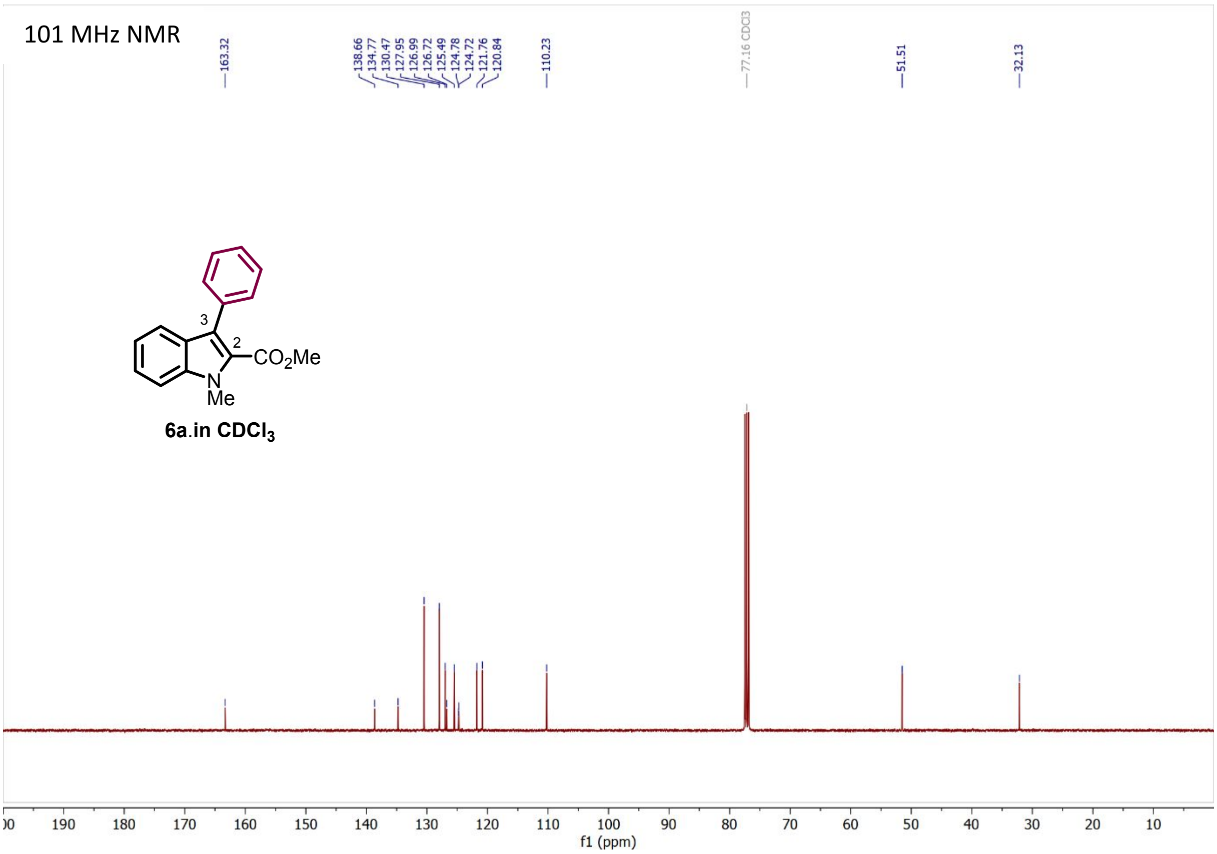
101 MHz NMR
163.32
138.66 134.77 130.47 127.95 126.99 126.72 125.49 124.78 124.72 121.76 120.84
110.23
77.16 CDCl3
51.51
32.13
3
2
CO2Me
N
Me
6a.in CDCl3
f1 (ppm)

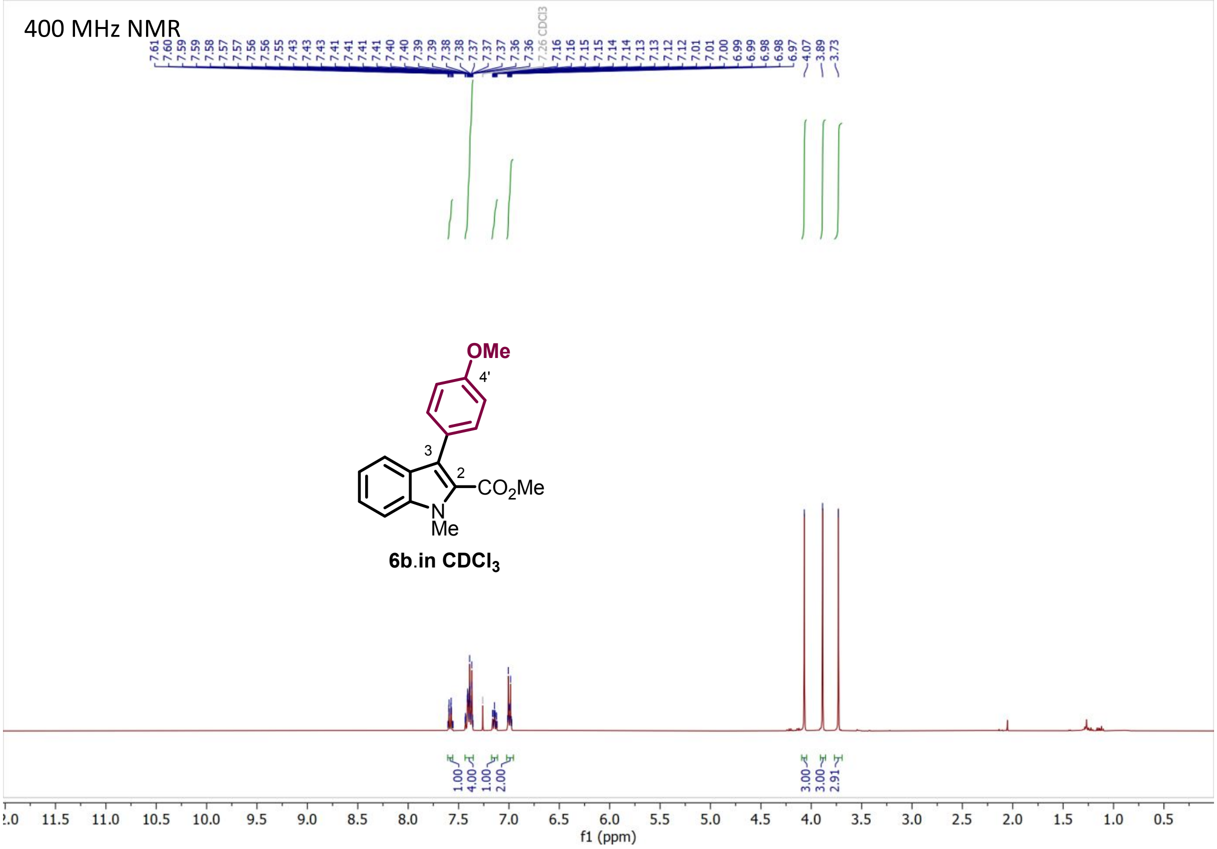
400 MHz NMR
OMe
4'
3
2
CO2Me
N
Me
6b.in CDCl3
f1 (ppm)

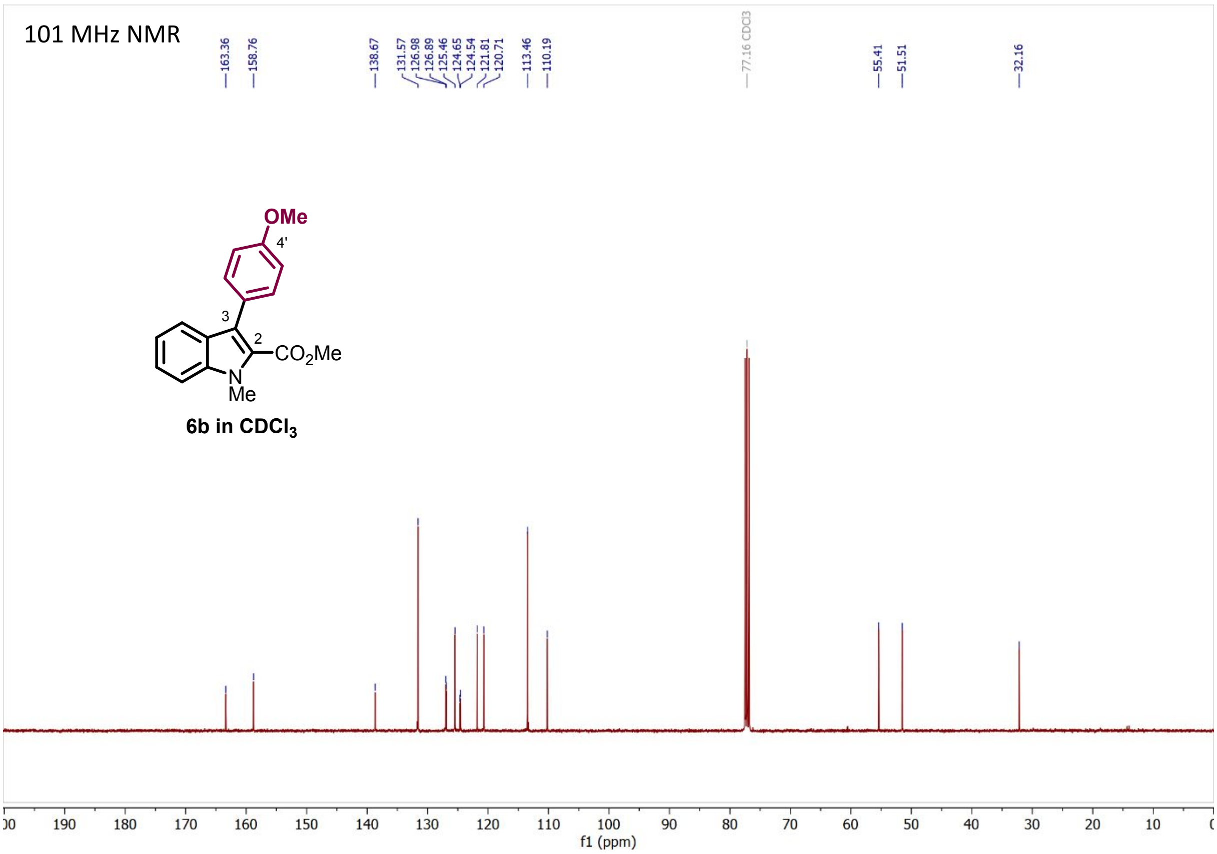
101 MHz NMR
163.36
158.76
138.67
131.57
126.98
126.89
125.46
124.65
124.54
121.81
120.71
113.46
110.19
77.16 CDCl3
55.41
51.51
32.16
OMe
4'
3
2
CO2Me
N
Me
6b in CDCl3
f1 (ppm)

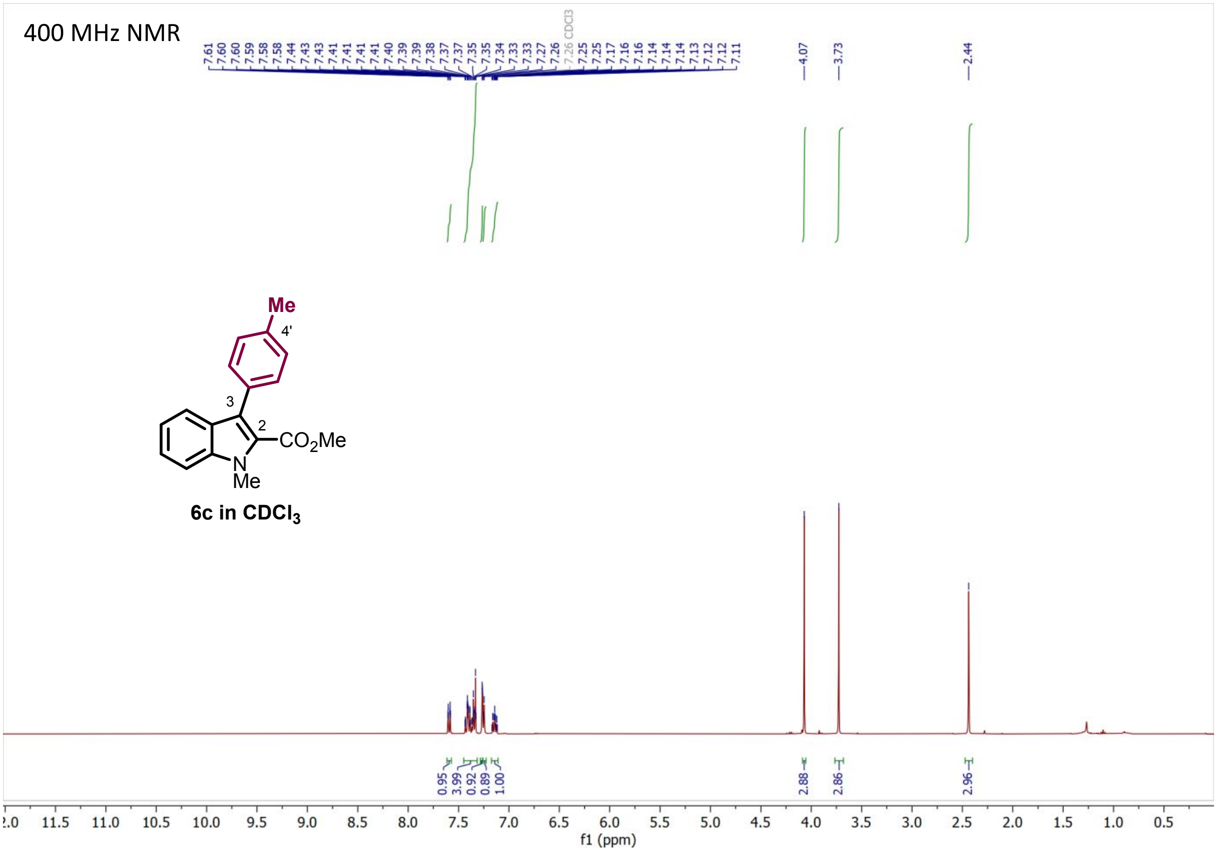

400 MHz NMR
6c in CDCl3
f1 (ppm)

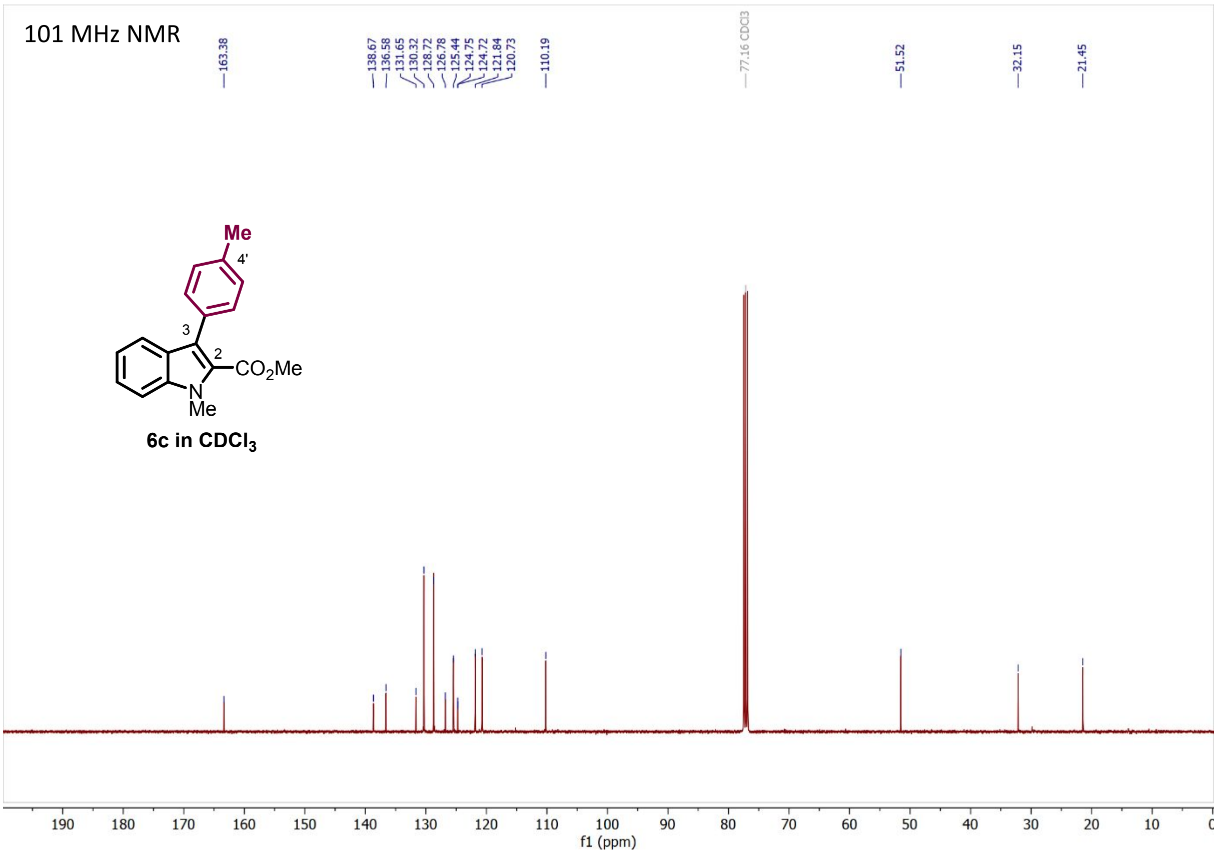

101 MHz NMR
6c in CDCl3
f1 (ppm)

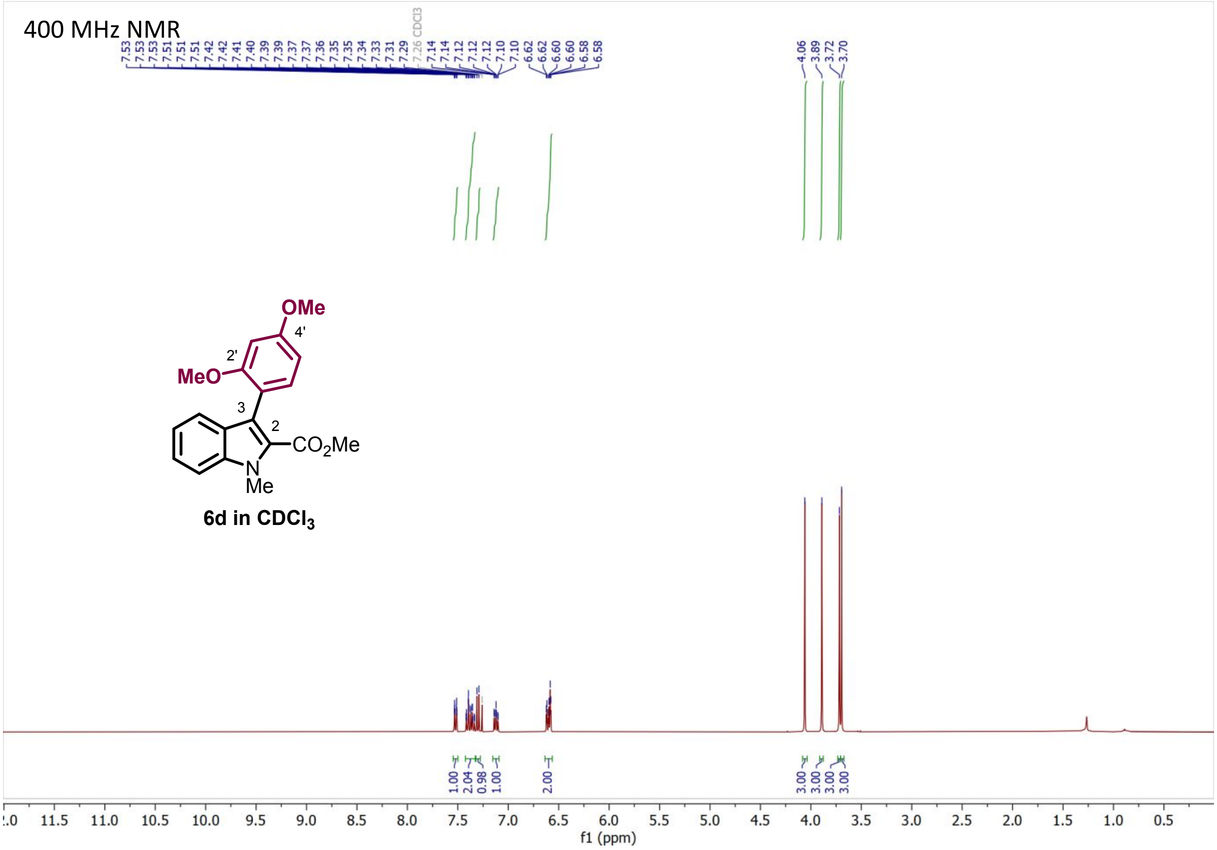
400 MHz NMR
6d in CDCl3
f1 (ppm)

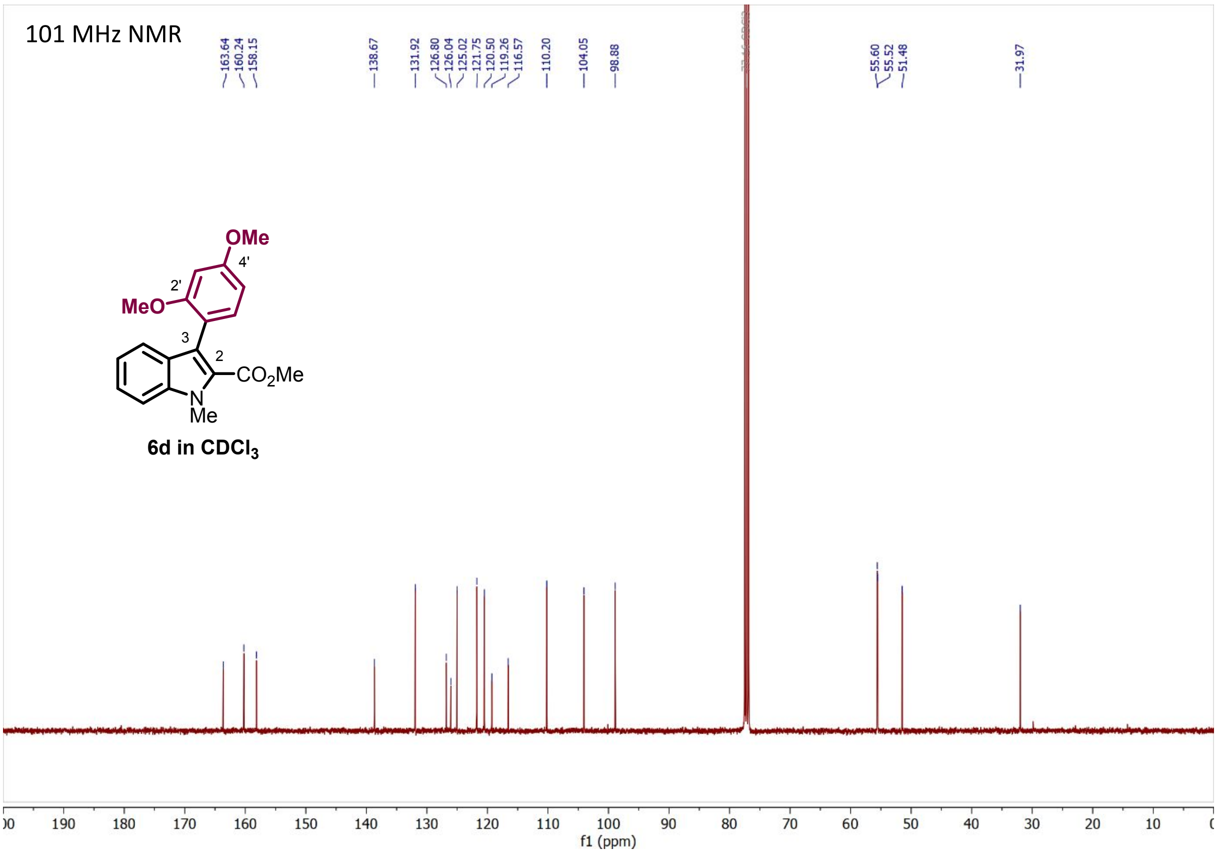
101 MHz NMR
6d in CDCl3
f1 (ppm)

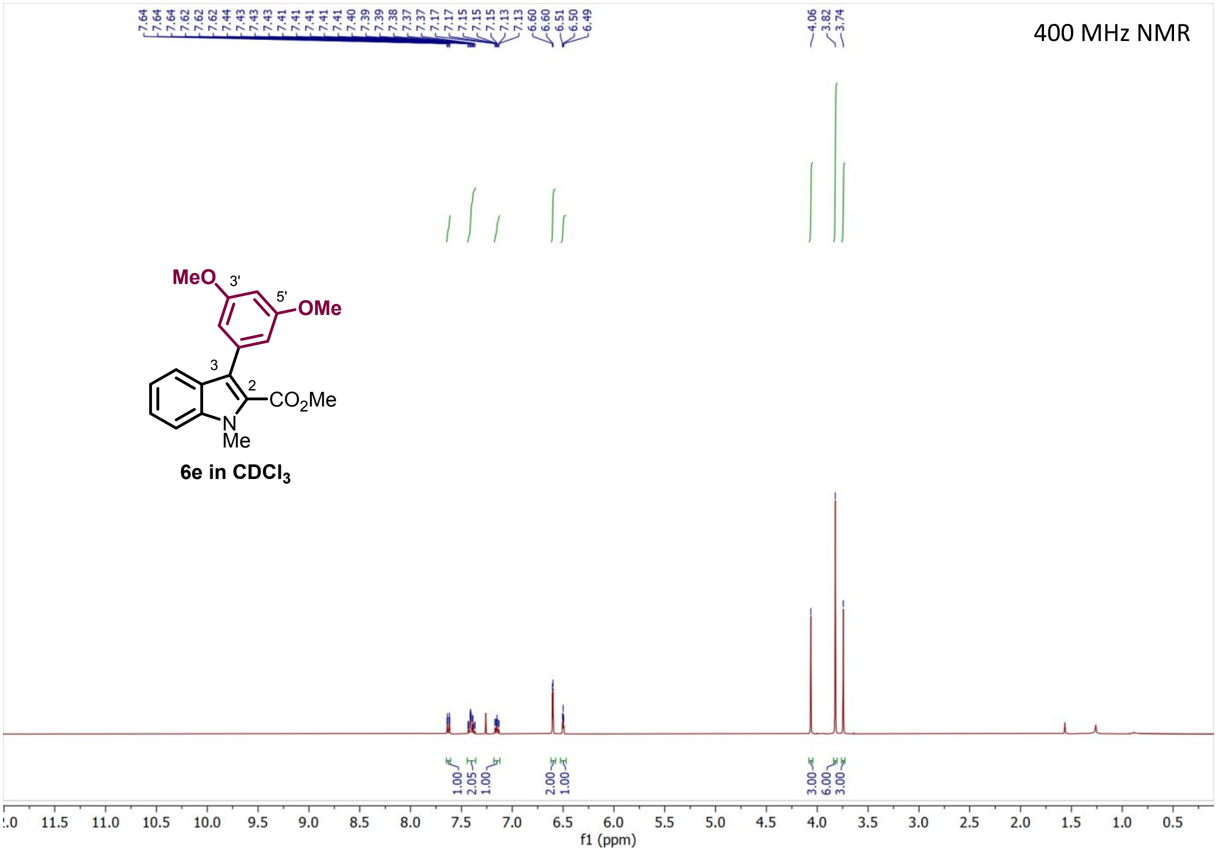

400 MHz NMR
7.64
7.64
7.64
7.62
7.62
7.62
7.44
7.43
7.43
7.43
7.41
7.41
7.41
7.41
7.41
7.40
7.39
7.39
7.38
7.37
7.37
7.17
7.15
7.15
7.15
7.13
6.60
6.60
6.51
6.50
6.49
4.06
3.82
3.74
MeO
3'
5'
OMe
3
2
CO_2Me
N
Me
6e in $CDCl_3$
1.00
2.05
1.00
2.00
1.00
3.00
6.00
3.00
f1 (ppm)

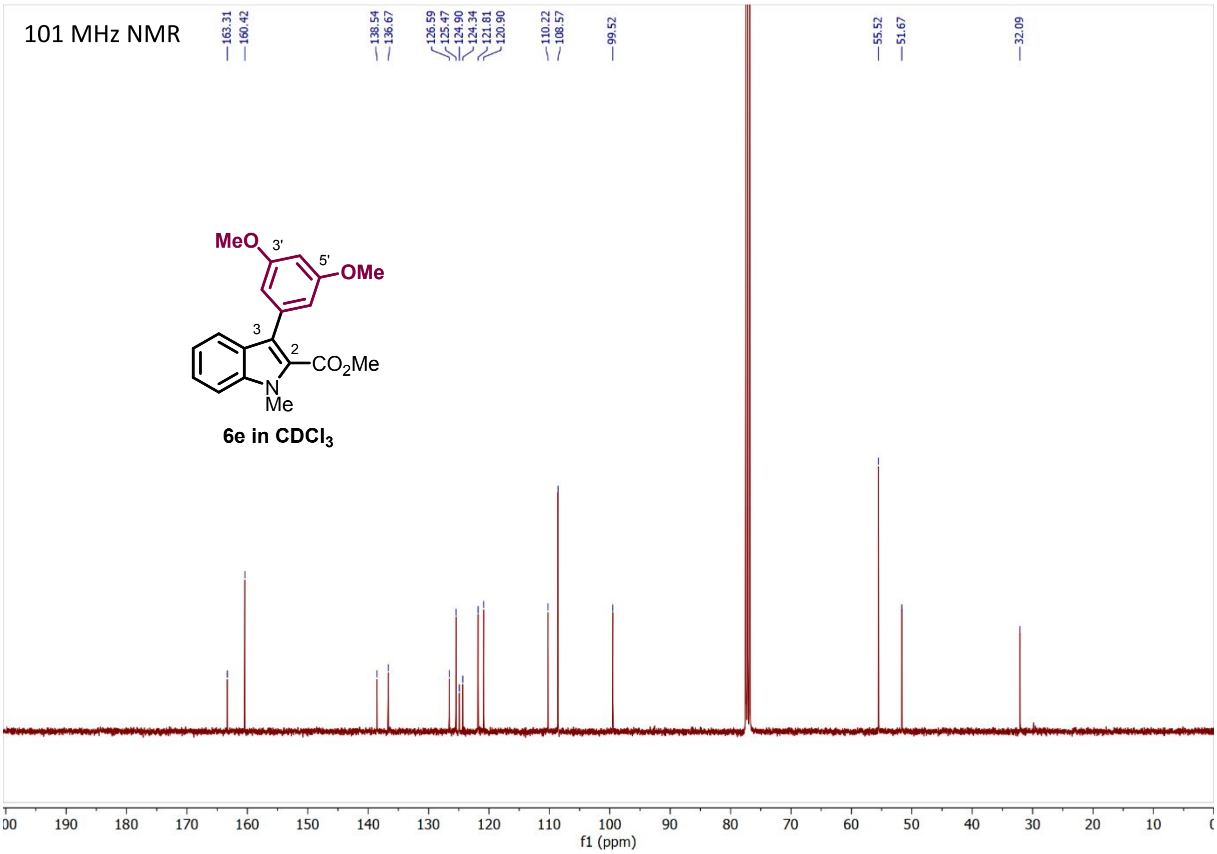

101 MHz NMR
163.31
160.42
138.54
136.67
126.59
125.47
124.90
124.34
121.81
120.90
110.22
108.57
99.52
55.52
51.67
32.09
MeO
3'
5'
OMe
3
2
CO_2Me
N
Me
6e in $CDCl_3$
f1 (ppm)

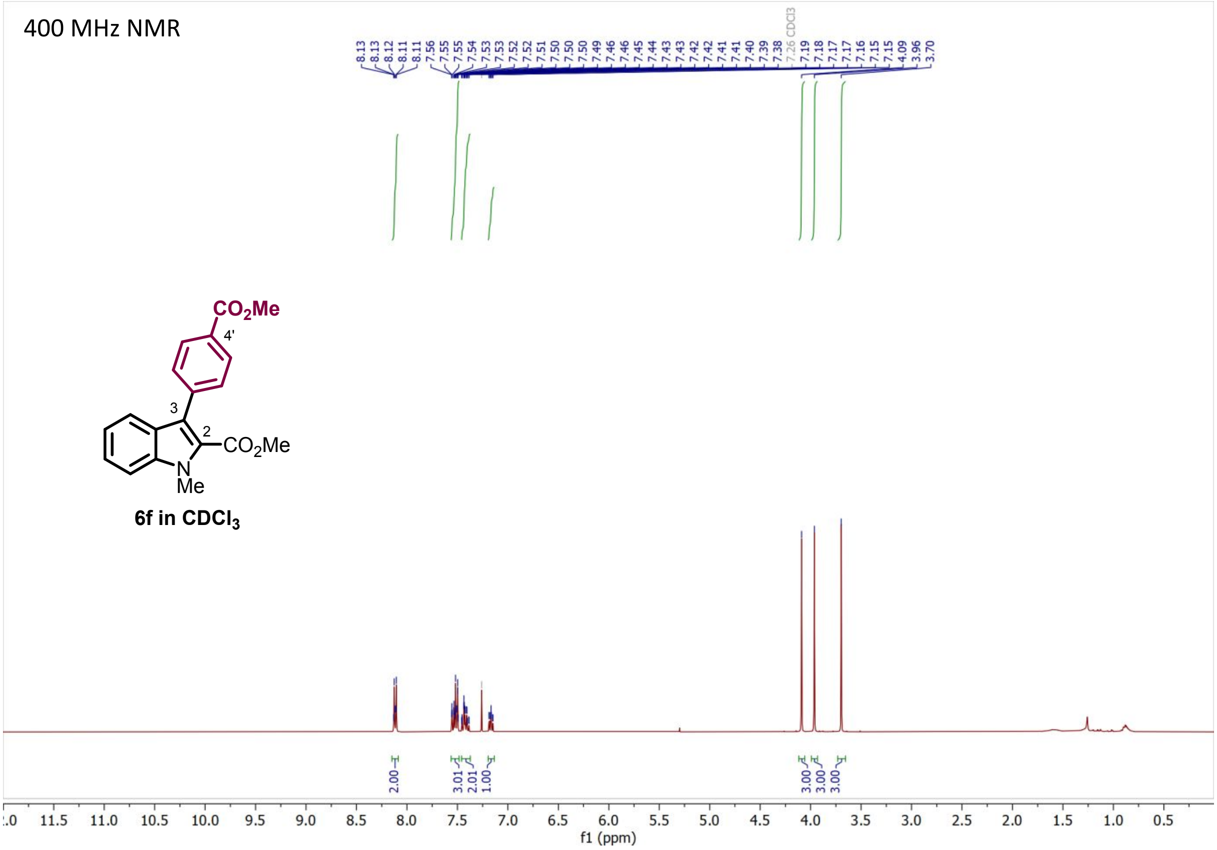

400 MHz NMR
6f in CDCl3
f1 (ppm)

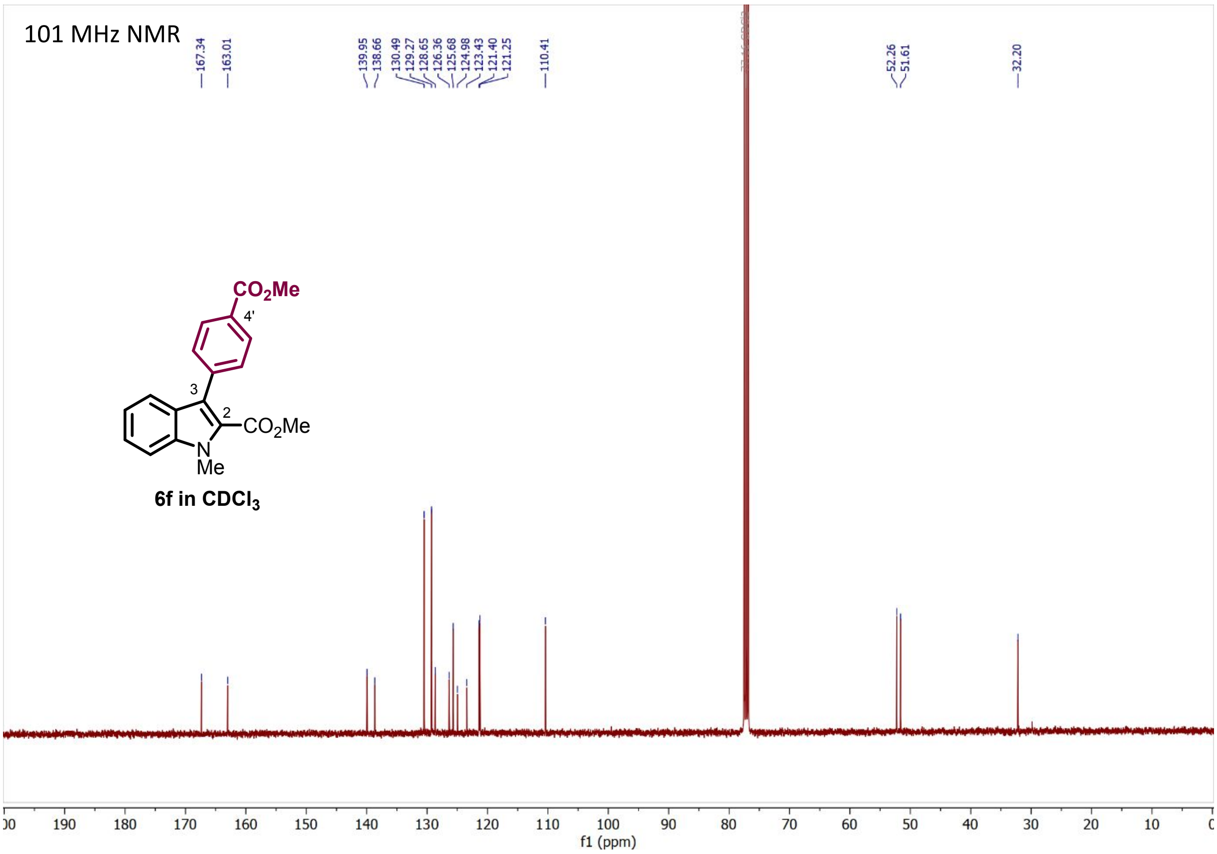

101 MHz NMR
6f in CDCl3
f1 (ppm)

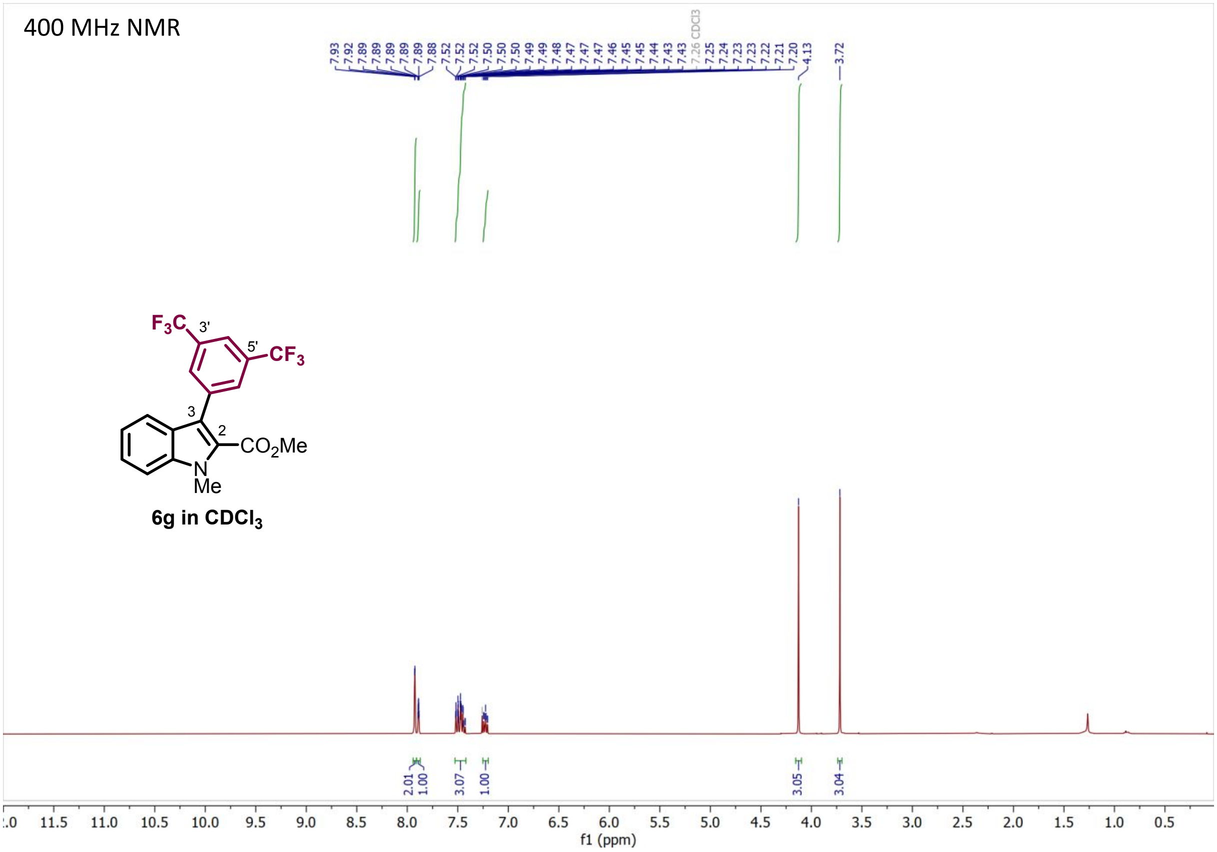
400 MHz NMR
F3C
3'
5'
CF3
3
2
CO2Me
N
Me
6g in CDCl3
7.93
7.92
7.89
7.89
7.89
7.89
7.89
7.88
7.52
7.52
7.52
7.50
7.50
7.50
7.49
7.49
7.48
7.47
7.47
7.47
7.46
7.45
7.45
7.44
7.43
7.43
7.26 CDCl3
7.25
7.24
7.23
7.23
7.22
7.21
7.20
4.13
3.72
2.01
1.00
3.07
1.00
3.05
3.04
f1 (ppm)

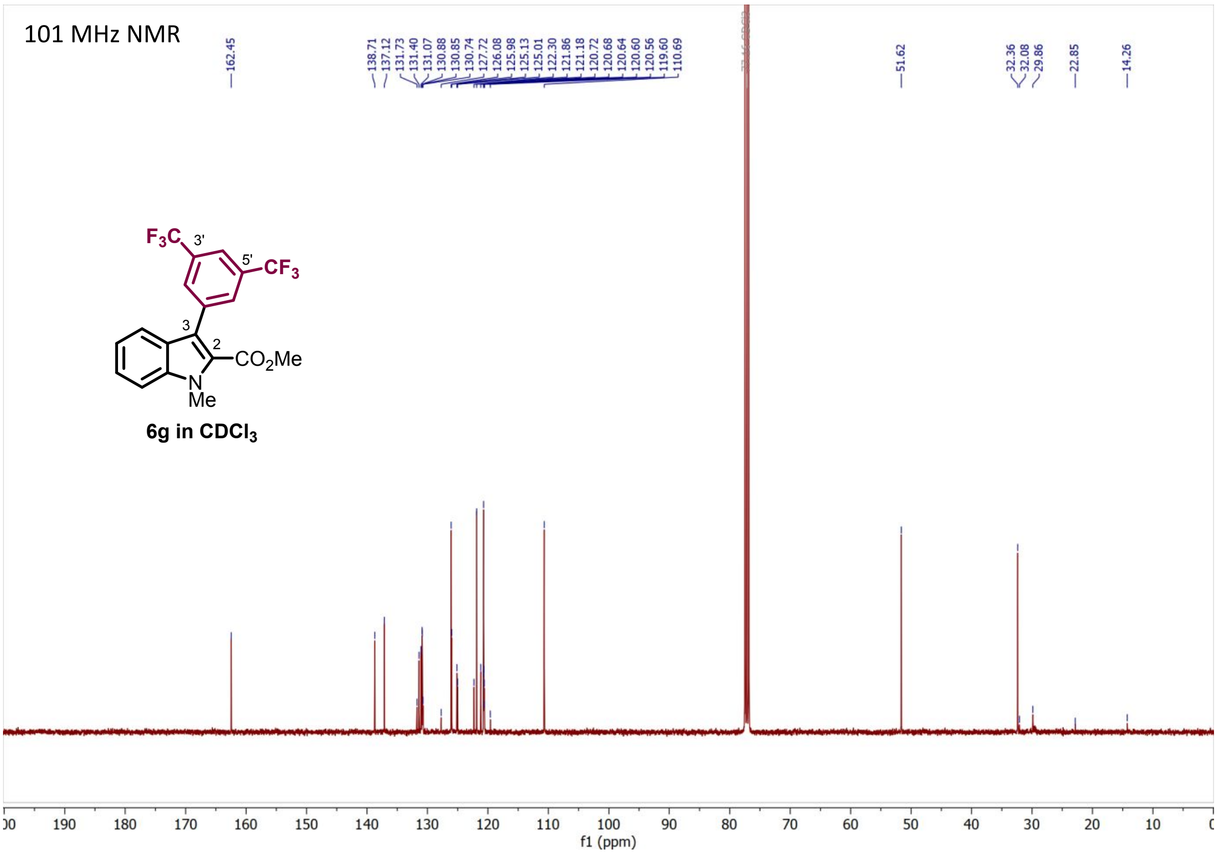
101 MHz NMR
F3C
3'
5'
CF3
3
2
CO2Me
N
Me
6g in CDCl3
162.45
138.71
137.12
131.73
131.40
131.07
130.88
130.85
130.74
127.72
126.08
125.98
125.13
125.01
122.30
121.86
121.18
120.72
120.68
120.64
120.60
120.56
119.60
110.69
51.62
32.36
32.08
29.86
22.85
14.26
f1 (ppm)

376 MHz NMR

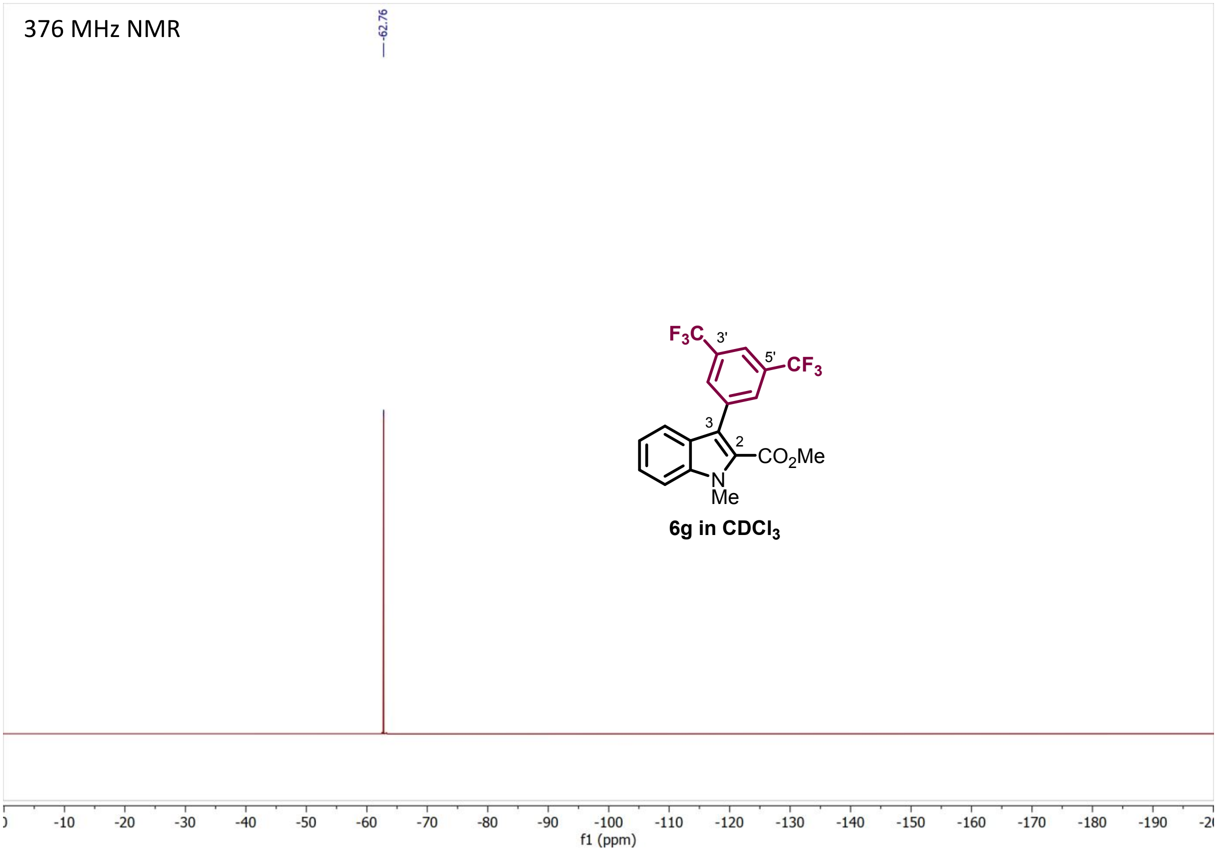

-62.76
F3C
3'
5'
CF3
3
2
CO2Me
N
Me
6g in CDCl3
0
-10
-20
-30
-40
-50
-60
-70
-80
-90
-100
-110
-120
-130
-140
-150
-160
-170
-180
-190
-200
f1 (ppm)

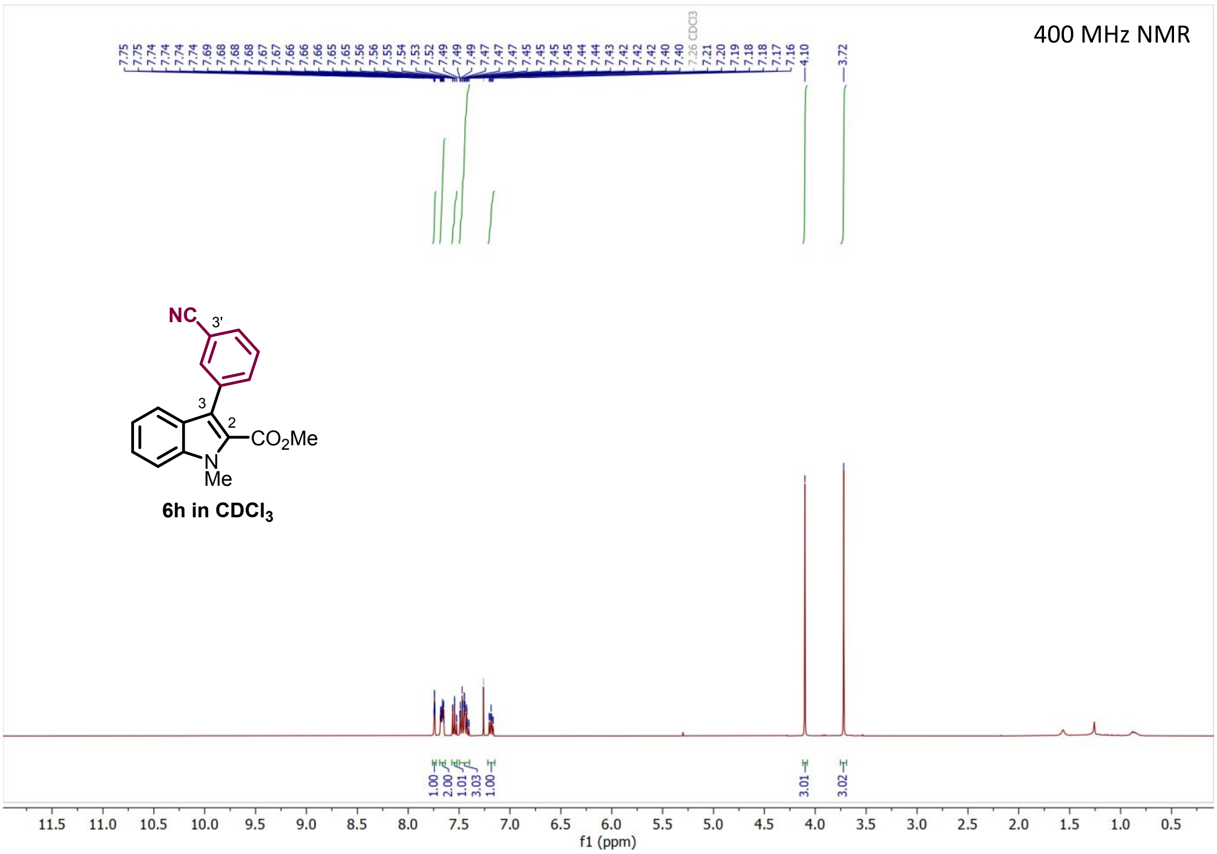

400 MHz NMR
7.75 7.75 7.74 7.74 7.74 7.69 7.68 7.68 7.67 7.67 7.66 7.66 7.66 7.65 7.65 7.56 7.56 7.55 7.54 7.53 7.52 7.49 7.49 7.49 7.49 7.47 7.47 7.45 7.45 7.45 7.45 7.44 7.44 7.43 7.42 7.42 7.42 7.40 7.40 7.26 CDCl3 7.21 7.20 7.19 7.18 7.18 7.17 7.16 4.10 3.72
NC
3'
3
2
CO2Me
N
Me
6h in CDCl3
1.00 2.00 1.01 3.03 1.00 3.01 3.02
11.5 11.0 10.5 10.0 9.5 9.0 8.5 8.0 7.5 7.0 6.5 6.0 5.5 5.0 4.5 4.0 3.5 3.0 2.5 2.0 1.5 1.0 0.5
f1 (ppm)

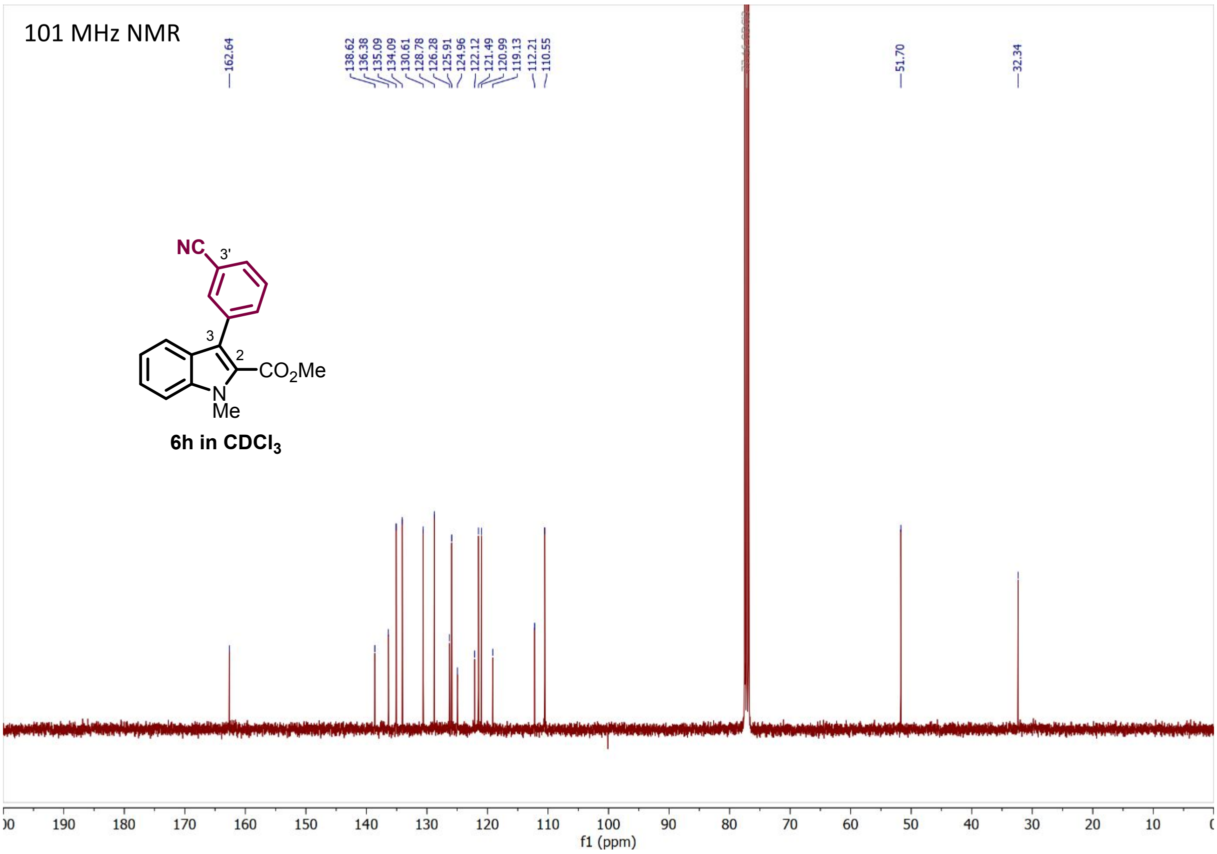

101 MHz NMR
162.64
138.62 136.38 135.09 134.09 130.61 128.78 126.28 125.91 124.96 122.12 121.49 120.99 119.13 112.21 110.55
51.70
32.34
NC
3'
3
2
CO2Me
N
Me
6h in CDCl3
190 180 170 160 150 140 130 120 110 100 90 80 70 60 50 40 30 20 10
f1 (ppm)

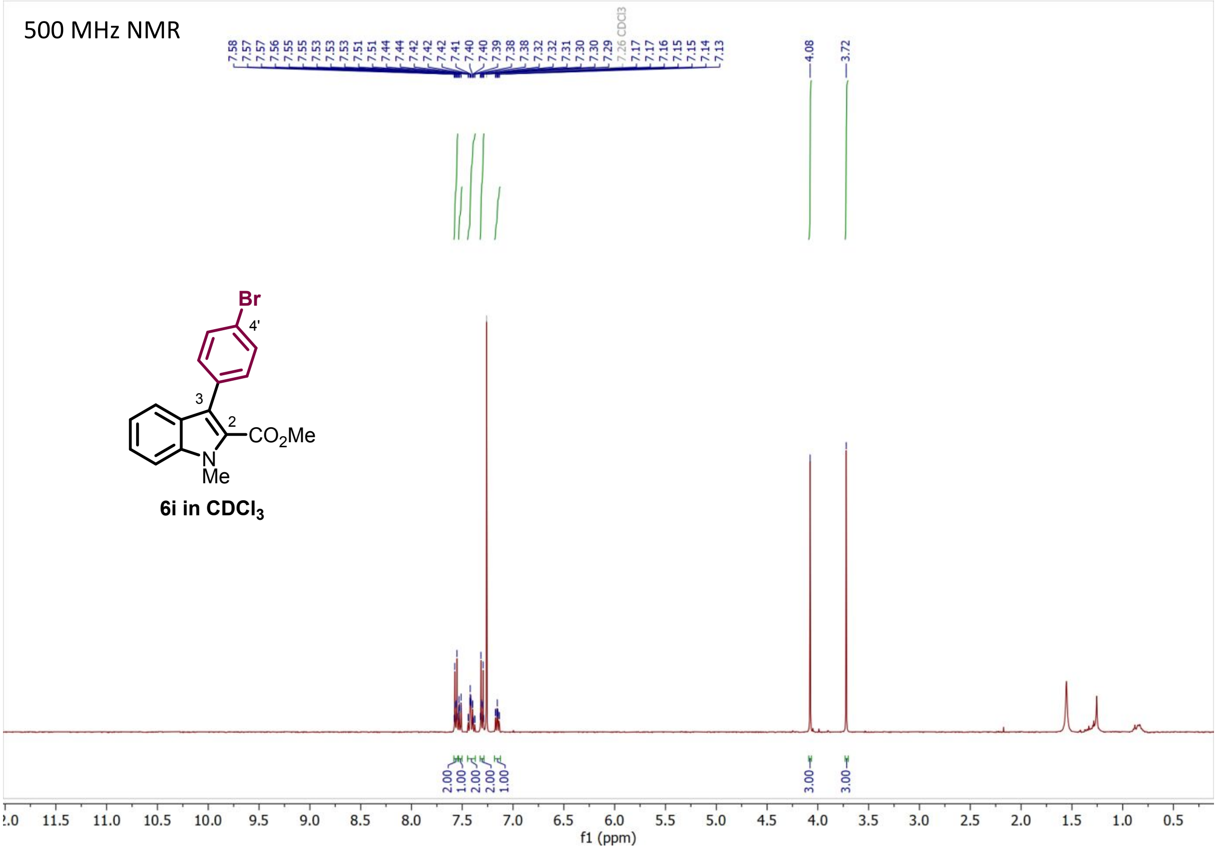
500 MHz NMR
7.58 7.57 7.56 7.55 7.55 7.53 7.53 7.53 7.51 7.51 7.44 7.42 7.42 7.42 7.41 7.40 7.40 7.39 7.38 7.38 7.32 7.32 7.31 7.30 7.30 7.29 7.26 CDCl3 7.17 7.17 7.16 7.15 7.15 7.14 7.13
4.08
3.72
Br
4'
3
2
CO2Me
N
Me
6i in CDCl3
2.00 1.00 2.00 2.00 1.00
3.00
3.00
f1 (ppm)

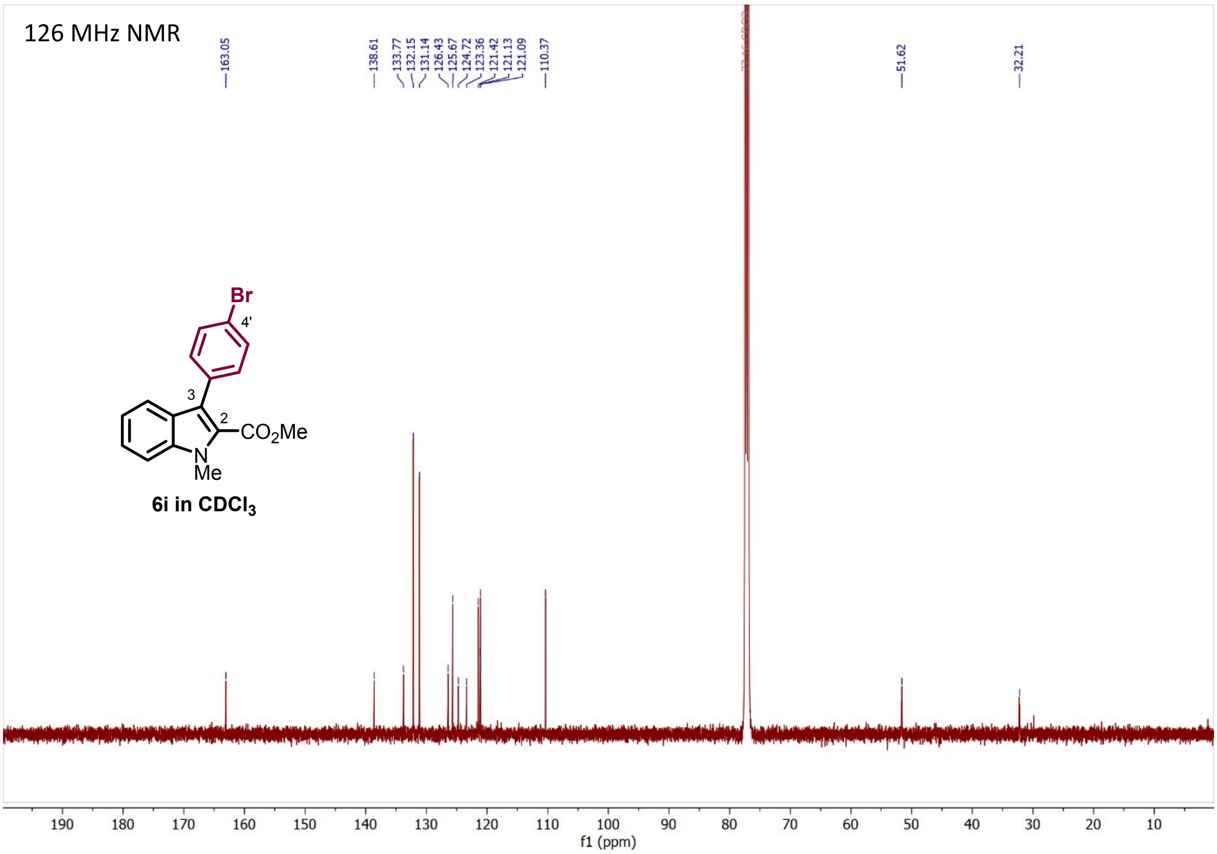
126 MHz NMR
163.05
138.61 133.77 132.15 131.14 126.43 125.67 124.72 123.36 121.42 121.13 121.09
110.37
51.62
32.21
Br
4'
3
2
CO2Me
N
Me
6i in CDCl3
f1 (ppm)

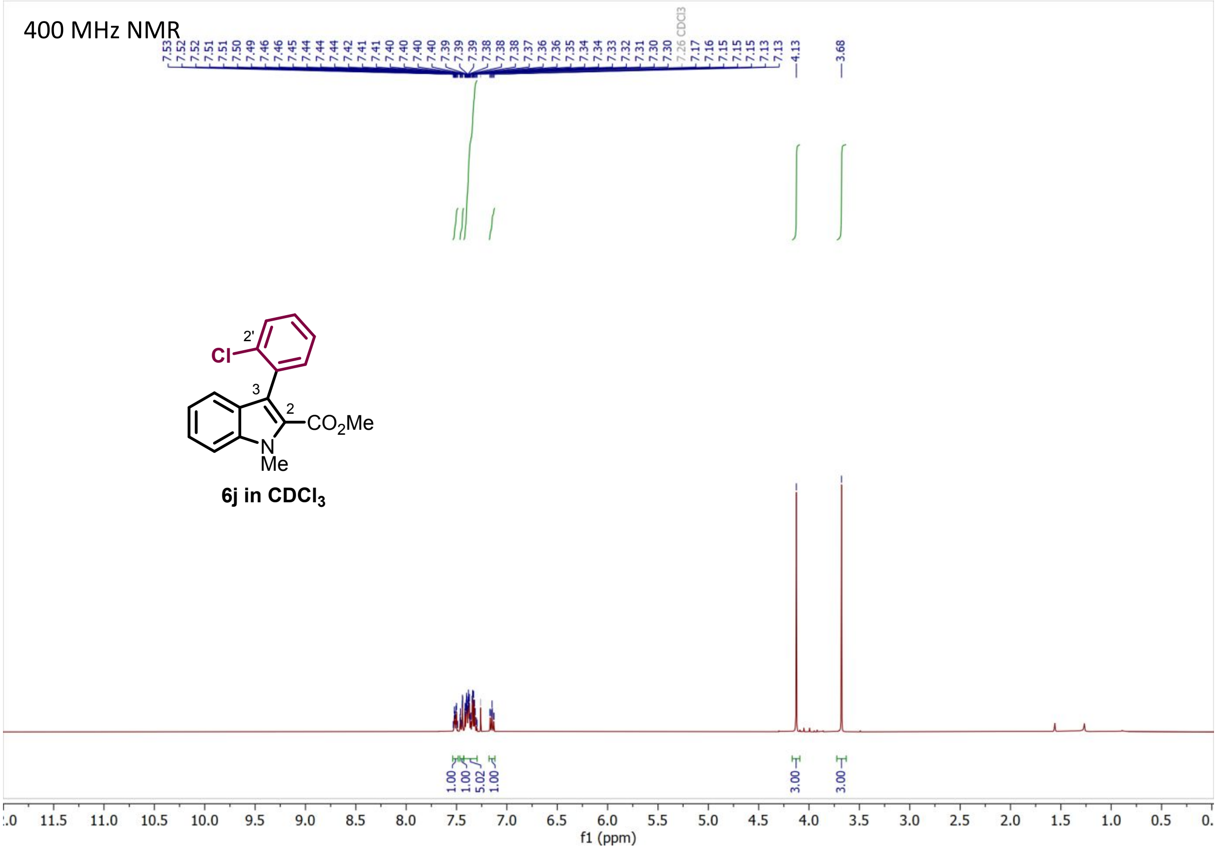
400 MHz NMR
6j in CDCl3
f1 (ppm)

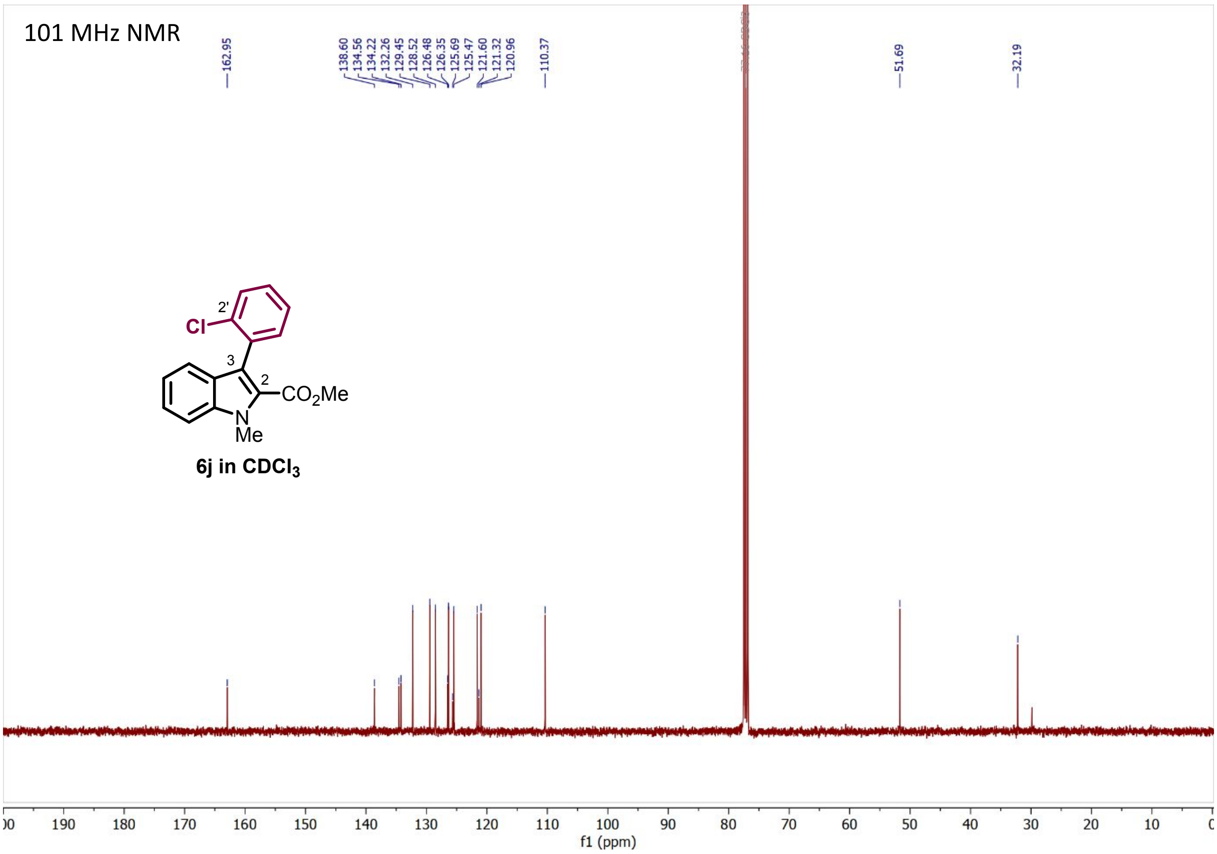
101 MHz NMR
6j in CDCl3
f1 (ppm)

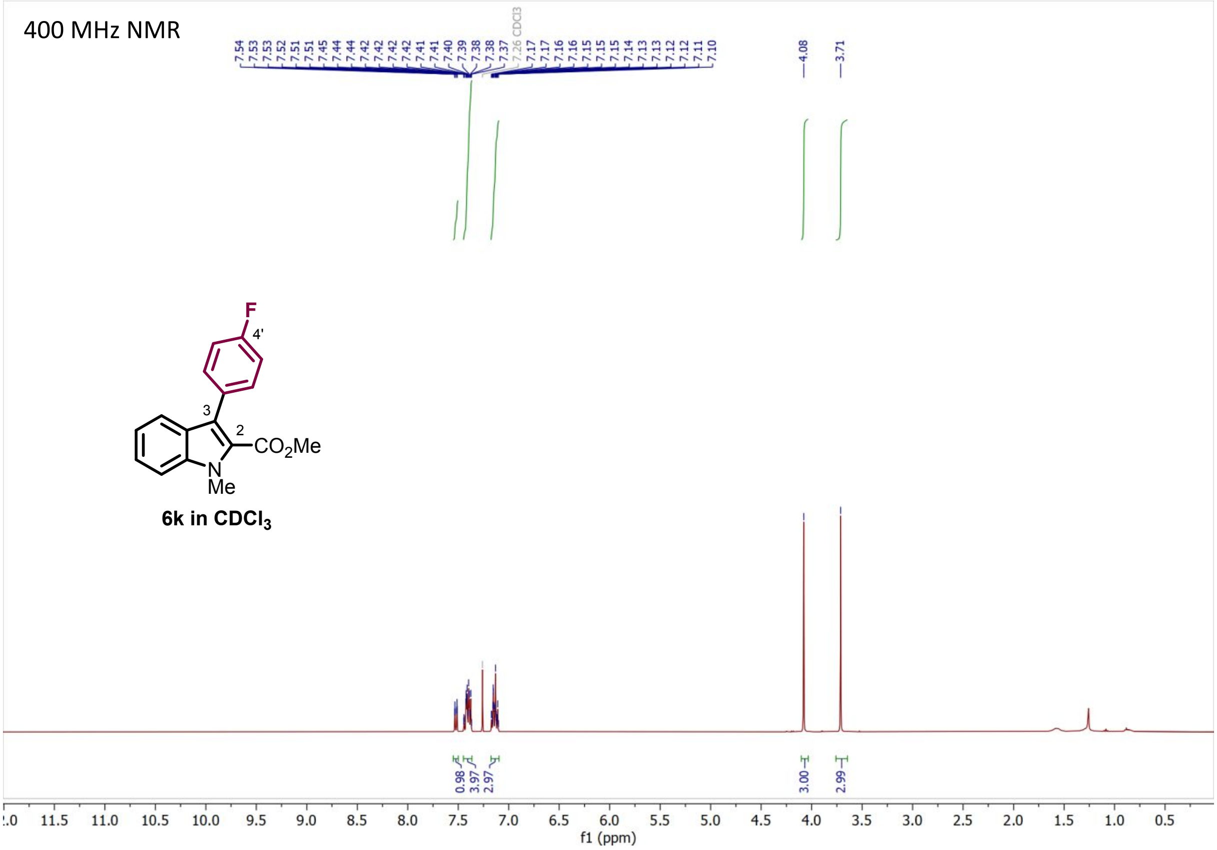

6k in $CDCl_3$

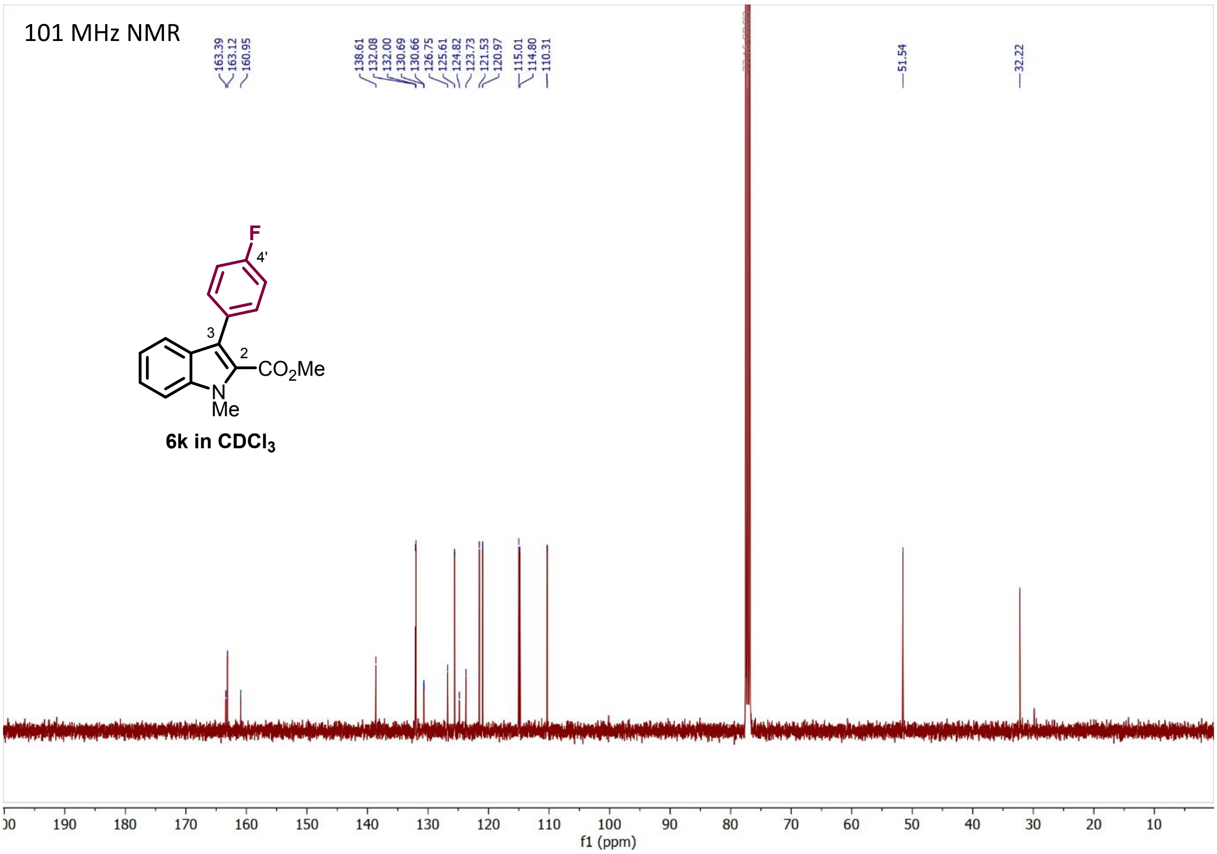

6k in $CDCl_3$

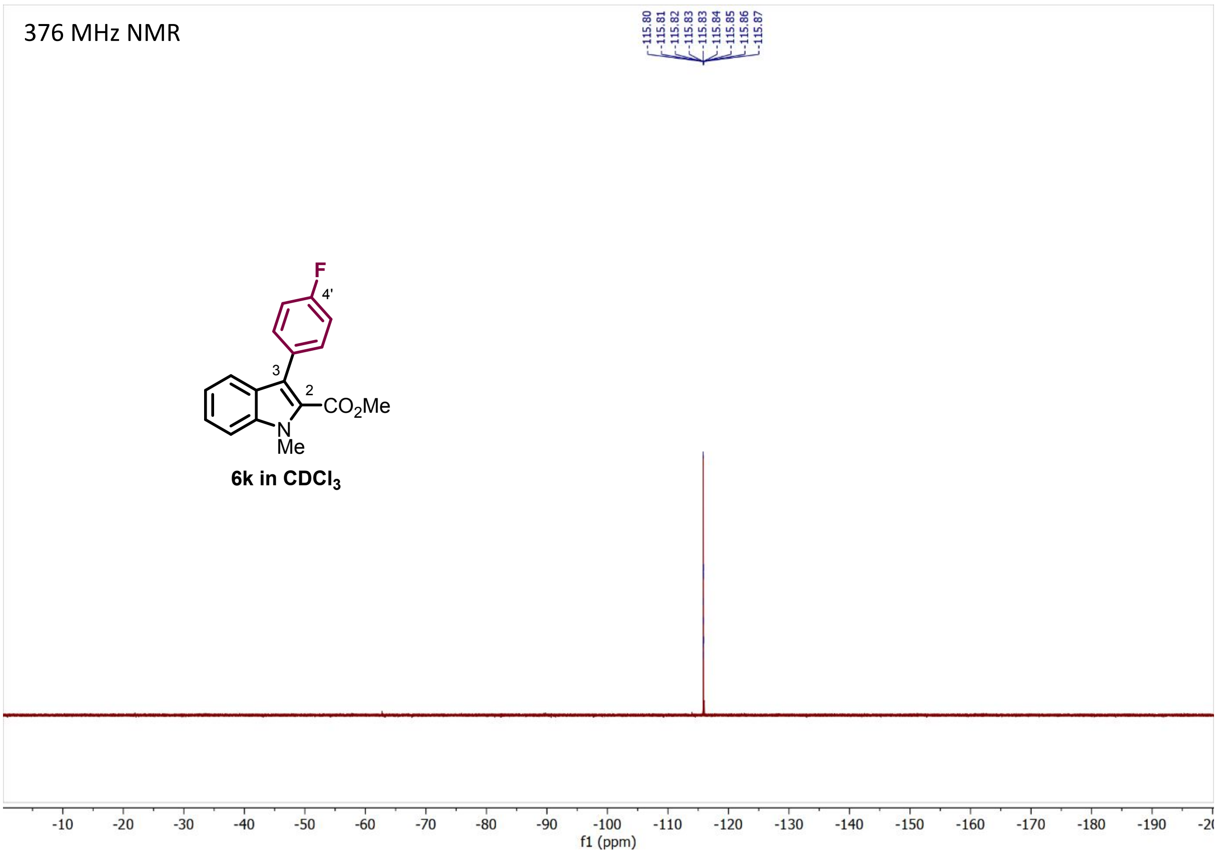

376 MHz NMR
-115.80
-115.81
-115.82
-115.83
-115.83
-115.84
-115.85
-115.86
-115.87
F
4'
3
2
CO2Me
N
Me
6k in CDCl3
-10
-20
-30
-40
-50
-60
-70
-80
-90
-100
-110
-120
-130
-140
-150
-160
-170
-180
-190
f1 (ppm)

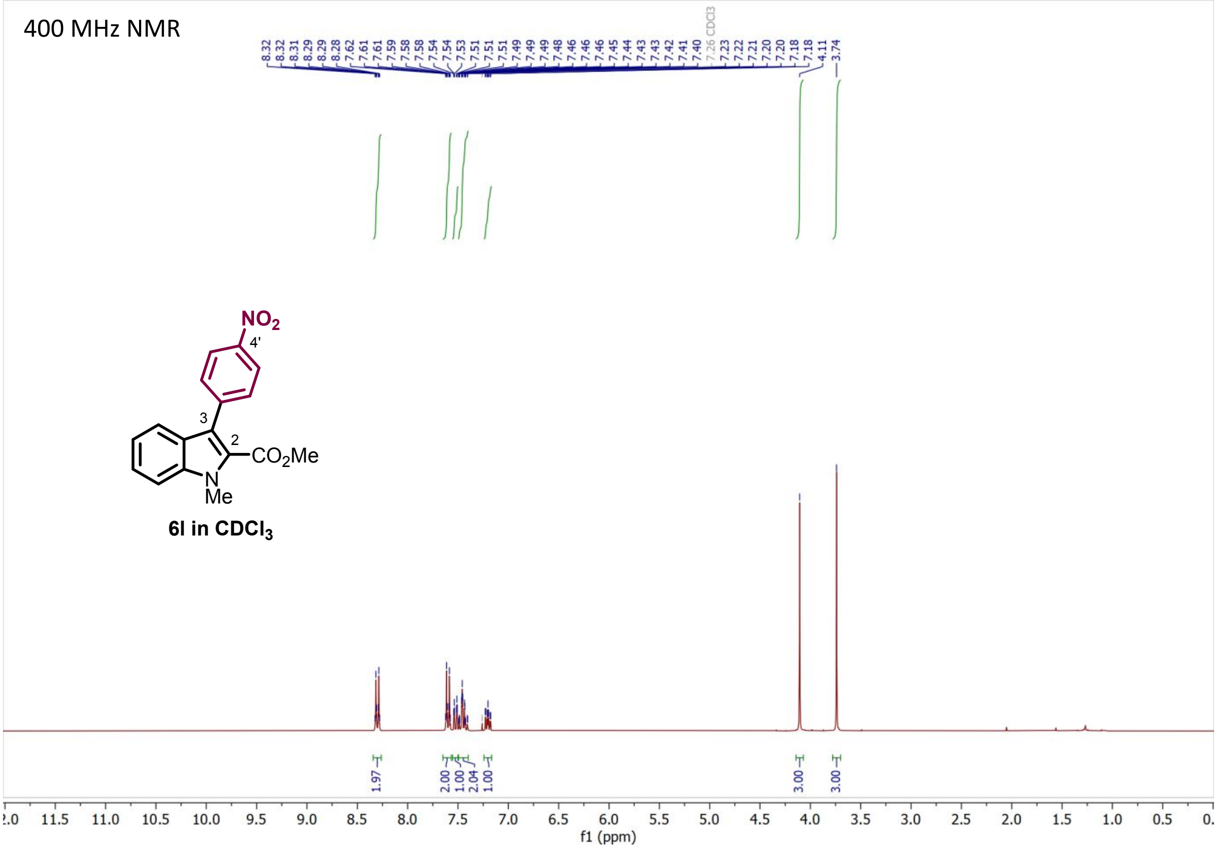
400 MHz NMR
6l in CDCl3
NO2
4'
3
2
CO2Me
N
Me
f1 (ppm)

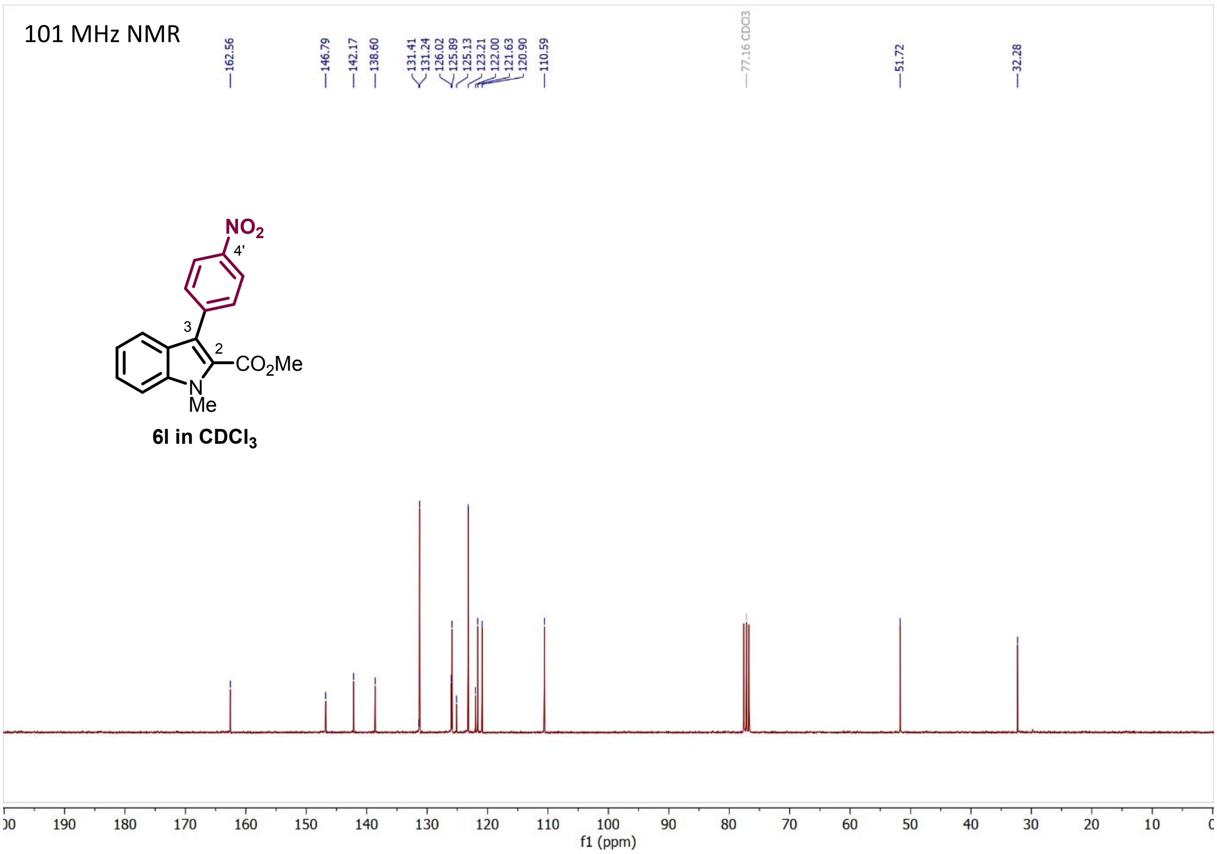
101 MHz NMR
162.56
146.79
142.17
138.60
131.41
131.24
126.02
125.89
125.13
123.21
122.00
121.63
120.90
110.59
77.16 CDCl3
51.72
32.28
6l in CDCl3
NO2
4'
3
2
CO2Me
N
Me
f1 (ppm)

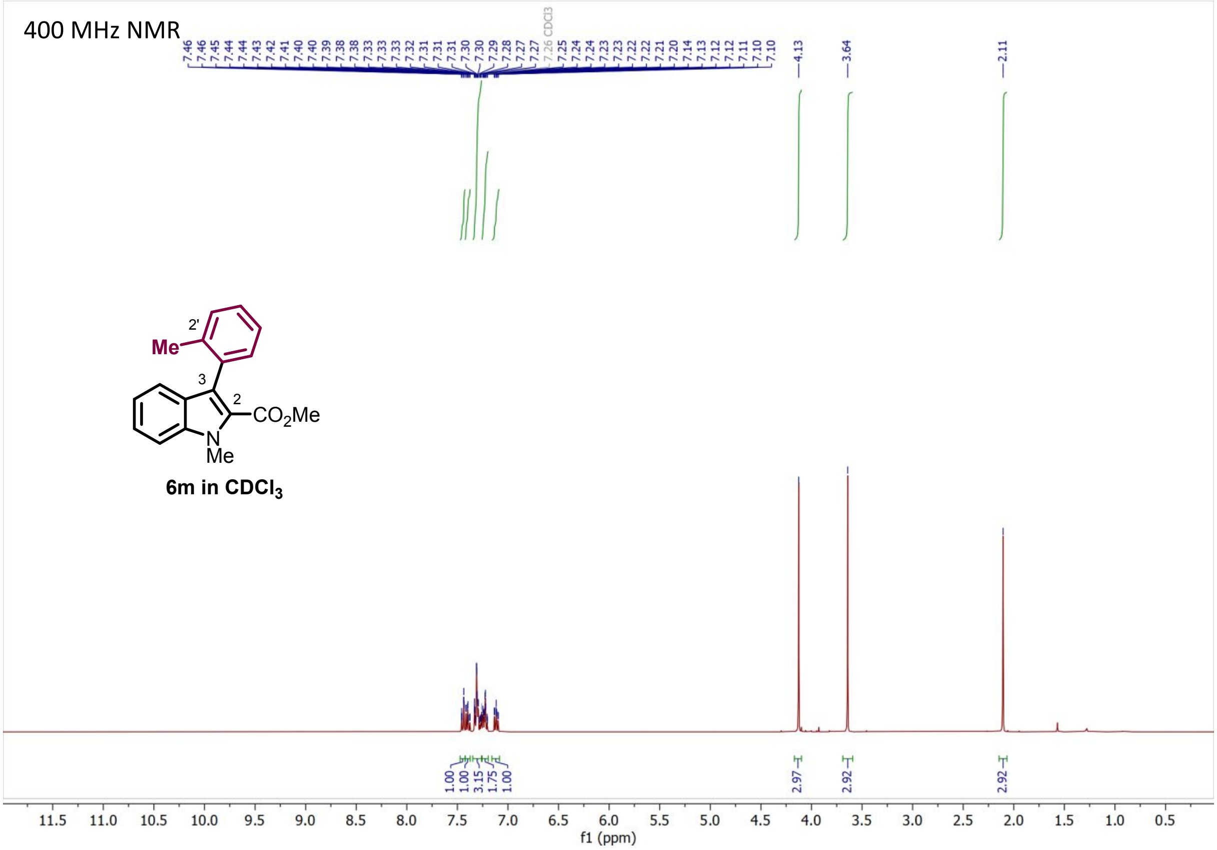
400 MHz NMR
6m in CDCl3
f1 (ppm)

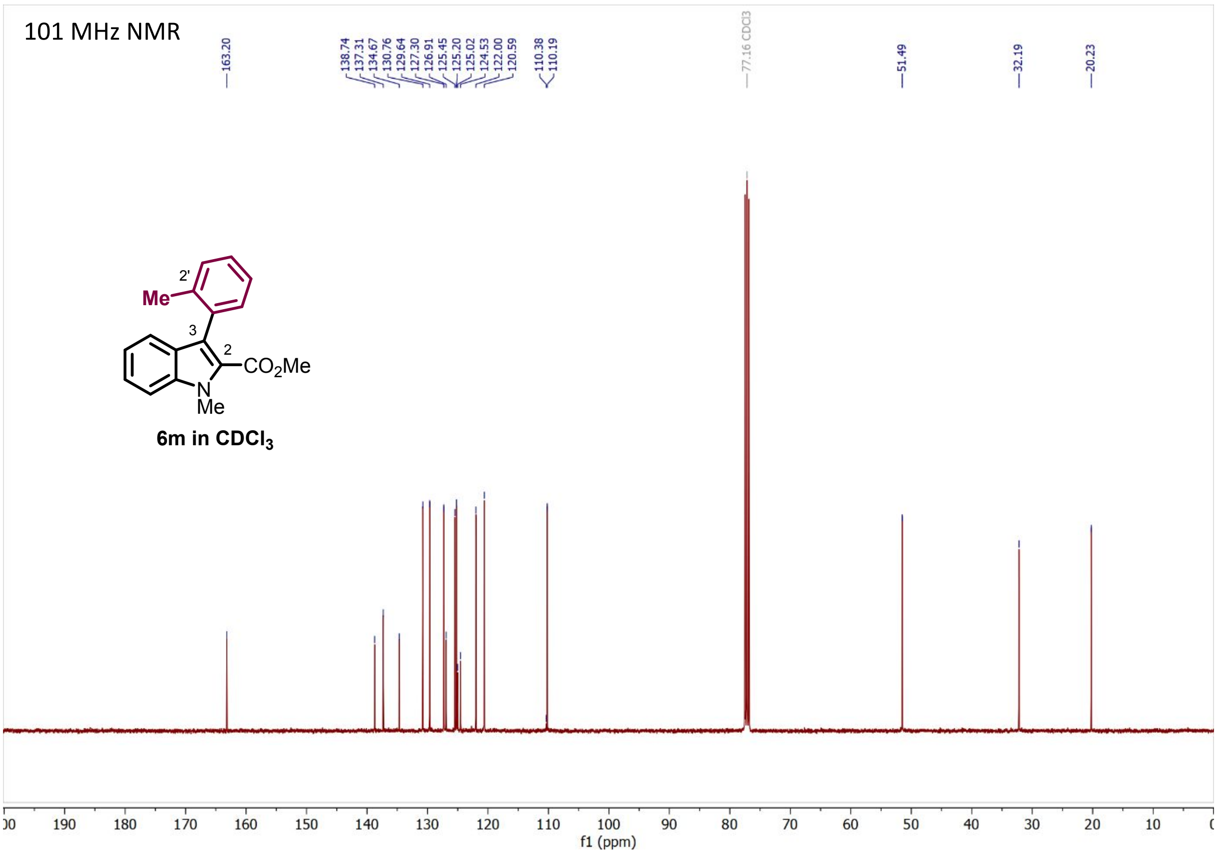
101 MHz NMR
6m in CDCl3
f1 (ppm)

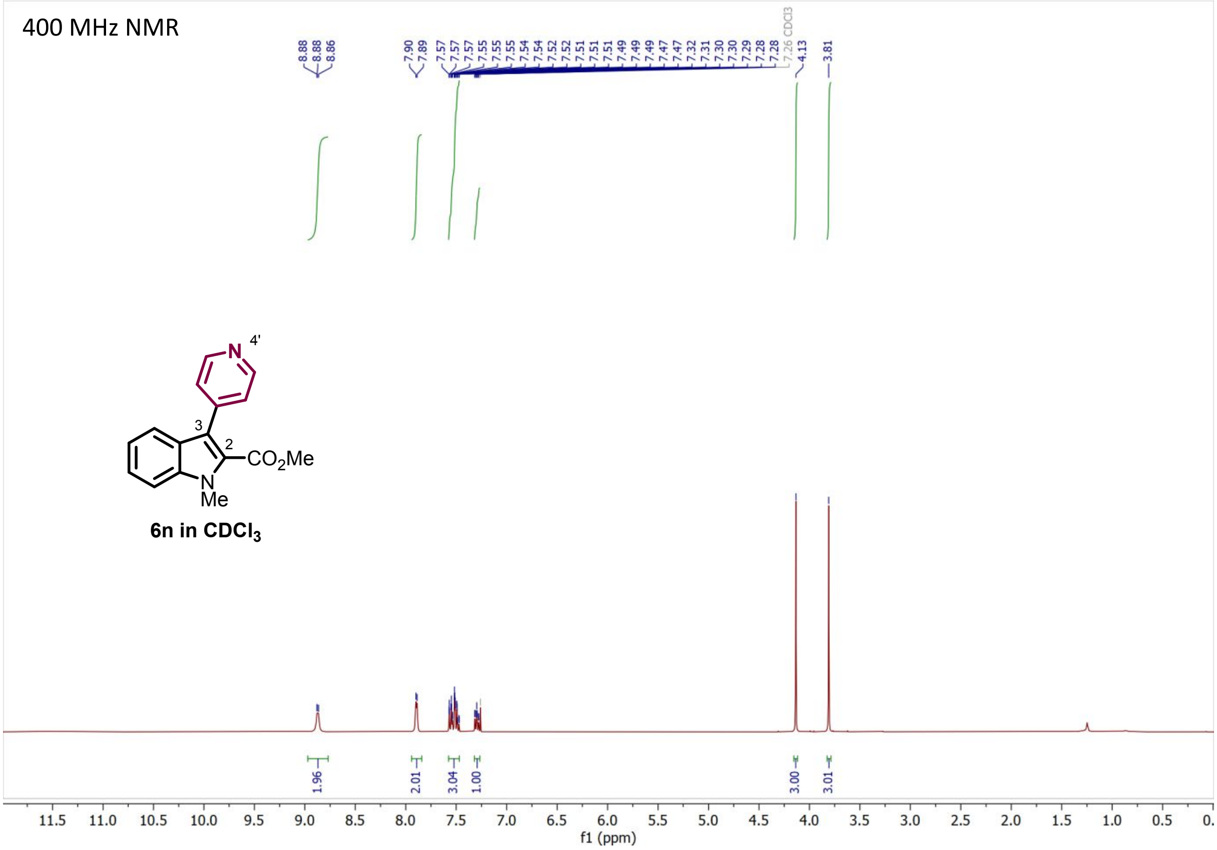
400 MHz NMR
6n in CDCl3
f1 (ppm)

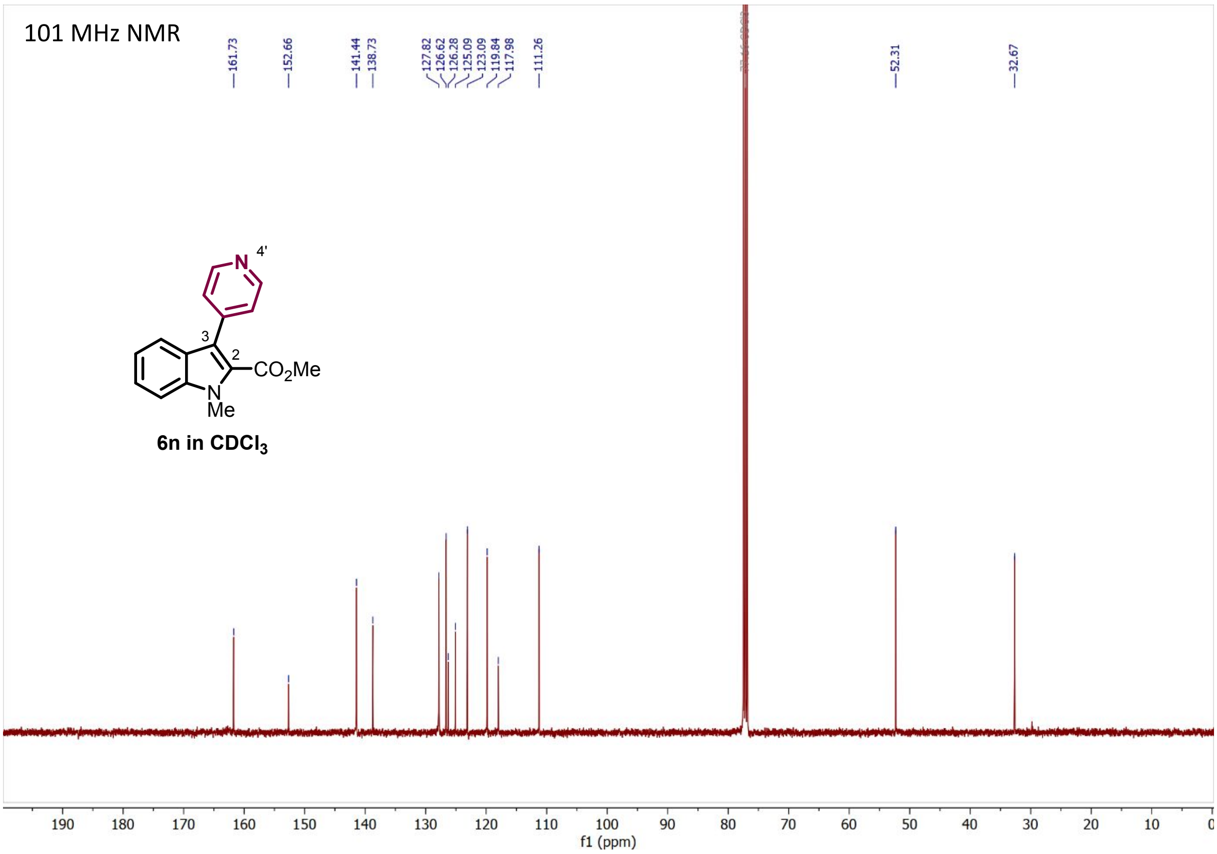
101 MHz NMR
6n in CDCl3
f1 (ppm)

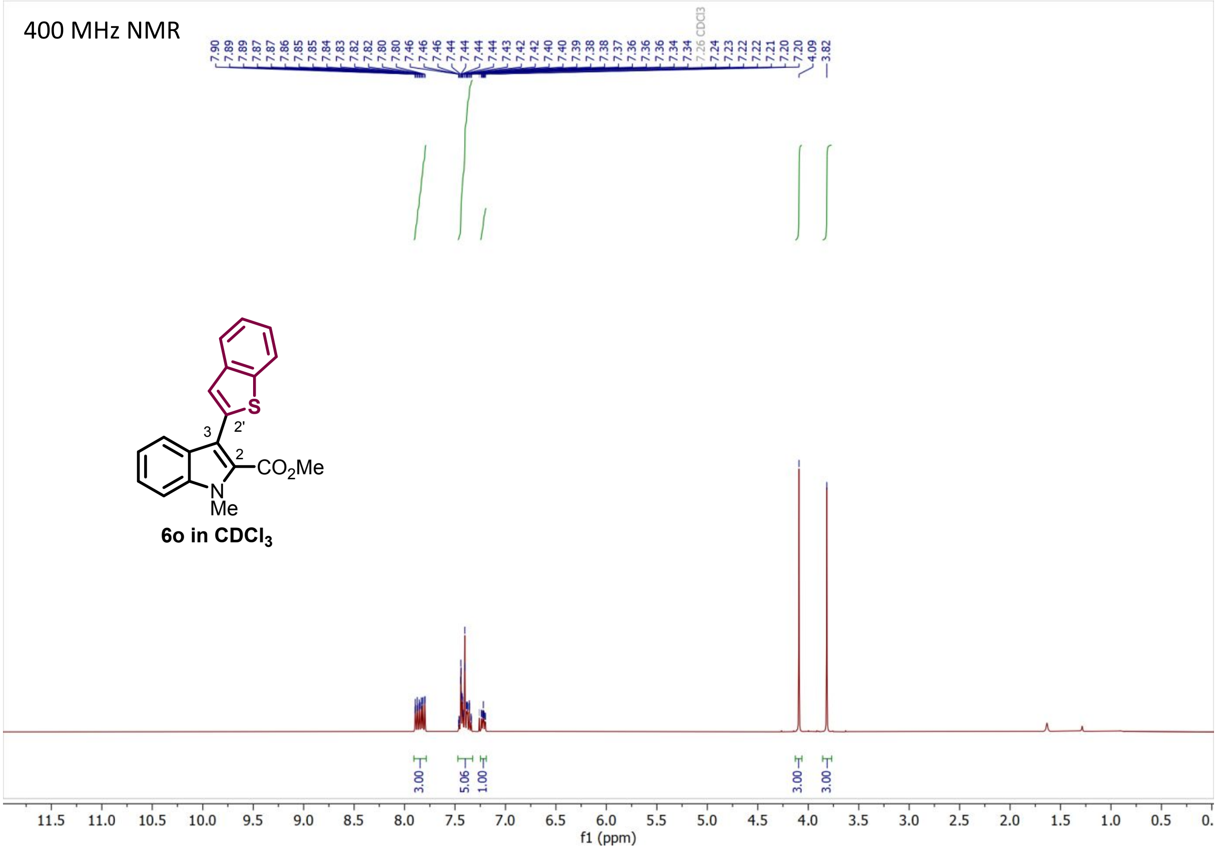

400 MHz NMR
7.90 7.89 7.89 7.87 7.87 7.86 7.85 7.85 7.84 7.83 7.82 7.82 7.80 7.80 7.46 7.46 7.46 7.44 7.44 7.44 7.44 7.43 7.42 7.42 7.40 7.40 7.39 7.38 7.38 7.37 7.36 7.36 7.36 7.34 7.34 7.26 CDCl3 7.24 7.23 7.23 7.22 7.22 7.21 7.20 7.20 4.09 3.82
3.00 5.06 1.00 3.00 3.00
2'
3
2
CO2Me
N
Me
S
6o in CDCl3
f1 (ppm)

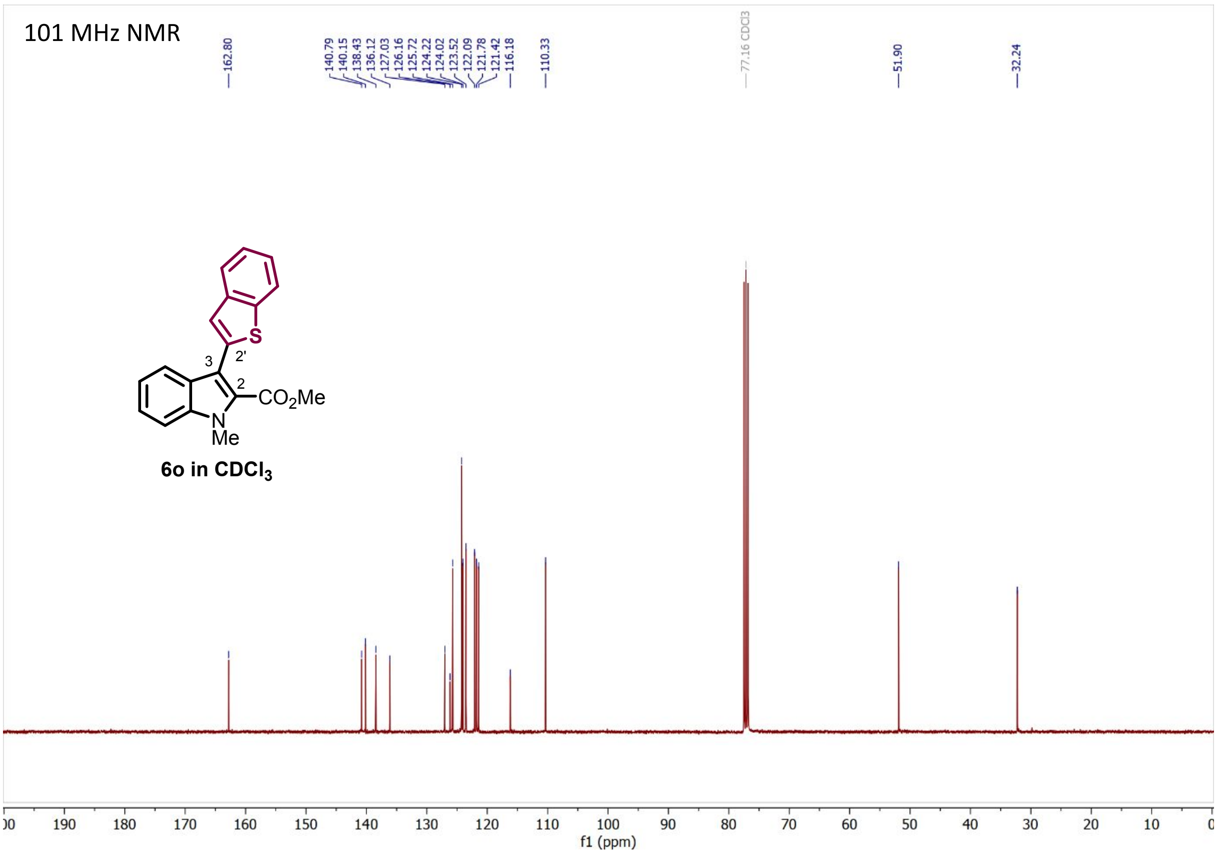

101 MHz NMR
162.80 140.79 140.15 138.43 136.12 127.03 126.16 125.72 124.22 124.02 123.52 122.09 121.78 121.42 116.18 110.33 77.16 CDCl3 51.90 32.24
2'
3
2
CO2Me
N
Me
S
6o in CDCl3
f1 (ppm)

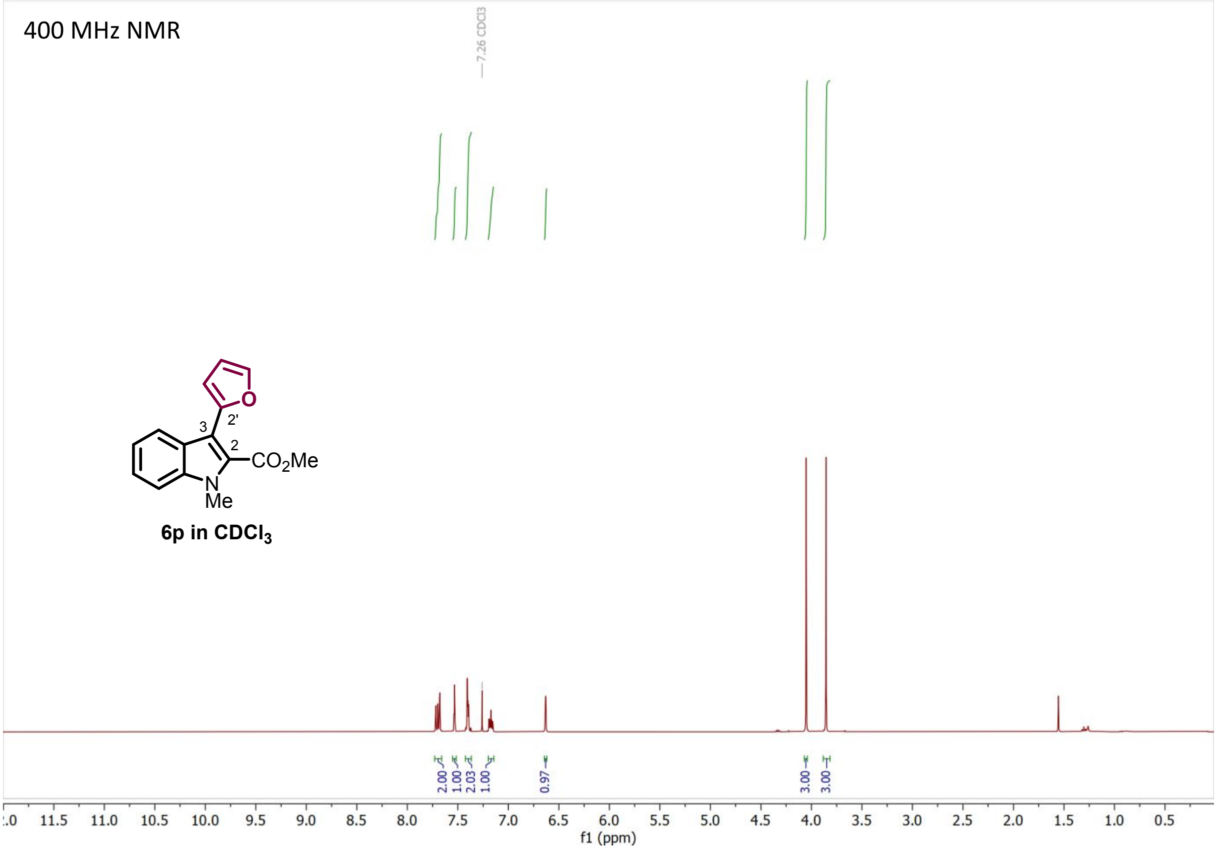
400 MHz NMR
7.26 CDCl3
6p in CDCl3
2.00
1.00
2.03
1.00
0.97
3.00
3.00
f1 (ppm)

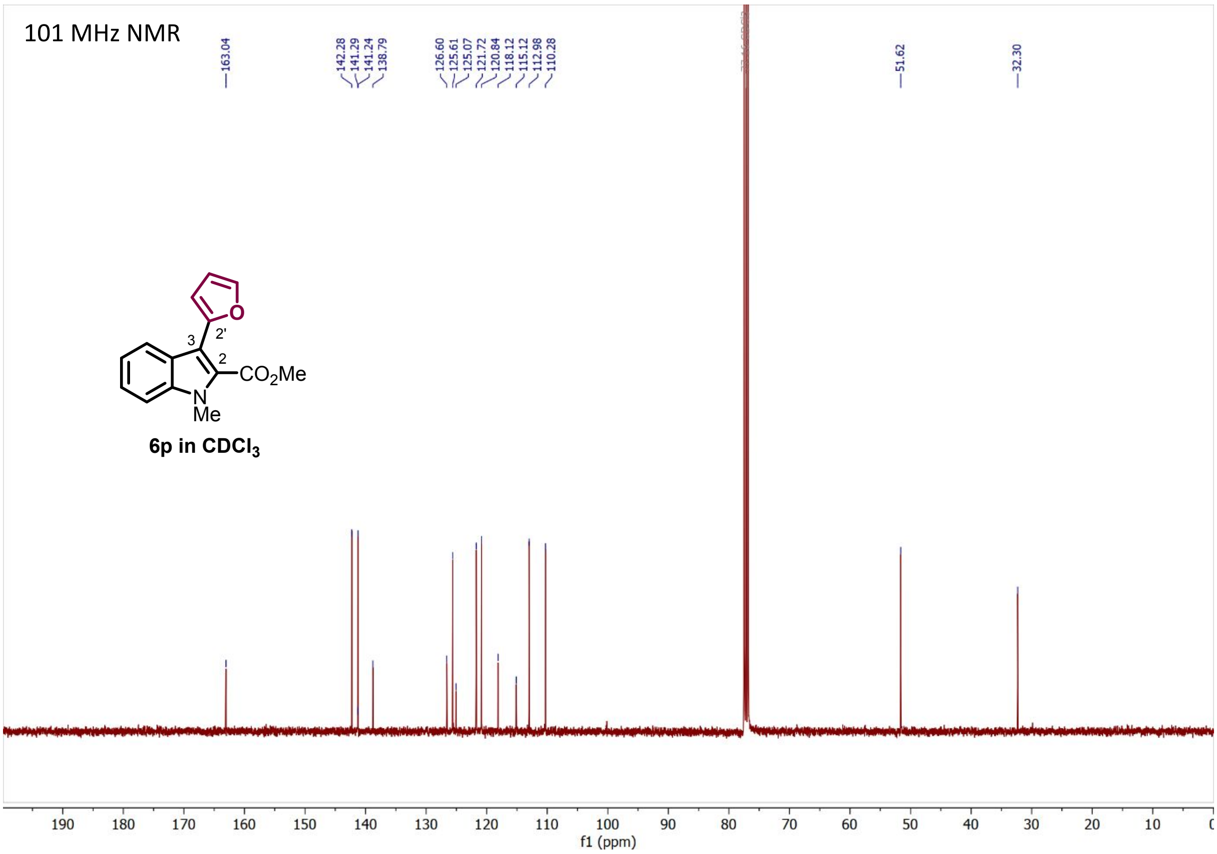
101 MHz NMR
163.04
142.28
141.29
141.24
138.79
126.60
125.61
125.07
121.72
120.84
118.12
115.12
112.98
110.28
51.62
32.30
6p in CDCl3
f1 (ppm)

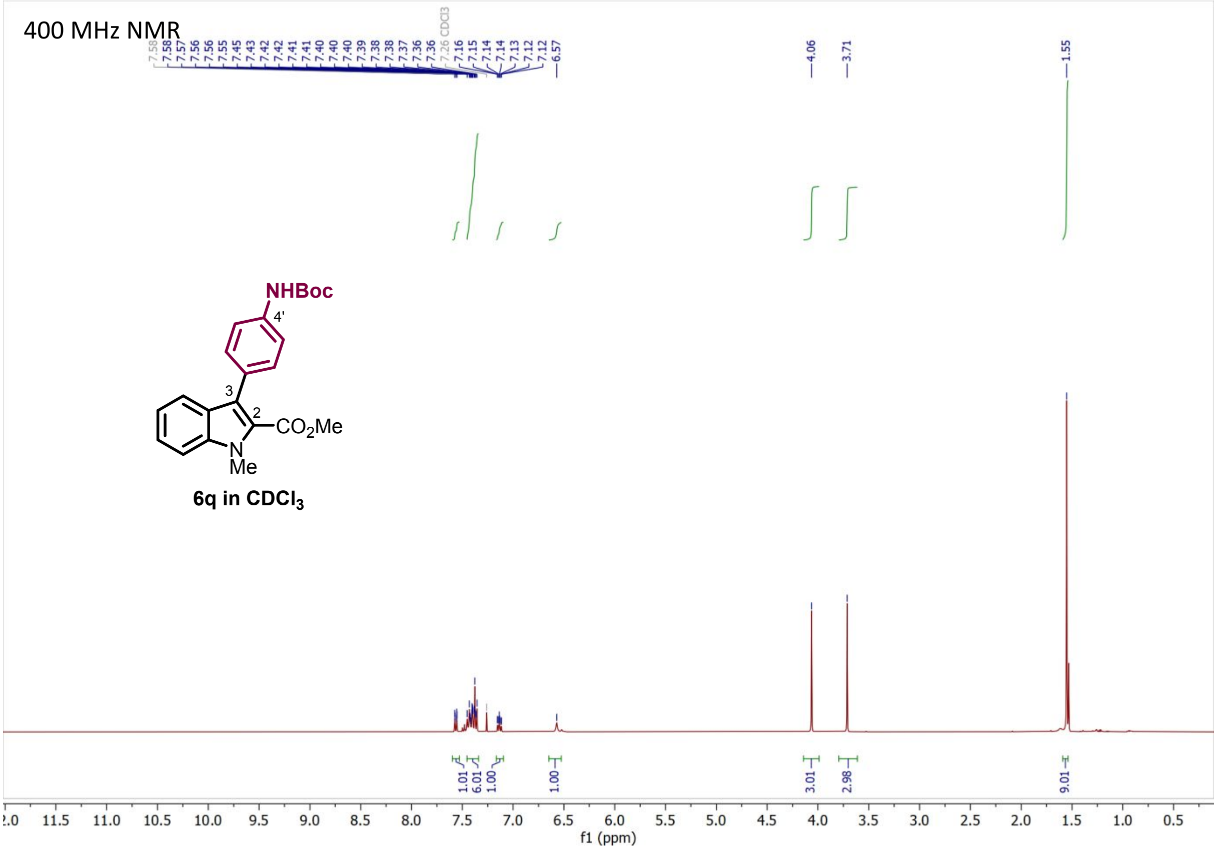
400 MHz NMR
NHBoc
4'
3
2
CO2Me
N
Me
6q in CDCl3
f1 (ppm)

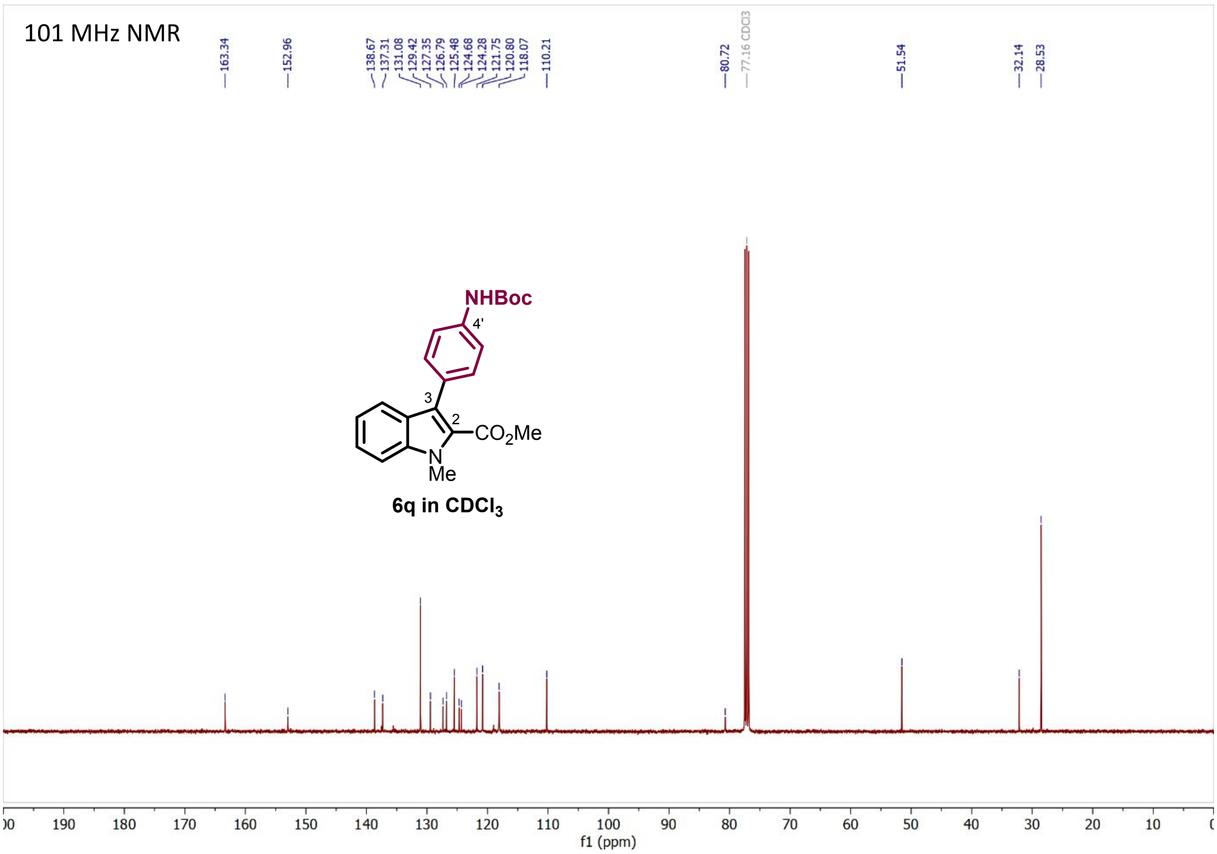
101 MHz NMR
NHBoc
4'
3
2
CO2Me
N
Me
6q in CDCl3
f1 (ppm)

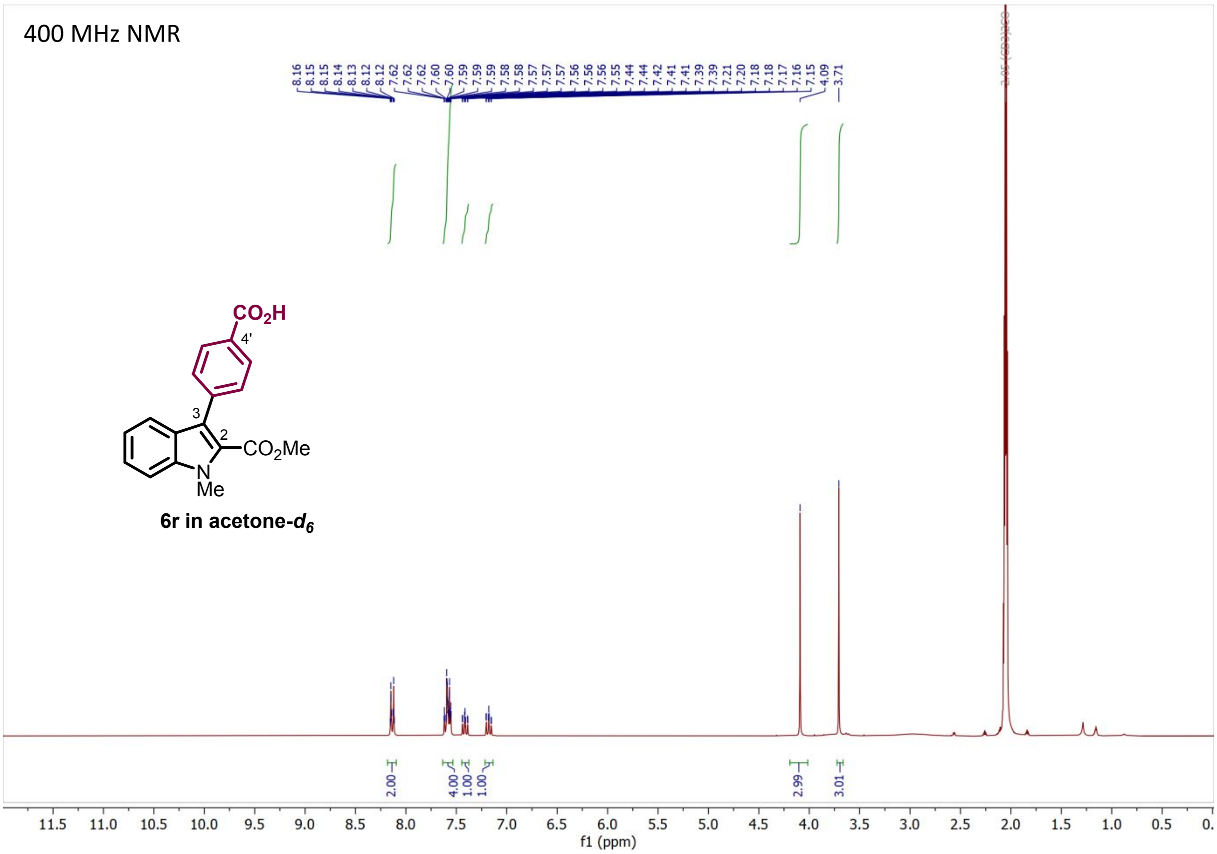

400 MHz NMR
8.16 8.15 8.15 8.14 8.13 8.12 8.12 7.62 7.62 7.62 7.60 7.60 7.59 7.59 7.59 7.58 7.58 7.57 7.57 7.57 7.56 7.56 7.55 7.44 7.44 7.42 7.41 7.41 7.39 7.39 7.21 7.20 7.18 7.18 7.17 7.16 7.15 4.09 3.71
CO2H
4'
3
2
CO2Me
N
Me
6r in acetone-d6
2.00
4.00
1.00
1.00
2.99
3.01
11.5 11.0 10.5 10.0 9.5 9.0 8.5 8.0 7.5 7.0 6.5 6.0 5.5 5.0 4.5 4.0 3.5 3.0 2.5 2.0 1.5 1.0 0.5
f1 (ppm)

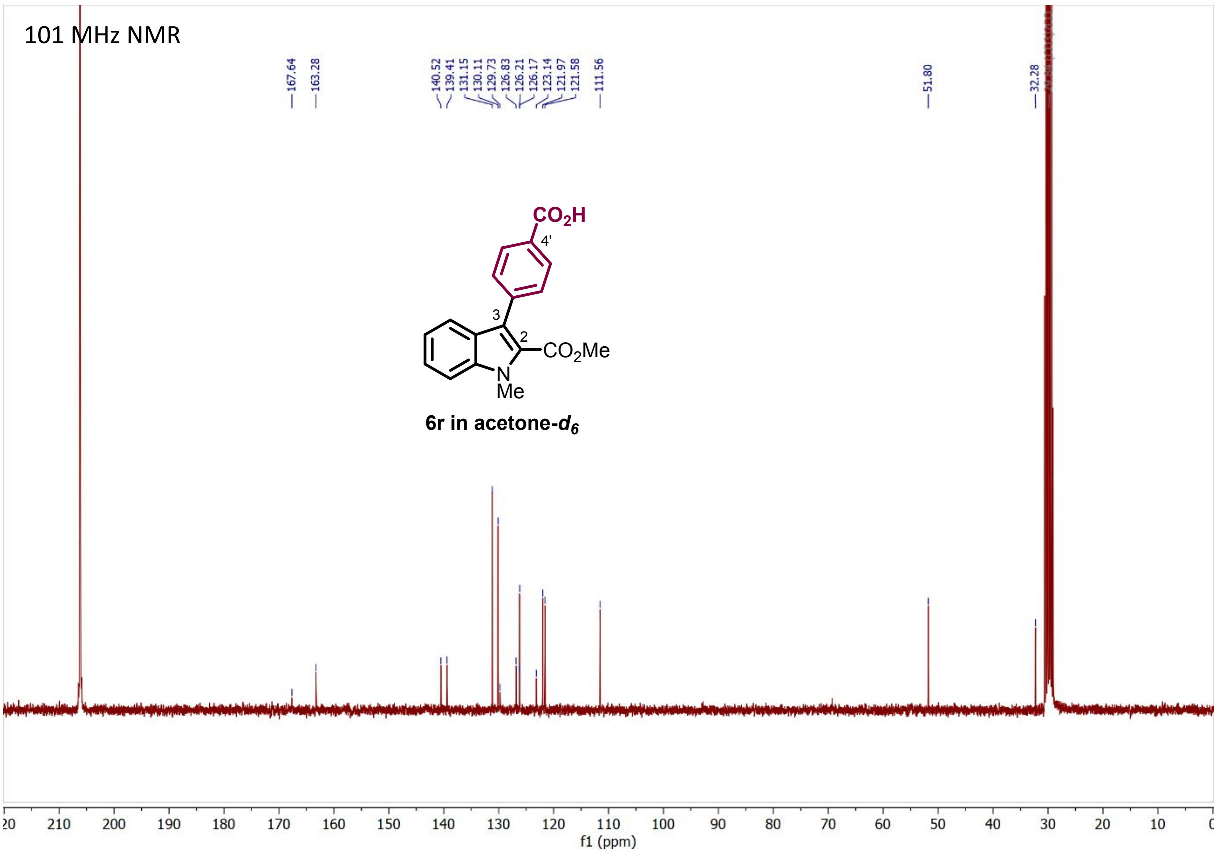

101 MHz NMR
167.64 163.28
140.52 139.41 131.15 130.11 129.73 126.83 126.21 126.17 123.14 121.97 121.58
111.56
51.80
32.28
CO2H
4'
3
2
CO2Me
N
Me
6r in acetone-d6
210 200 190 180 170 160 150 140 130 120 110 100 90 80 70 60 50 40 30 20 10
f1 (ppm)

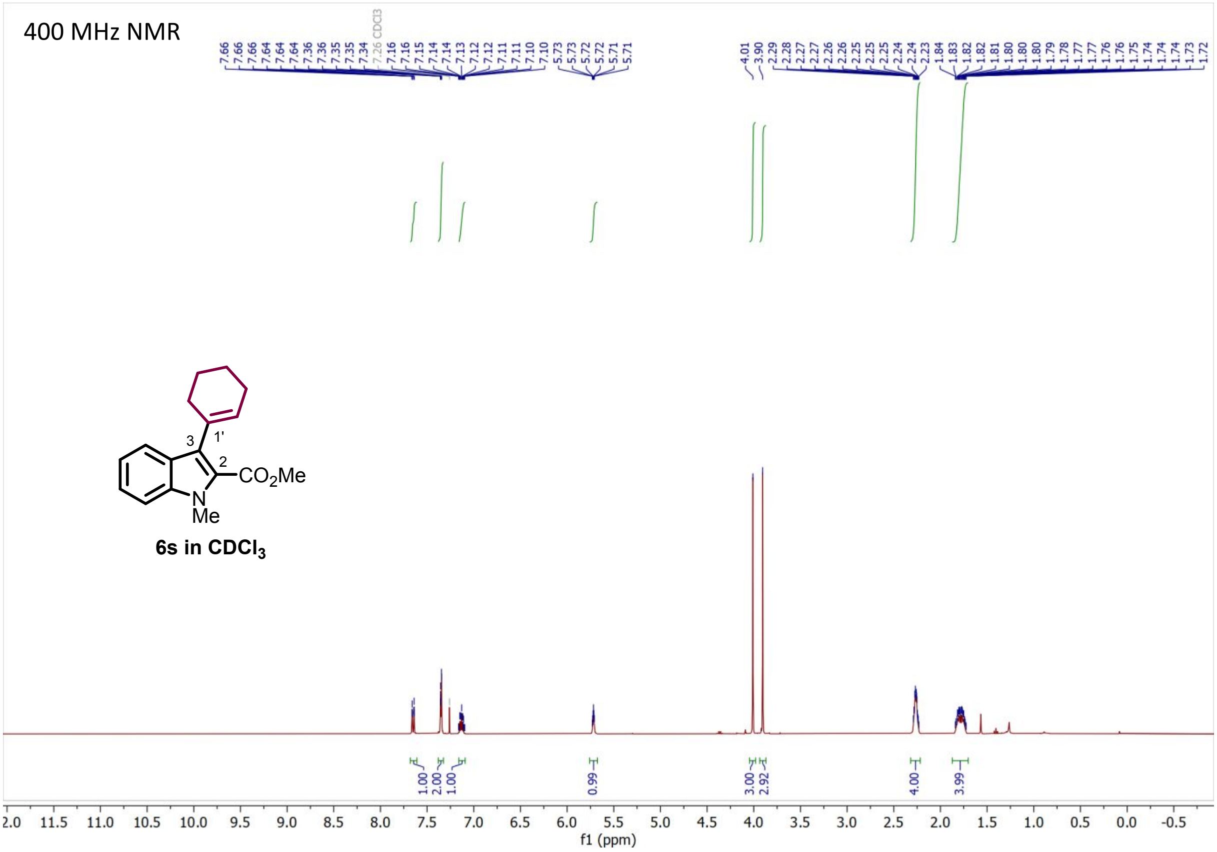
400 MHz NMR
6s in CDCl3
f1 (ppm)

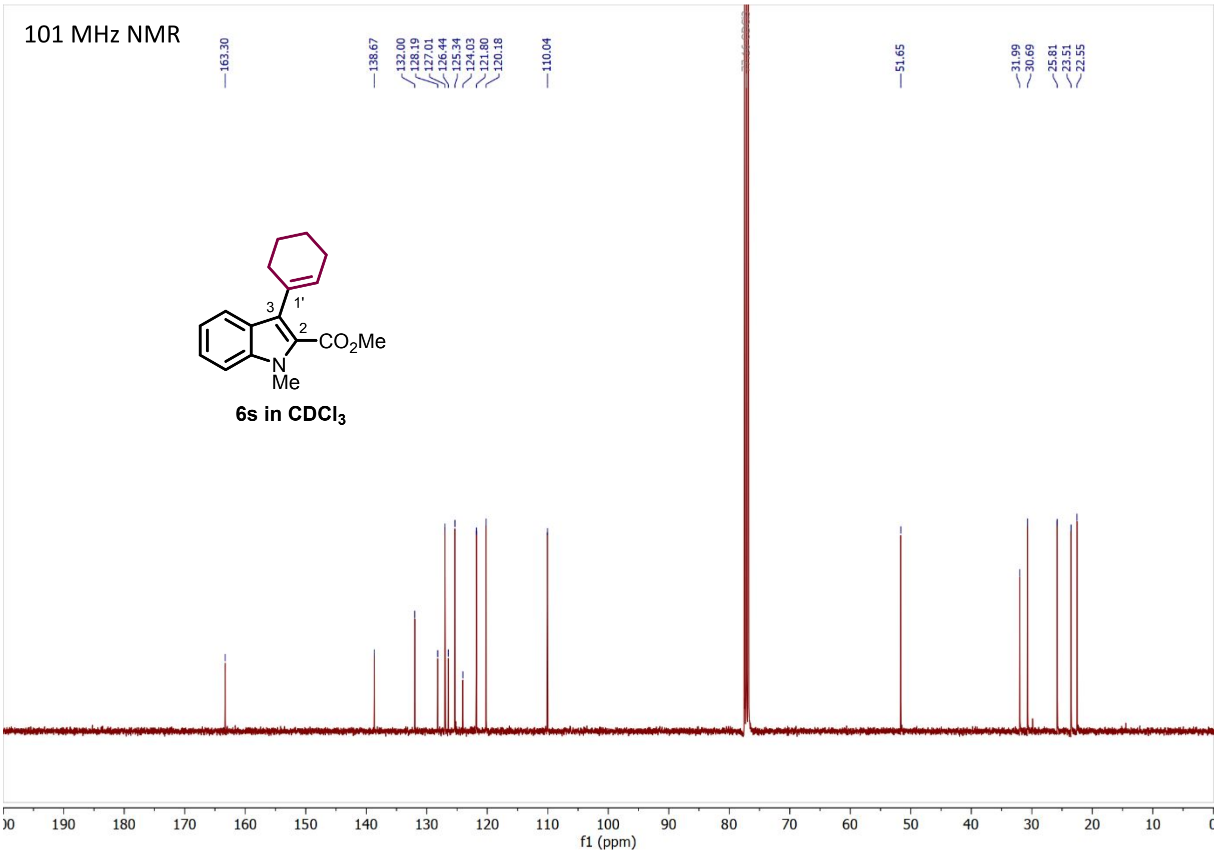
101 MHz NMR
6s in CDCl3
f1 (ppm)

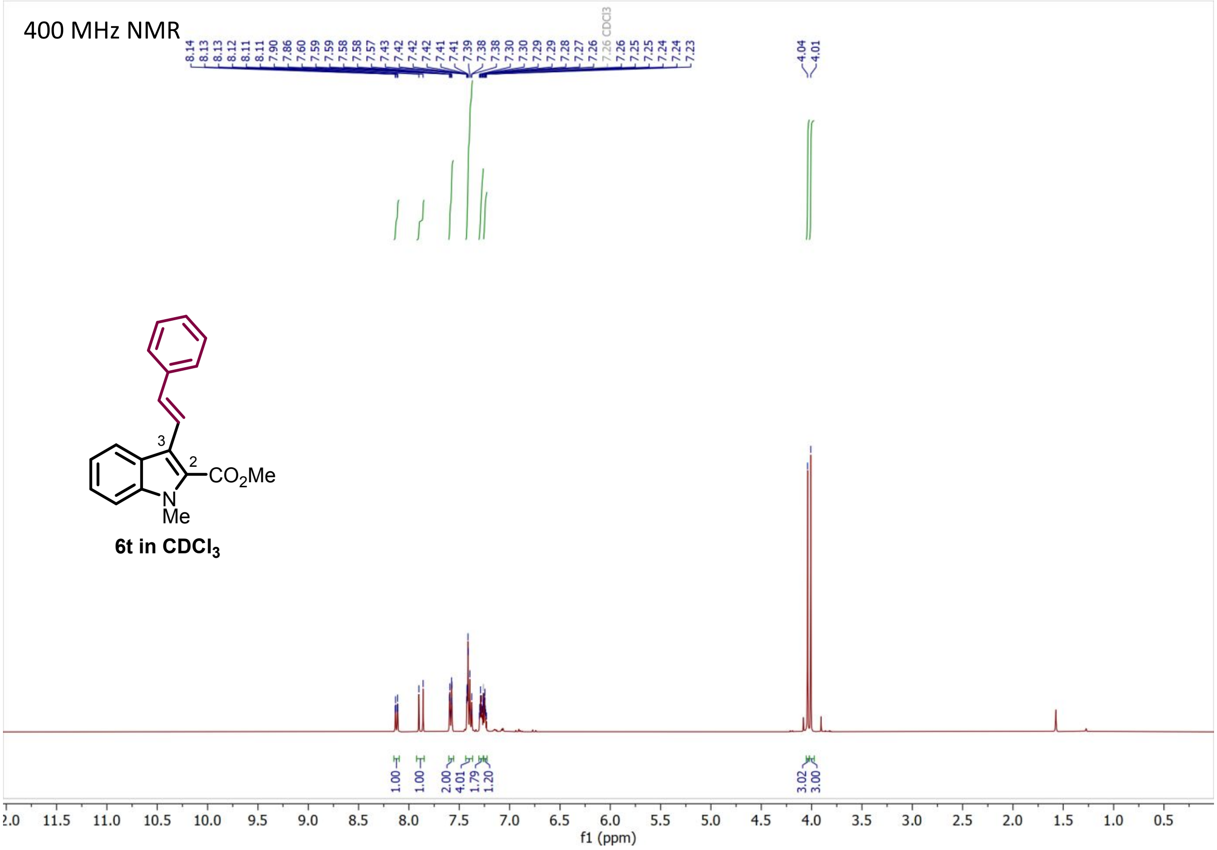
400 MHz NMR
8.14
8.13
8.13
8.12
8.11
8.11
7.90
7.86
7.60
7.59
7.59
7.58
7.58
7.57
7.43
7.42
7.42
7.42
7.41
7.39
7.38
7.38
7.30
7.30
7.29
7.29
7.28
7.27
7.26 CDCl3
7.26
7.26
7.25
7.25
7.24
7.24
7.23
4.04
4.01
3
2
CO2Me
N
Me
6t in CDCl3
1.00
1.00
2.00
4.01
1.79
1.20
3.02
3.00
f1 (ppm)

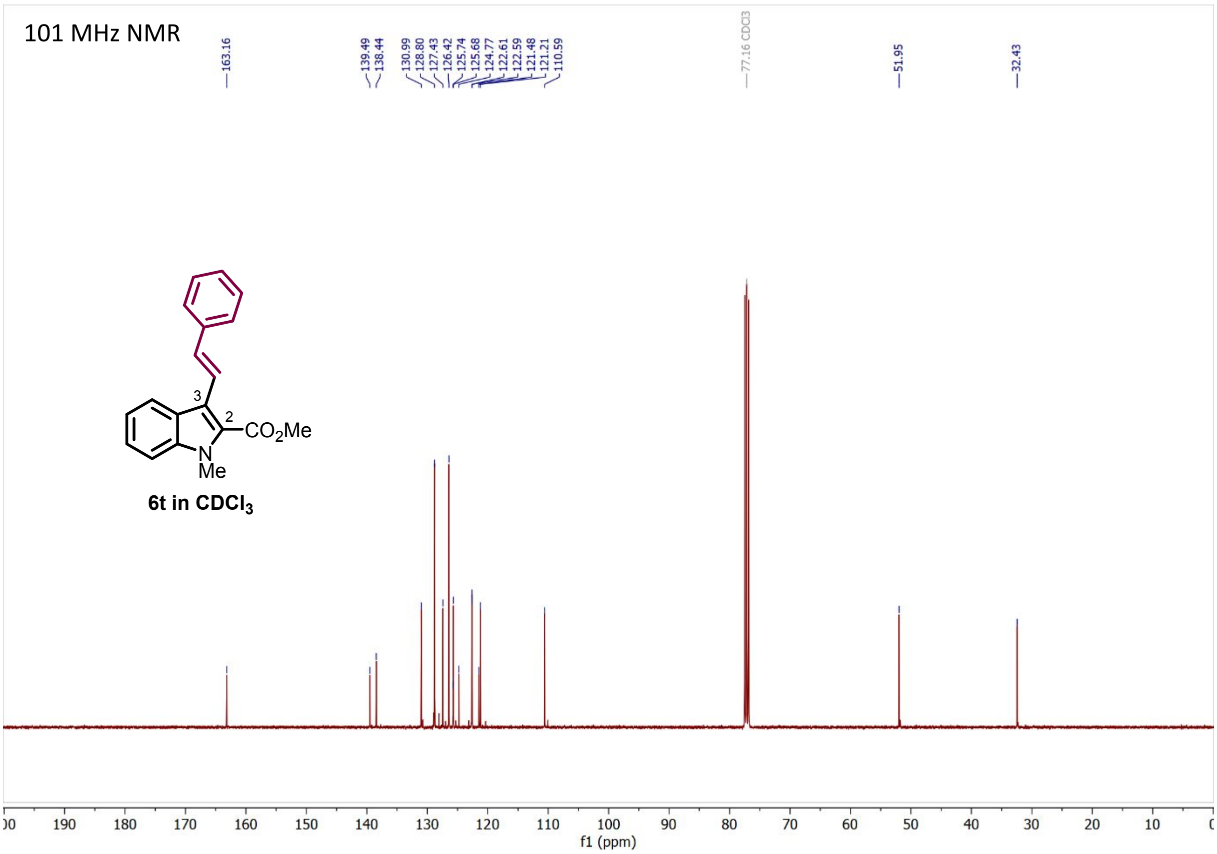
101 MHz NMR
163.16
139.49
138.44
130.99
128.80
127.43
126.42
125.74
125.68
124.77
122.61
122.59
121.48
121.21
110.59
77.16 CDCl3
51.95
32.43
3
2
CO2Me
N
Me
6t in CDCl3
f1 (ppm)

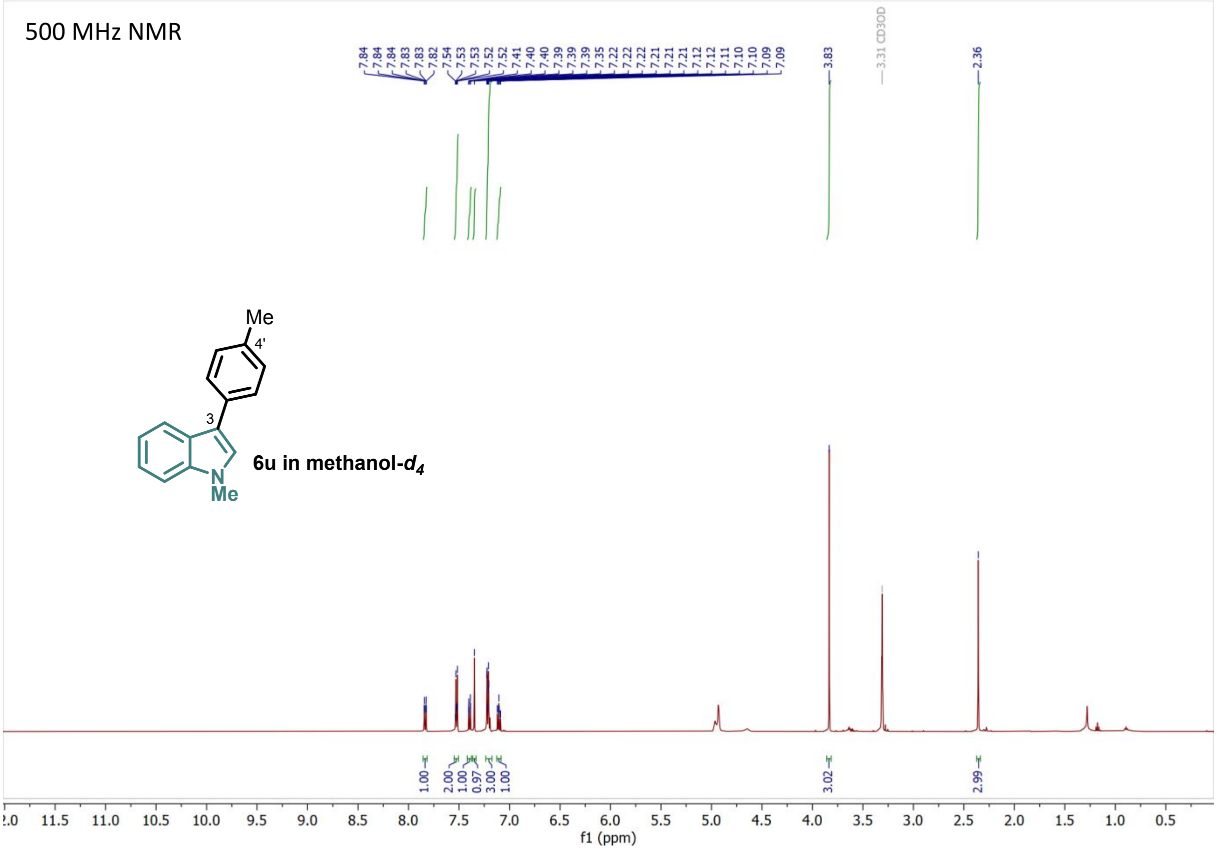
500 MHz NMR
7.84
7.84
7.84
7.83
7.83
7.82
7.54
7.53
7.53
7.52
7.52
7.52
7.41
7.40
7.40
7.39
7.39
7.39
7.35
7.22
7.22
7.22
7.22
7.21
7.21
7.21
7.12
7.12
7.11
7.10
7.10
7.09
7.09
3.83
3.31 CD3OD
2.36
Me
4'
3
N
Me
6u in methanol-d_4
1.00
2.00
1.00
0.97
3.00
1.00
3.02
2.99
f1 (ppm)

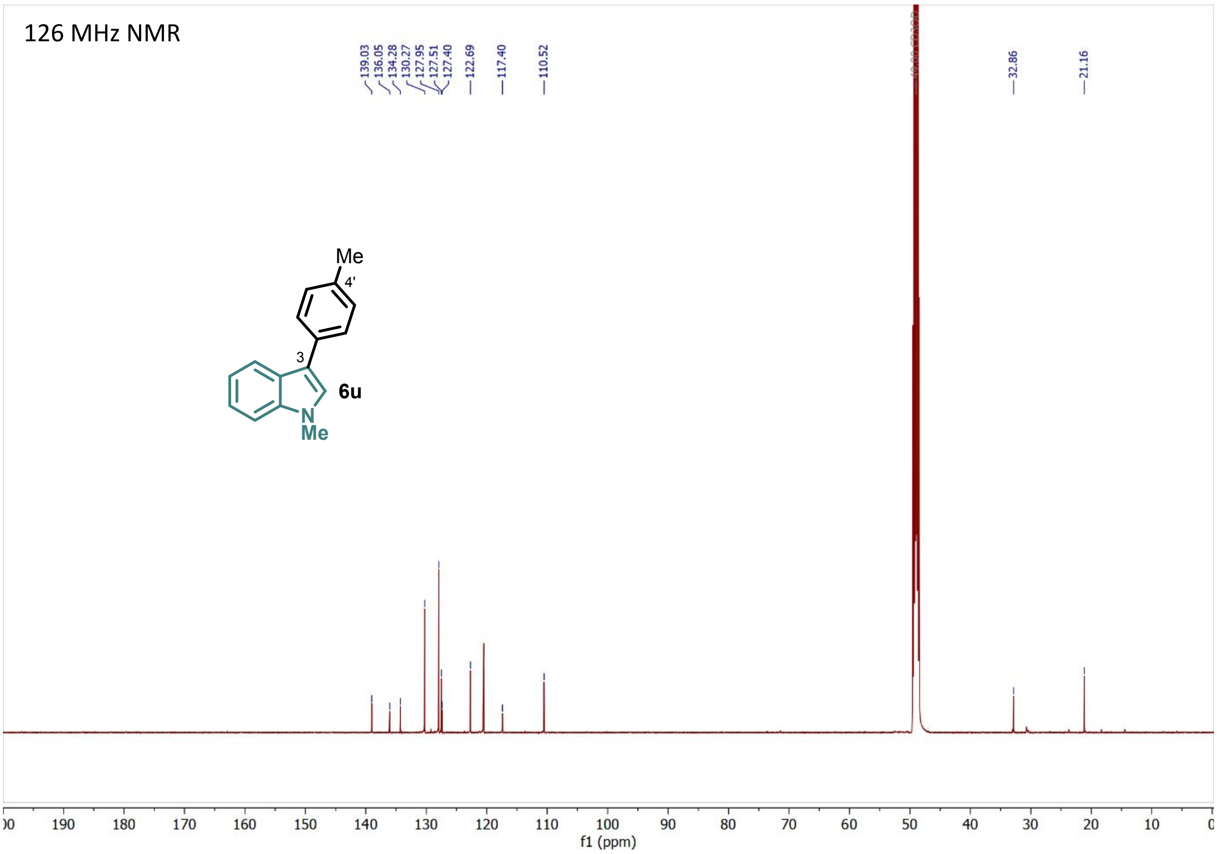
126 MHz NMR
139.03
136.05
134.28
130.27
127.95
127.51
127.40
122.69
117.40
110.52
32.86
21.16
Me
4'
3
6u
N
Me
f1 (ppm)

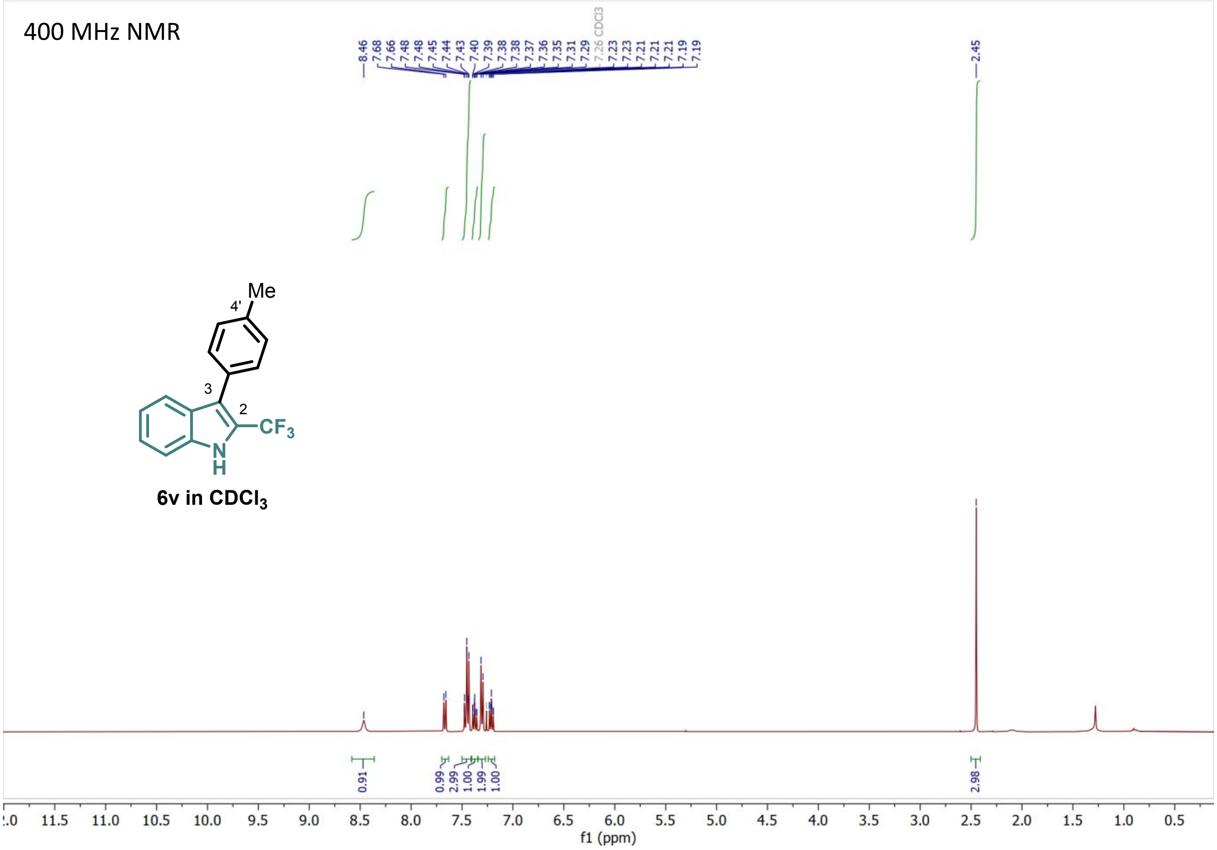
400 MHz NMR
6v in CDCl3
f1 (ppm)

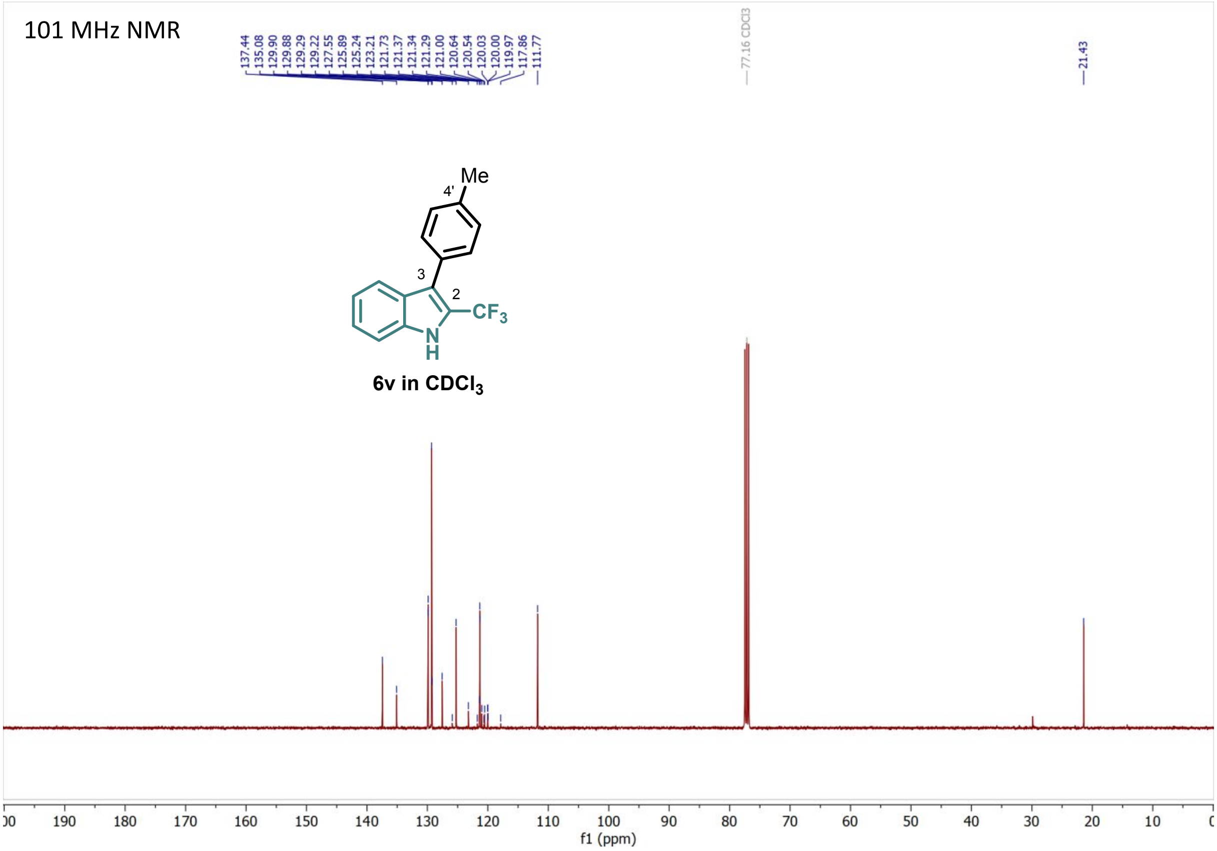
101 MHz NMR
6v in CDCl3
f1 (ppm)

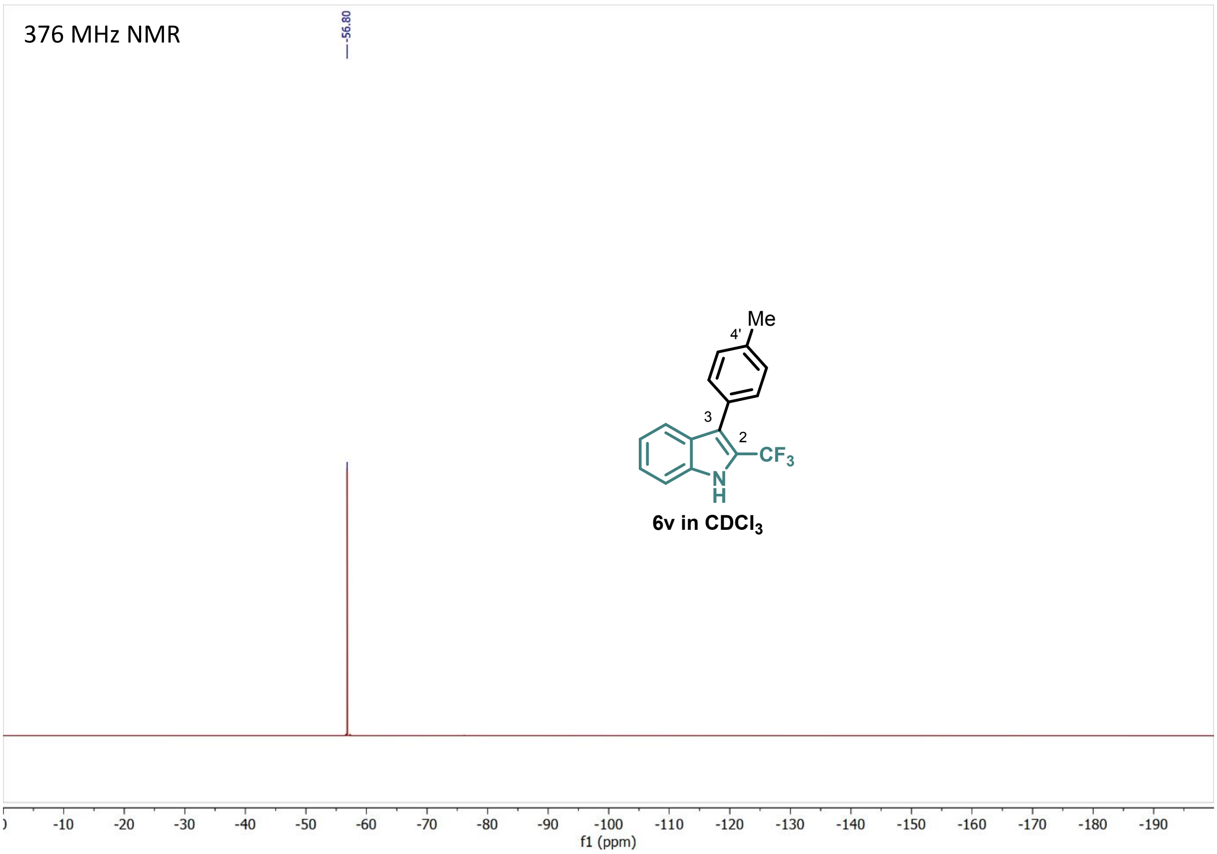
376 MHz NMR
-56.80
Me
4'
3
2
CF3
N
H
6v in CDCl3
-10
-20
-30
-40
-50
-60
-70
-80
-90
-100
-110
-120
-130
-140
-150
-160
-170
-180
-190
f1 (ppm)

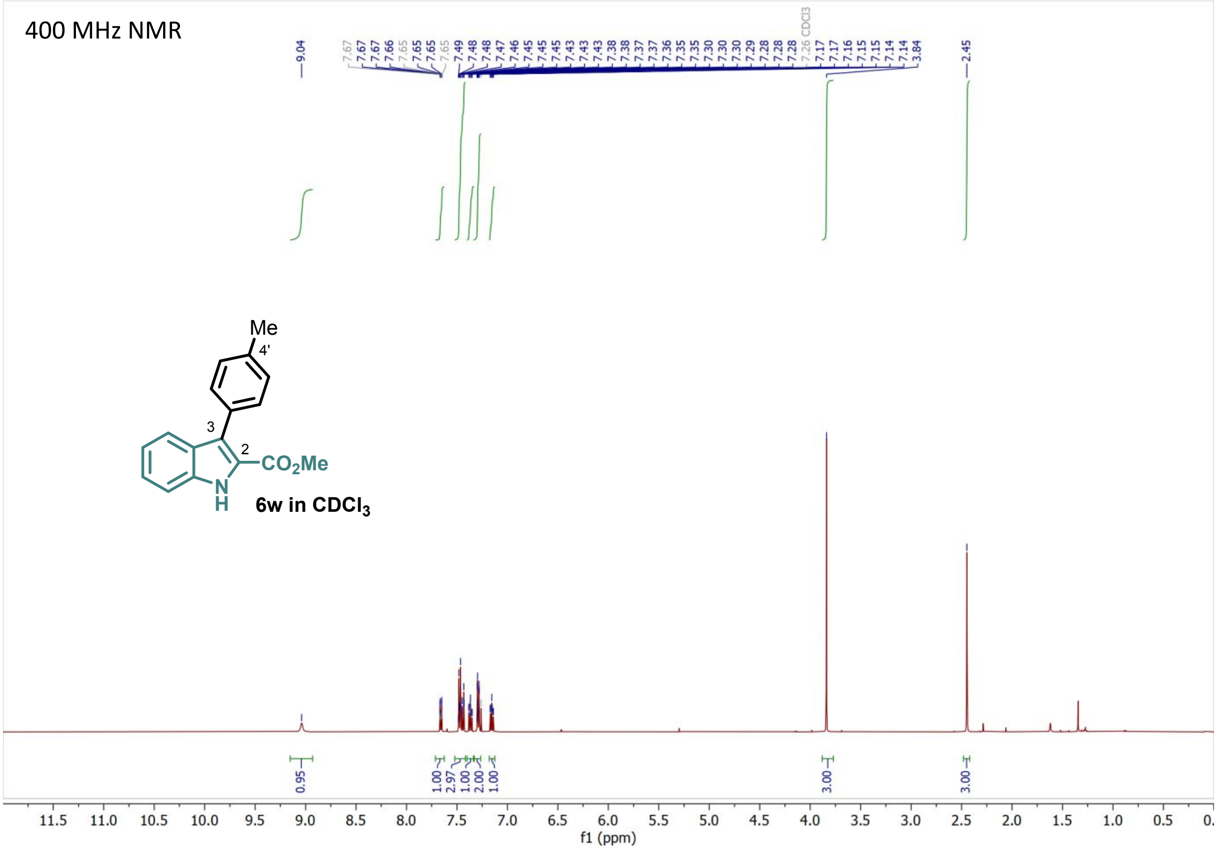
400 MHz NMR
Me
4'
3
2
CO2Me
N
H
6w in CDCl3
f1 (ppm)

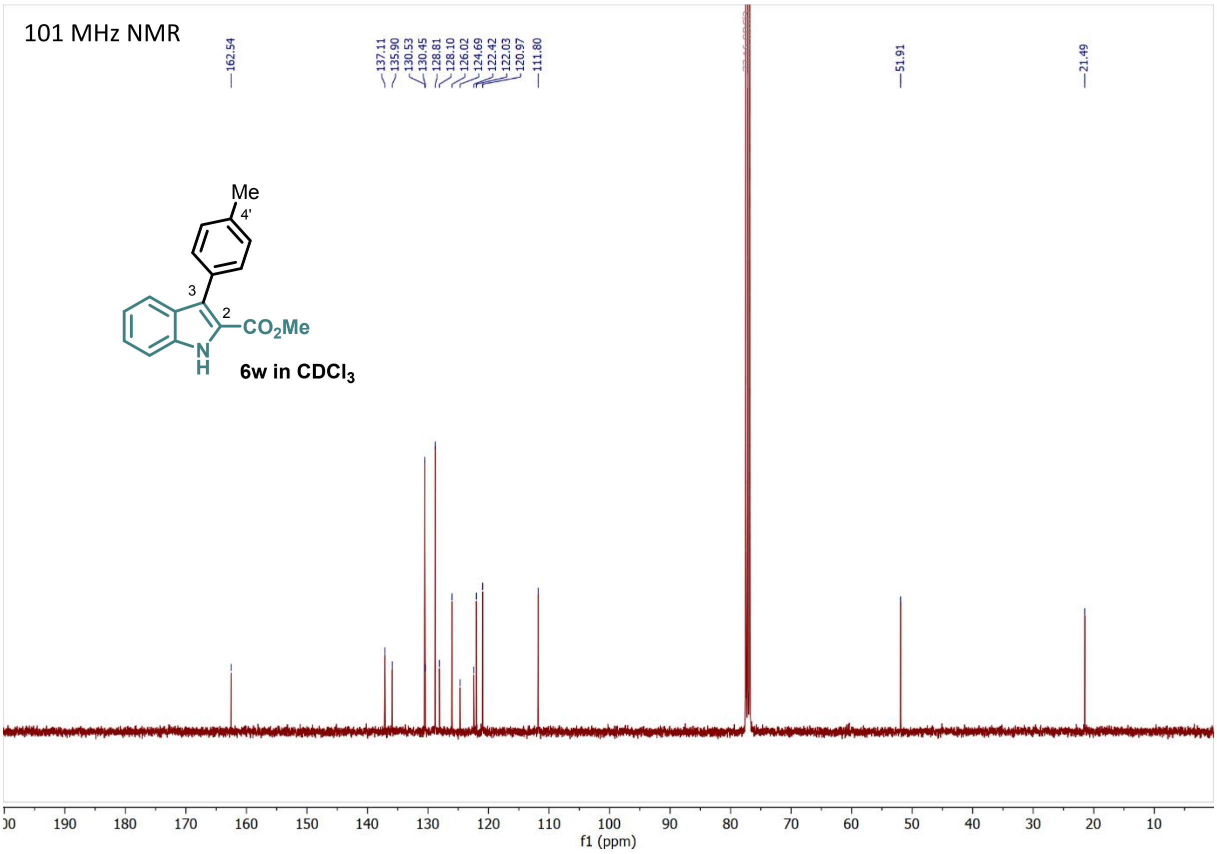
101 MHz NMR
162.54
137.11
135.90
130.53
130.45
128.81
128.10
126.02
124.69
122.42
122.03
120.97
111.80
51.91
21.49
Me
4'
3
2
CO2Me
N
H
6w in CDCl3
f1 (ppm)

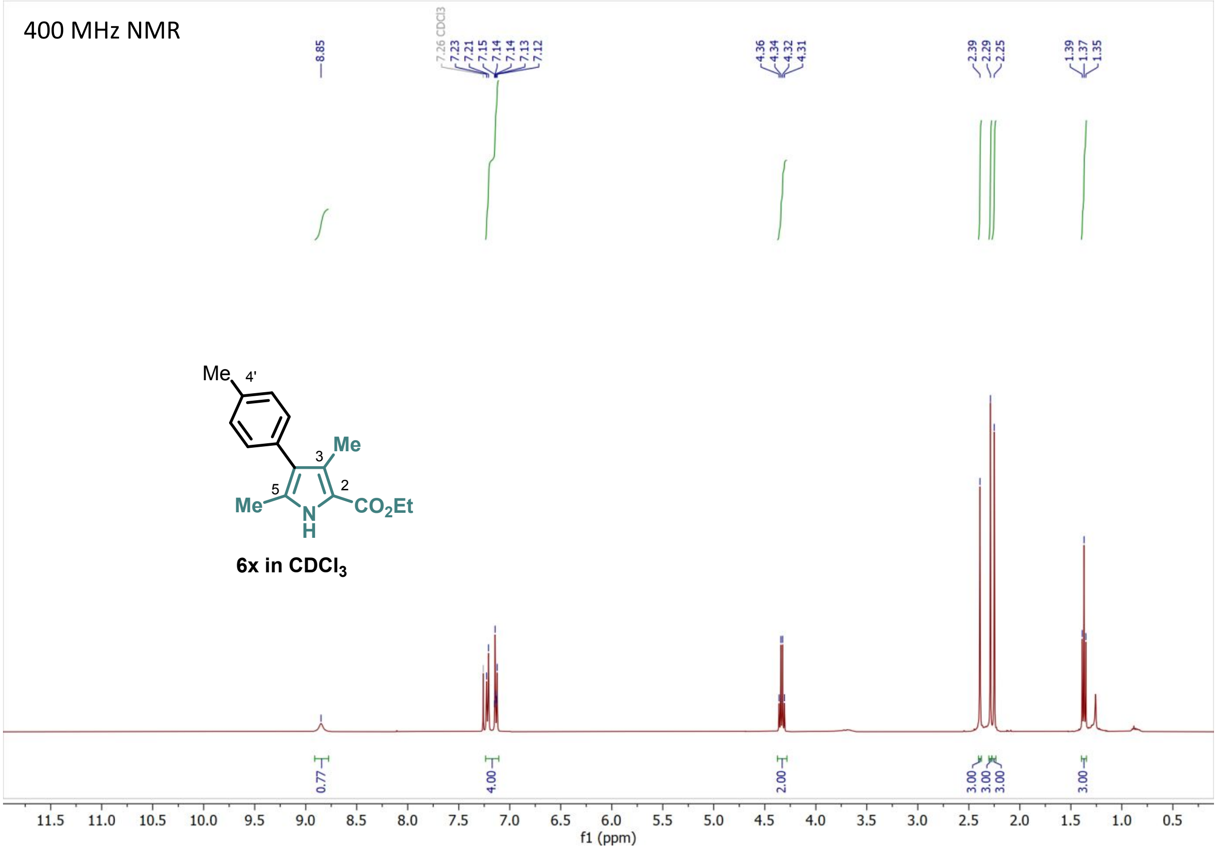
400 MHz NMR
8.85
7.26 CDCl3
7.23
7.21
7.15
7.14
7.14
7.13
7.12
4.36
4.34
4.32
4.31
2.39
2.29
2.25
1.39
1.37
1.35
Me
4'
3
Me
5
2
Me
N
H
CO_2Et
6x in $CDCl_3$
0.77
4.00
2.00
3.00
3.00
3.00
3.00
f1 (ppm)

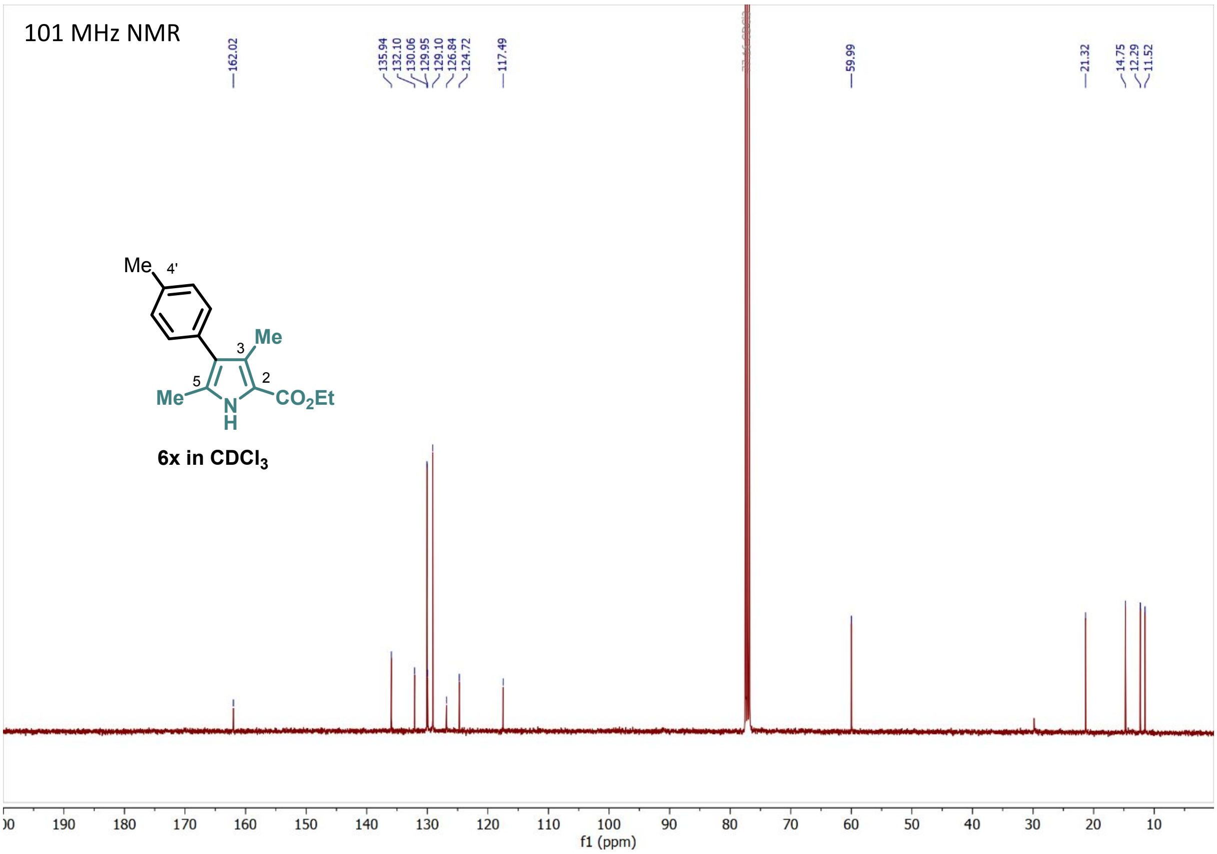
101 MHz NMR
162.02
135.94
132.10
130.06
129.95
129.10
126.84
124.72
117.49
59.99
21.32
14.75
12.29
11.52
Me
4'
3
Me
5
2
Me
N
H
CO_2Et
6x in $CDCl_3$
f1 (ppm)

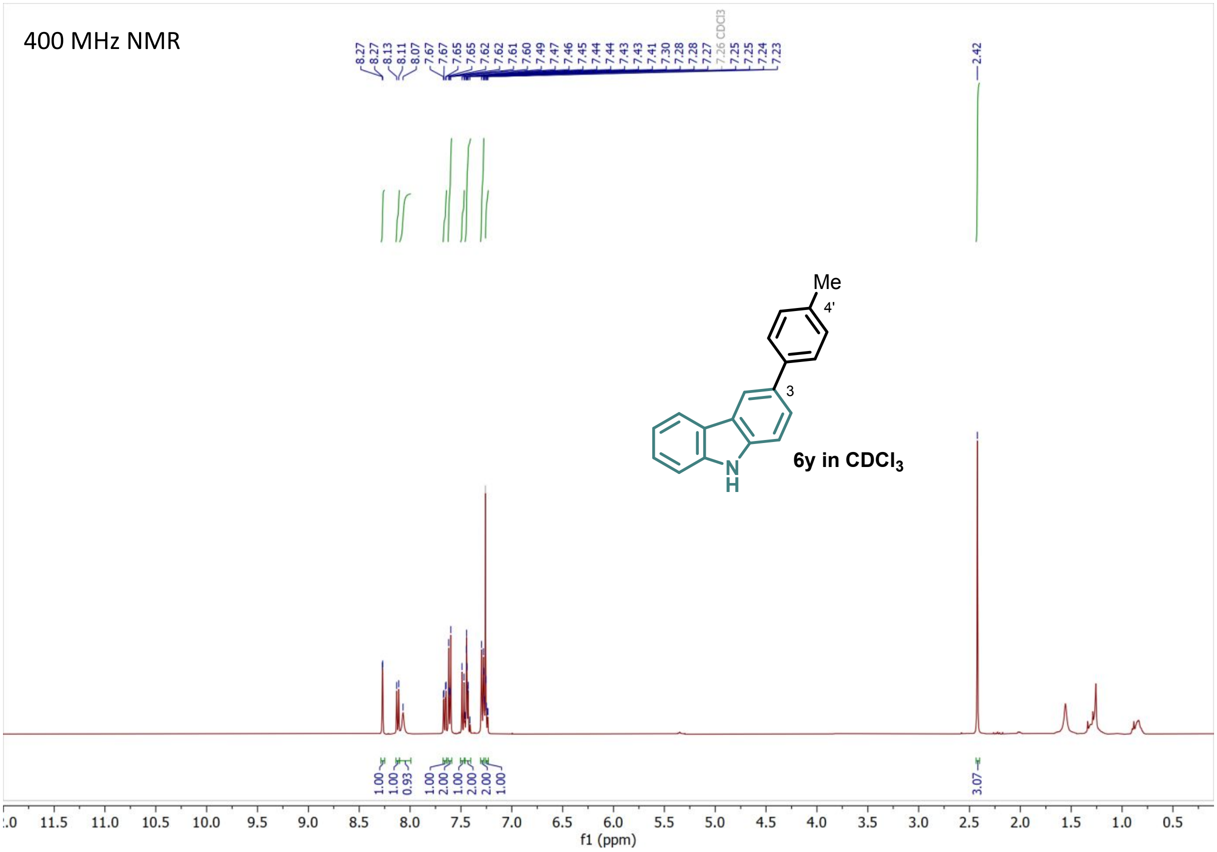
400 MHz NMR
8.27
8.27
8.13
8.11
8.07
7.67
7.65
7.65
7.62
7.62
7.61
7.60
7.49
7.47
7.46
7.45
7.44
7.44
7.43
7.43
7.41
7.30
7.28
7.28
7.27
7.26 CDCl3
7.25
7.25
7.24
7.23
2.42
Me
4'
3
N
H
6y in CDCl3
1.00
1.00
0.93
1.00
2.00
1.00
2.00
2.00
2.00
1.00
3.07
f1 (ppm)

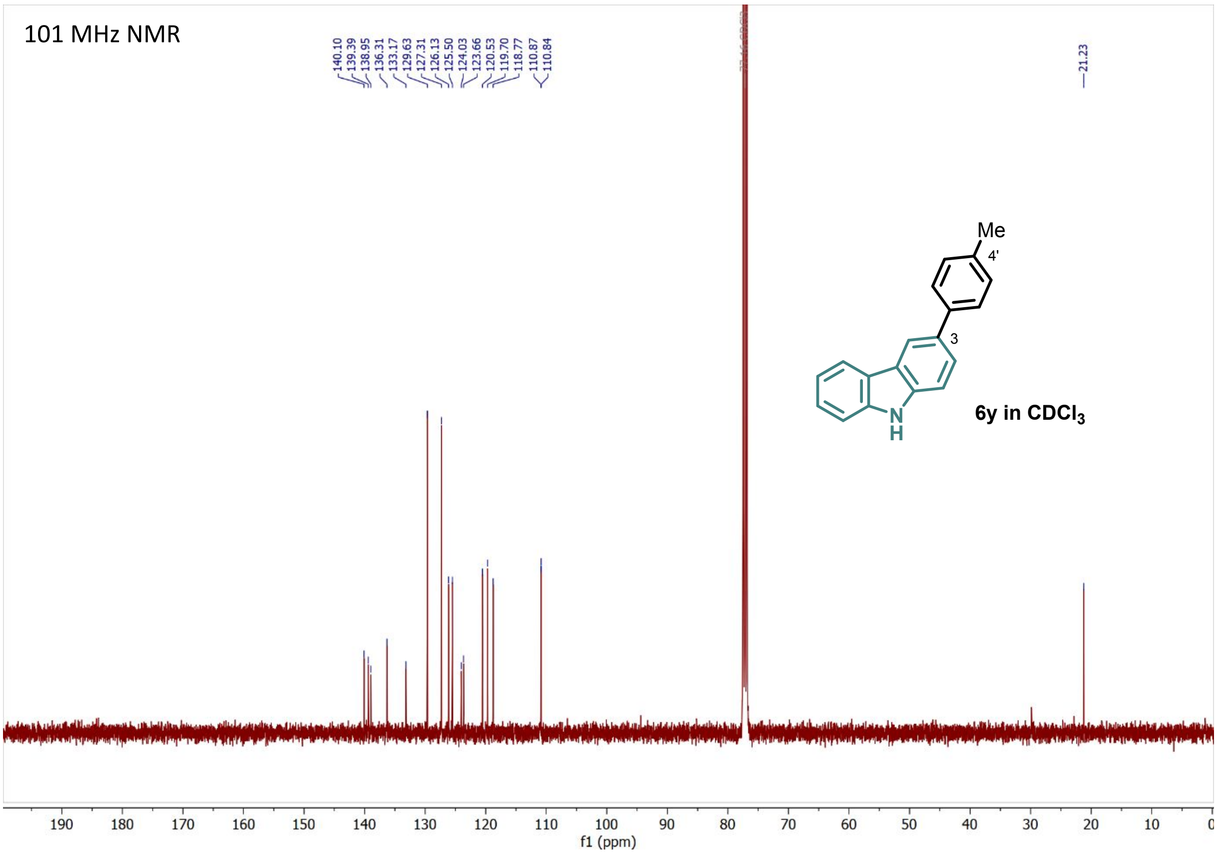
101 MHz NMR
140.10
139.39
138.95
136.31
133.17
129.63
127.31
126.13
125.50
124.03
123.66
120.53
119.70
118.77
110.87
110.84
21.23
Me
4'
3
N
H
6y in CDCl3
f1 (ppm)

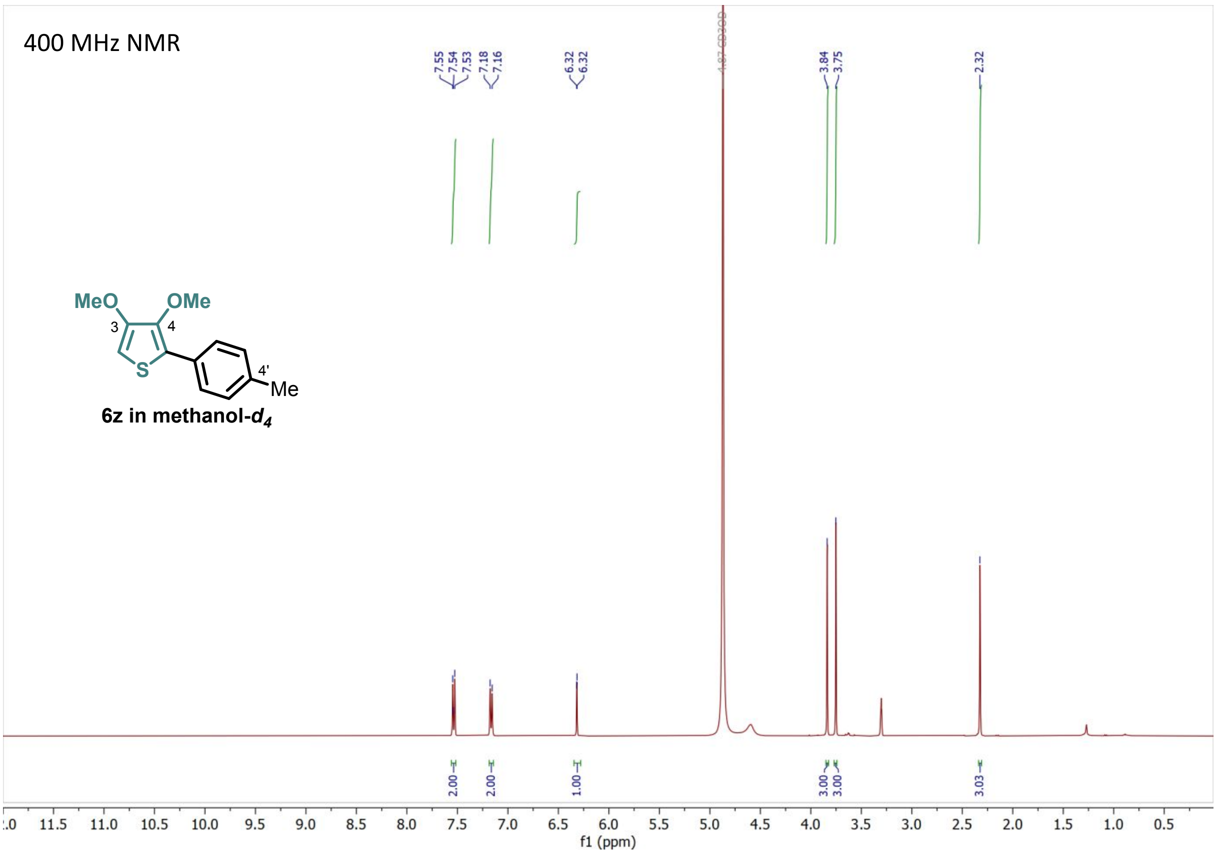
400 MHz NMR
7.55
7.54
7.53
7.18
7.16
6.32
6.32
3.84
3.75
2.32
MeO
OMe
3
4
S
4'
Me
6z in methanol-d_4
2.00
2.00
1.00
3.00
3.00
3.03
f1 (ppm)

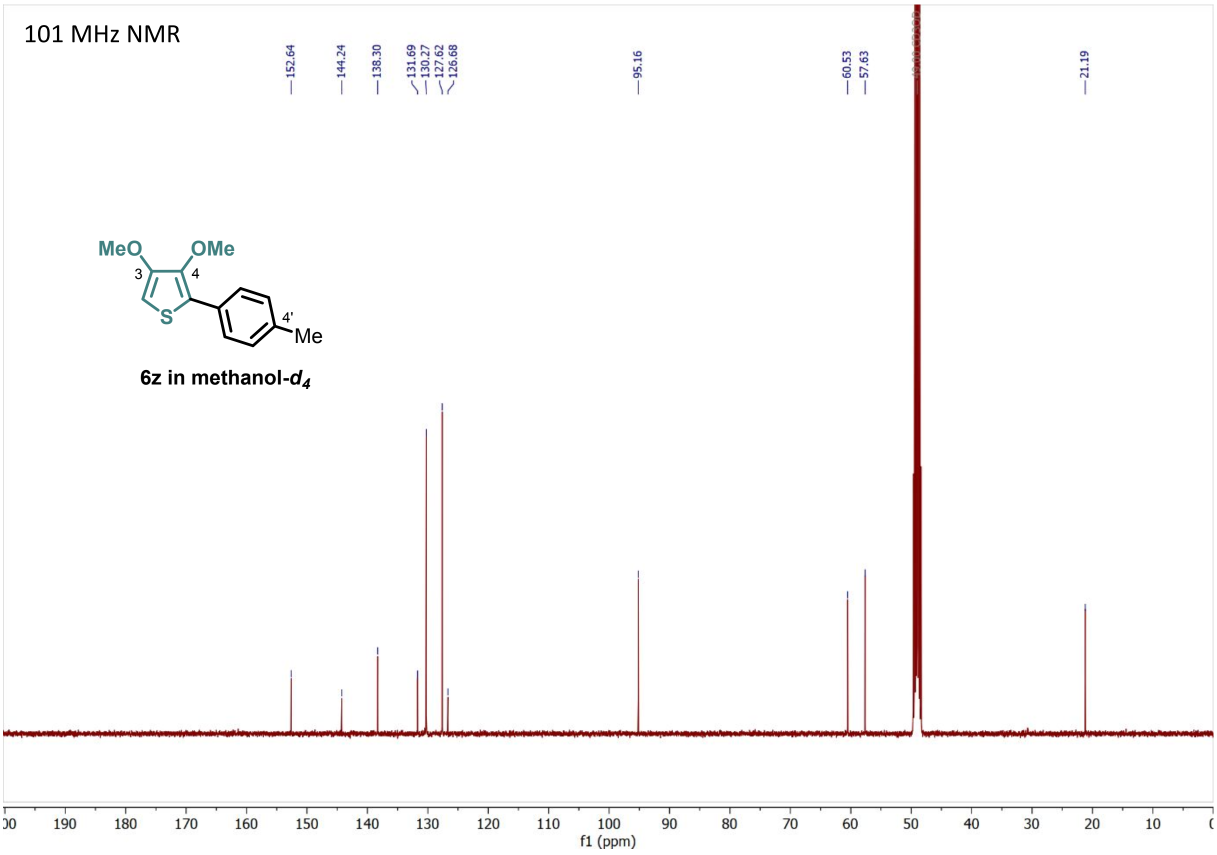
101 MHz NMR
152.64
144.24
138.30
131.69
130.27
127.62
126.68
95.16
60.53
57.63
21.19
MeO
OMe
3
4
S
4'
Me
6z in methanol-d_4
f1 (ppm)

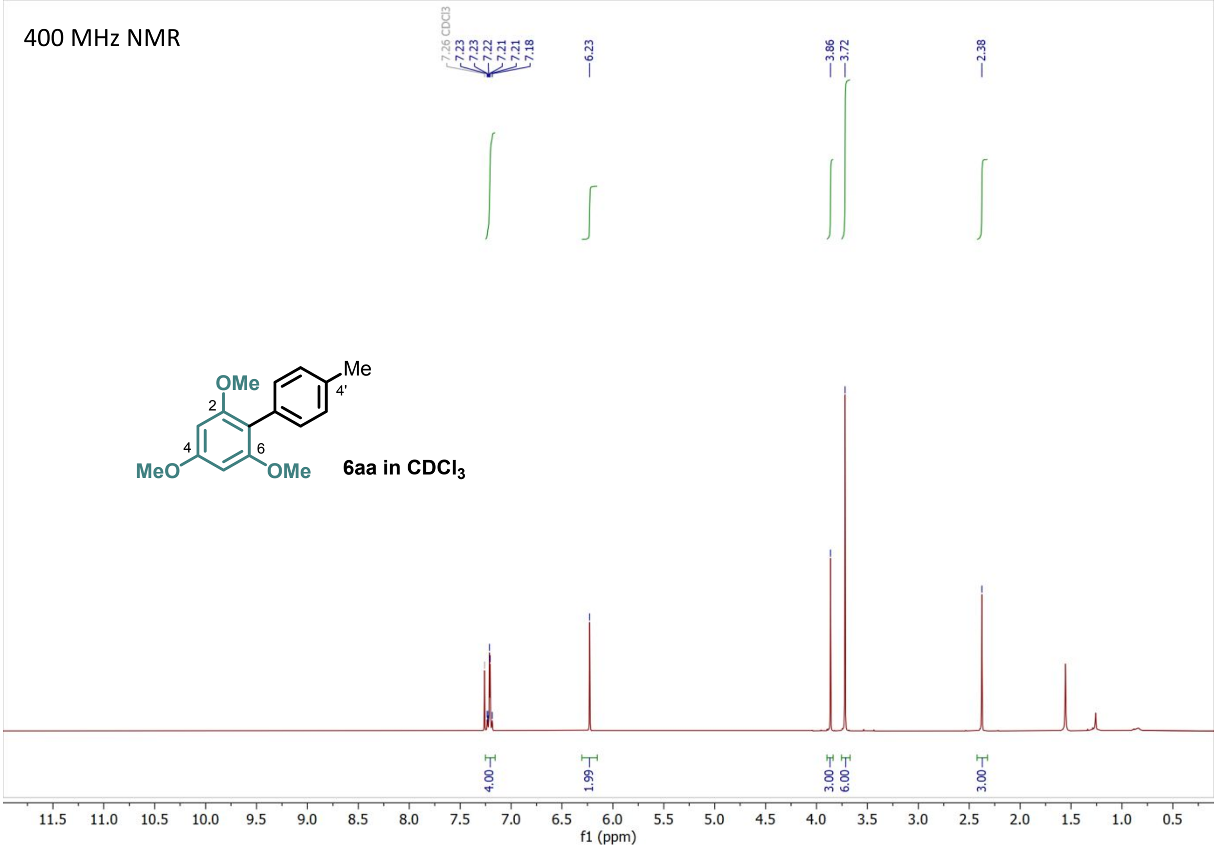
400 MHz NMR
7.26 CDCl3
7.23
7.23
7.22
7.21
7.21
7.18
6.23
3.86
3.72
2.38
OMe
Me
4'
2
4
6
MeO
OMe
6aa in $CDCl_3$
4.00
1.99
3.00
6.00
3.00
11.5
11.0
10.5
10.0
9.5
9.0
8.5
8.0
7.5
7.0
6.5
6.0
5.5
5.0
4.5
4.0
3.5
3.0
2.5
2.0
1.5
1.0
0.5
f1 (ppm)

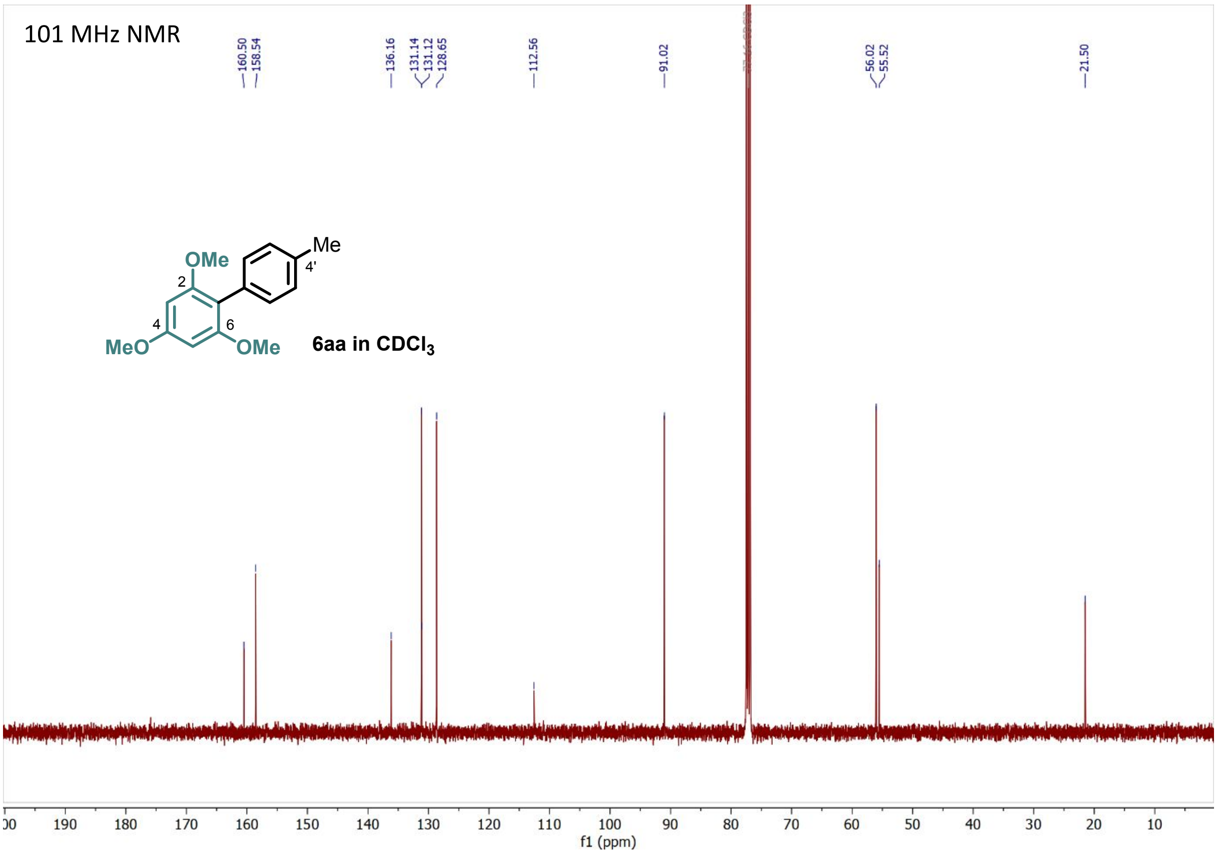
101 MHz NMR
160.50
158.54
136.16
131.14
131.12
128.65
112.56
91.02
56.02
55.52
21.50
OMe
Me
4'
2
4
6
MeO
OMe
6aa in $CDCl_3$
190
180
170
160
150
140
130
120
110
100
90
80
70
60
50
40
30
20
10
f1 (ppm)

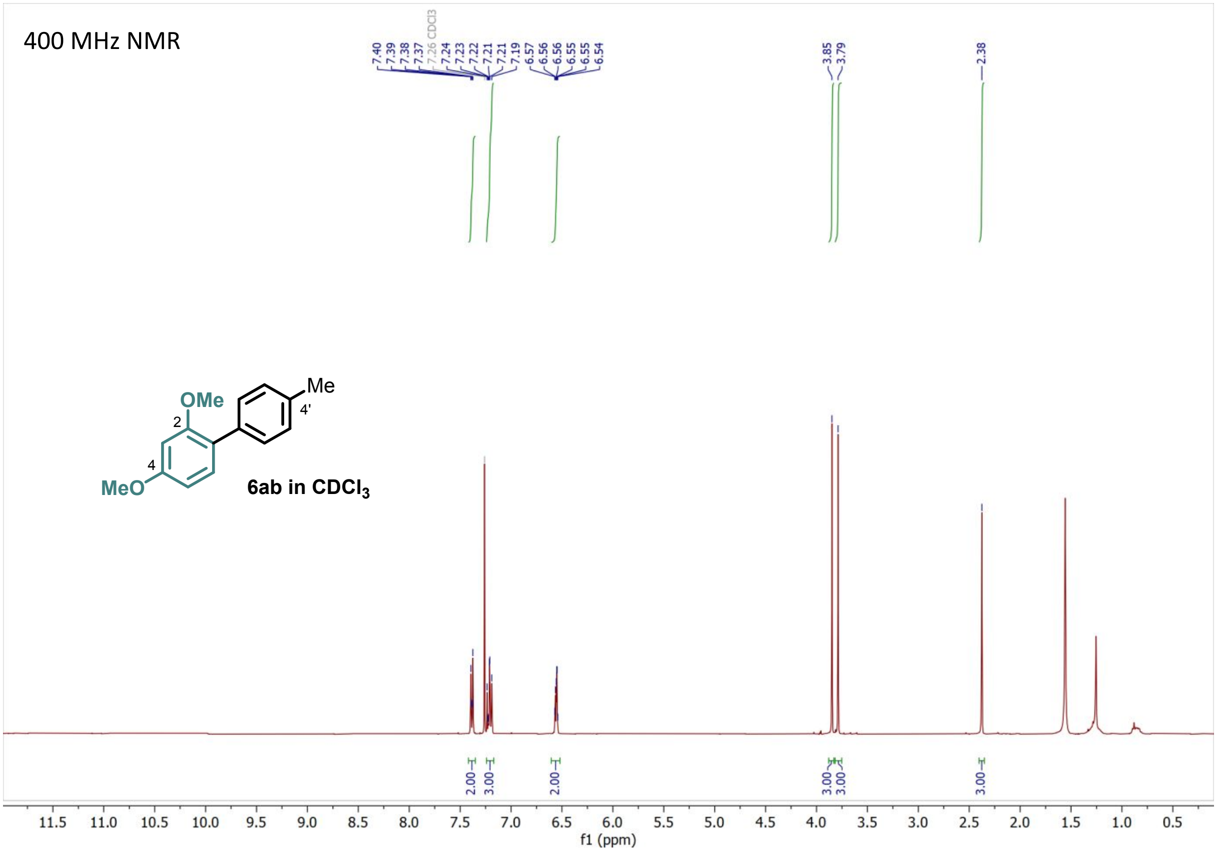
400 MHz NMR
7.40
7.39
7.38
7.37
7.26 CDCl3
7.24
7.23
7.22
7.21
7.21
7.21
7.19
6.57
6.56
6.56
6.55
6.55
6.54
3.85
3.79
2.38
OMe
Me
2
4'
4
MeO
6ab in CDCl3
2.00
3.00
2.00
3.00
3.00
3.00
11.5
11.0
10.5
10.0
9.5
9.0
8.5
8.0
7.5
7.0
6.5
6.0
5.5
5.0
4.5
4.0
3.5
3.0
2.5
2.0
1.5
1.0
0.5
f1 (ppm)

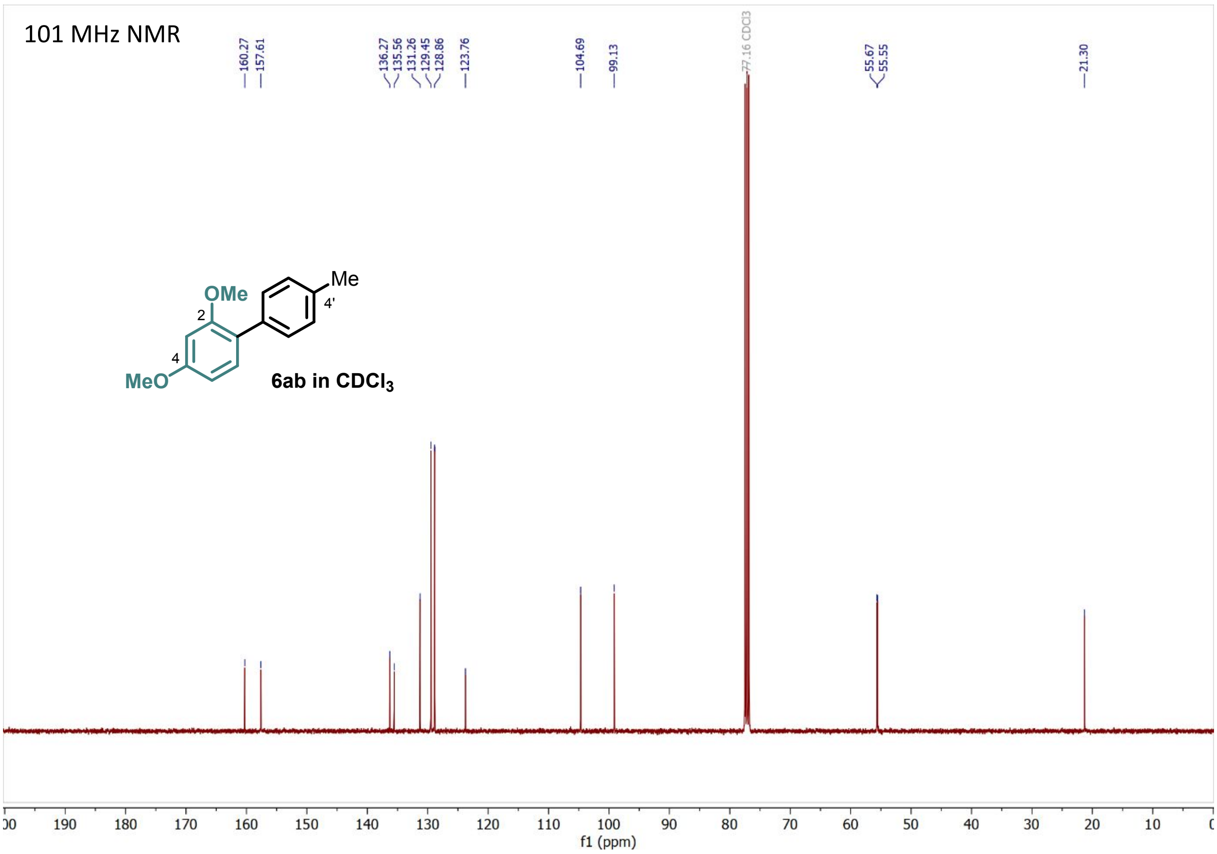
101 MHz NMR
160.27
157.61
136.27
135.56
131.26
129.45
128.86
123.76
104.69
99.13
77.16 CDCl3
55.67
55.55
21.30
OMe
Me
2
4'
4
MeO
6ab in CDCl3
190
180
170
160
150
140
130
120
110
100
90
80
70
60
50
40
30
20
10
f1 (ppm)

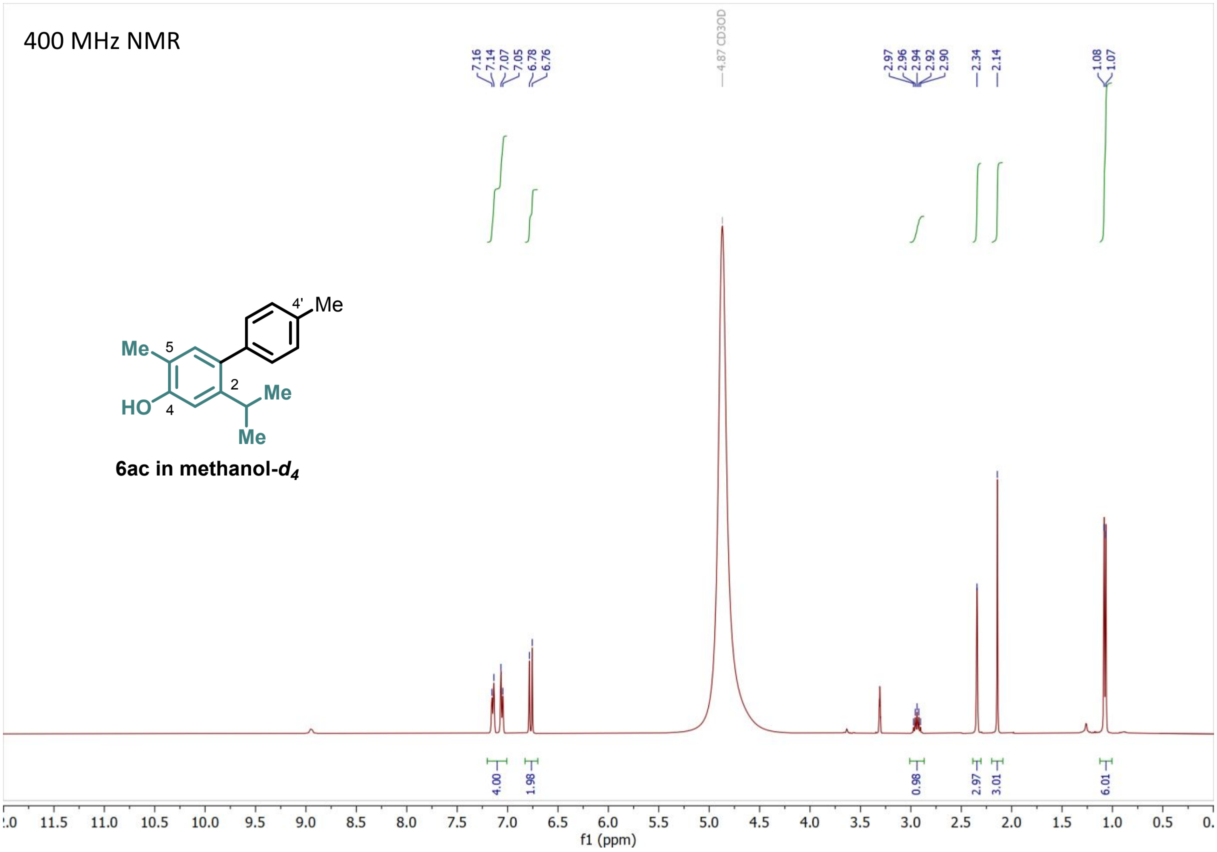

400 MHz NMR
7.16
7.14
7.07
7.05
6.78
6.76
4.87 CD3OD
2.97
2.96
2.94
2.92
2.90
2.34
2.14
1.08
1.07
Me
4'
Me
5
2
Me
HO
4
Me
6ac in methanol-d4
4.00
1.98
0.98
2.97
3.01
6.01
f1 (ppm)

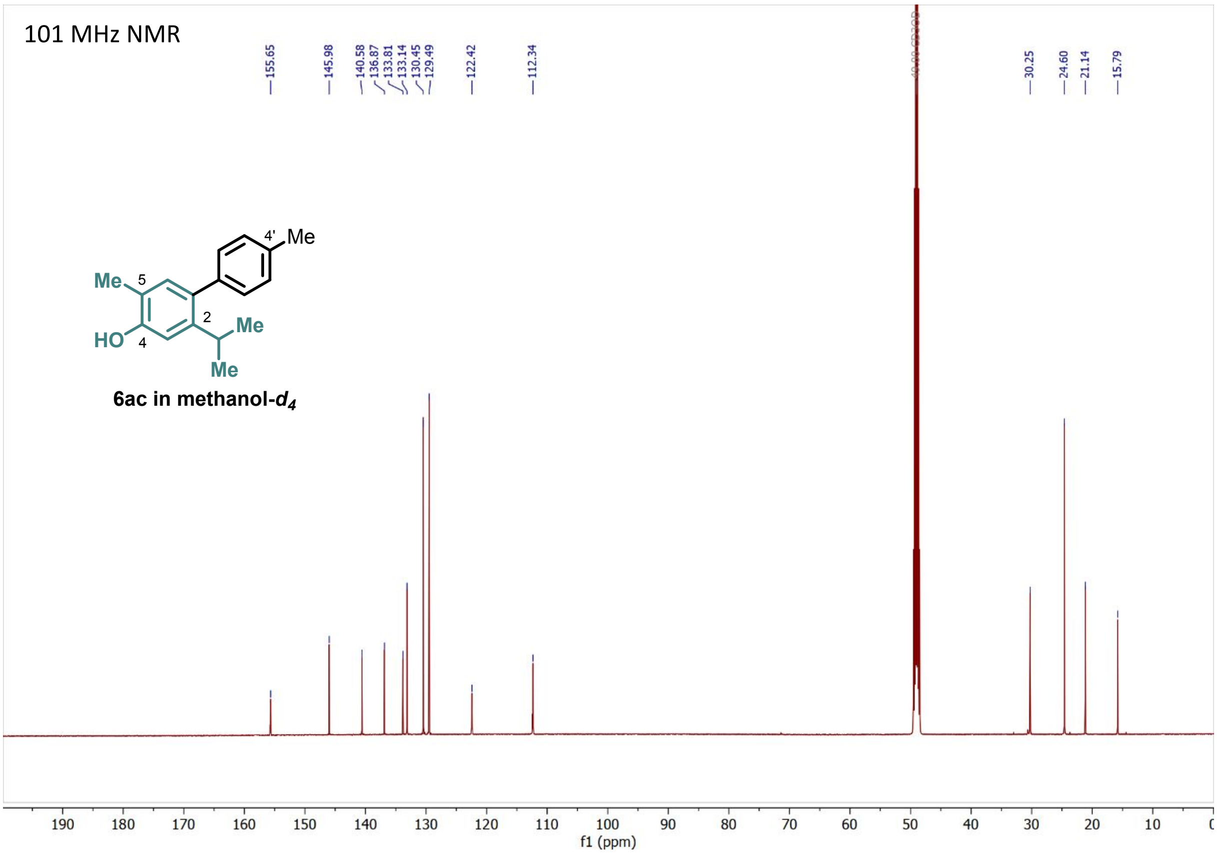

101 MHz NMR
155.65
145.98
140.58
136.87
133.81
133.14
130.45
129.49
122.42
112.34
30.25
24.60
21.14
15.79
Me
4'
Me
5
2
Me
HO
4
Me
6ac in methanol-d4
f1 (ppm)

7. References

[1] a) S. Diethelm, R. Teufel, L. Kaysser, B. S. Moore, *Angew. Chem. Int. Ed.* **2014**, *53*, 11023-11026; b) D. R. Morris, L. P. Hager, *J. Biol. Chem.* **1966**, *241*, 1763-1768.

[2] a) G. V. Los, L. P. Encell, M. G. McDougall, D. D. Hartzell, N. Karassina, C. Zimprich, M. G. Wood, R. Learish, R. F. Ohana, M. Urh, *ACS Chem. Biol.* **2008**, *3*, 373-382; b) G. Los, A. Darzins, N. Karassina, C. Zimprich, R. Learish, M. McDougall, L. Encell, R. Friedman-Ohana, M. Wood, G. Vidugiris, *Cell Notes* **2005**, *11*, 2-6; c) G. V. Los, K. Wood, in *High Content Screening*, Springer, **2007**, pp. 195-208.

[3] a) P. e. Smith, R. I. Krohn, G. Hermanson, A. Mallia, F. Gartner, M. Provenzano, E. Fujimoto, N. Goeke, B. Olson, D. Klenk, *Anal. Biochem.* **1985**, *150*, 76-85; b) J. M. Walker, in *The protein protocols handbook*, Springer, **2009**, pp. 11-15.

[4] P. Dydio, J. N. Reek, *Angew. Chem.* **2013**, *125*, 3970-3974.

[5] B. Malapel-Andrieu, J.-Y. Mérour, *Tetrahedron* **1998**, *54*, 11079-11094.

[6] M. Sechi, M. Derudas, R. Dallocchio, A. Dessì, A. Bacchi, L. Sannia, F. Carta, M. Palomba, O. Ragab, C. Chan, *J. Med. Chem.* **2004**, *47*, 5298-5310.

[7] R. Gattu, S. Bhattacharjee, K. Mahato, A. T. Khan, *Org. Biomol. Chem.* **2018**, *16*, 3760-3770.

[8] T. Konno, J. Chae, T. Ishihara, H. Yamanaka, *J. Org. Chem.* **2004**, *69*, 8258-8265.

[9] J. Ni, Y. Jiang, Z. An, R. Yan, *Org. Lett.* **2018**, *20*, 1534-1537.

[10] S. Chakraborty, J. Ahmed, B. K. Shaw, A. Jose, S. K. Mandal, *Chem. Eur. J.* **2018**, *24*, 17651-17655.

[11] R. J. Key, J. M. M. Tengco, M. D. Smith, A. K. Vannucci, *Organometal.* **2019**, *38*, 2007-2014.
